# Pre‐conception weight loss interventions in women with polycystic ovary syndrome and the effect on perinatal outcomes: A quantitative synthesis of surrogate outcomes

**DOI:** 10.1111/dom.70116

**Published:** 2025-10-01

**Authors:** Amelia Fernandes, Adrienne Gordon, Arianne Sweeting

**Affiliations:** ^1^ Department of Endocrinology Royal Prince Alfred Hospital Sydney New South Wales Australia; ^2^ Faculty of Medicine and Health University of Sydney, Camperdown Sydney New South Wales Australia; ^3^ Charles Perkins Centre University of Sydney, Camperdown Sydney New South Wales Australia; ^4^ Department of Neonatology Royal Prince Alfred Hospital New South Wales Australia

**Keywords:** bariatric surgery, lifestyle, obesity, overweight, pharmacotherapy, polycystic ovary syndrome, weight loss

## Abstract

**Aims:**

Polycystic ovary syndrome (PCOS) guidelines recommend weight optimisation to improve anthropometric, metabolic and androgenic complications, but data is lacking for pregnancy outcomes.

**Materials and Methods:**

We aimed to assess which weight loss interventions improve pregnancy outcomes for women with PCOS and overweight or obesity. A systematic search of Embase, Medline (Ovid) and the Cochrane Clinical Trials Registry for English language articles from database inception until 22 July 2024 was conducted. We included weight loss intervention randomised controlled trials (RCTs) for women with PCOS and body mass index (BMI) ≥25 kg/m^2^. Primary outcomes were pregnancy rates, live births and miscarriages. Secondary outcomes were anthropometric, androgenic, metabolic and other perinatal complications.

**Results:**

Of 9010 articles, 7077 abstracts were screened and 37 RCTs included. One study reported increased pregnancy rates with a 12‐month lifestyle intervention versus standard care (23.3%–26.7% vs. 16.7%) (low certainty) but no difference in live birth rate, time to conception or antenatal outcomes. Lifestyle interventions reduced BMI by −0.34 kg/m^2^ (−0.65 to −0.02) on quantitative meta‐analysis. Limitations include outcome measure heterogeneity, intervention heterogeneity, inconsistent definitions and low use of core outcome sets.

**Conclusions:**

Limited data on antenatal outcomes highlight a knowledge gap amongst a group at high perinatal risk.

## 1 INTRODUCTION

Polycystic ovary syndrome (PCOS) is a common endocrine condition with an estimated global prevalence of 8%–13% amongst women of reproductive age.^1^ The diagnosis is based on consensus‐based criteria: two or more of clinical or biochemical hyperandrogenism, ovulatory dysfunction or polycystic ovarian ultrasound morphology.^2^, ^3^ PCOS is associated with cardiometabolic, androgenic and psychological complications, and a higher risk of reproductive and perinatal sequelae.^2^, ^3^ Independent of body mass index (BMI), women with PCOS experience greater risk of miscarriage (odds ratio [OR]: 1.49; 95% confidence interval [CI]: 1.20–1.85), gestational diabetes (GDM) (OR: 2.41; 95% CI: 1.95–2.99), gestational hypertension (OR: 2.20; 95% CI: 1.81, 2.69), pre‐eclampsia (OR: 2.30; 95% CI: 1.87–2.82), caesarean delivery (OR: 1.23; 95% CI: 1.06–1.43), preterm birth (OR: 1.93; 95% CI: 1.45–2.57) and neonatal intensive care unit admission (NICU) (OR: 2.32; 95% CI: 1.40–3.85).^4^, ^5^

Obesity is prevalent in approximately 50%–80% of women with PCOS. Women with PCOS have a higher BMI (1.76 kg/m^2^; 95% CI 1.44–2.08) at conception versus women without PCOS.^4^ There is an additive risk of pregnancy complications with PCOS and BMI ≥25 kg/m^2^.^5^, ^6^, ^7^, ^8^, ^9^, ^10^ Conversely, women with PCOS of normal BMI have a lower miscarriage risk (0.64; 95% CI: 0.59–0.71) and increased live birth rate (1.39; 95% CI: 1.17–1.65) versus women with PCOS and BMI ≥25 kg/m^2^.^2^

International evidence‐based guidelines for PCOS recommend weight loss for BMI ≥25 kg/m^2^ using lifestyle and pharmacotherapy interventions or potentially bariatric surgery.^2^ Whether optimising pre‐conception BMI can improve later pregnancy outcomes is unknown, as is timing, duration or degree of weight loss.^2^ A 2019 Cochrane review on lifestyle interventions for women with PCOS at any age reported minimal data on pregnancy outcomes.^11^ While previous reviews have compared lifestyle or pharmacotherapy interventions, currently no comprehensive review exists assessing all lifestyle, pharmacological and surgical weight loss modalities for women with PCOS and BMI ≥25 kg/m^2^.^11^, ^12^

Therefore, this review aims to compare lifestyle, pharmacological and surgical weight loss therapies on pregnancy outcomes in women with PCOS and overweight/obesity.

## 2 MATERIALS AND METHODS

### 2.1 Study design

This review was reported according to the Preferred Reporting Items for Systematic Reviews and Meta‐analyses (PRISMA) checklist and registered with International Prospective Register of Systematic Reviews (CRD42022383443)^13^

### 2.2 Eligibility criteria

English language randomised controlled trials (RCTs) assessing women ≥18 years with both PCOS (based on the trial definition) and BMI ≥25 kg/m^2^.

Any weight loss intervention defined as any non‐pharmacological, pharmacological or surgical therapy to induce weight loss was included. Non‐pharmacological intervention included lifestyle, psychological, diet and exercise. Pharmacological included therapies with weight loss potential including orlistat, sibutramine, glucagon like peptide 1 (GLP‐1) receptor agonists and metformin. Surgical therapy included any bariatric surgery.

All published RCTs with multi‐arm or head‐to‐head active comparators, placebo or usual care were eligible.

Primary outcome measures were pregnancy rate, live birth rate and miscarriage. Secondary outcome measures were perinatal (time to conception, GDM, pre‐eclampsia, hypertension in pregnancy, stillbirth, preterm delivery and NICU admission) androgenic (menstrual regularity, ovulation, total testosterone, sex hormone‐binding globulin [SHBG], free androgen index [FAI], hirsutism), metabolic (blood pressure [BP], fasting glucose, insulin, homeostatic model assessment for insulin resistance [HOMA‐IR], Haemoglobin A1c (HbA1c), triglycerides and total, high‐density [HDL‐c], low‐density [LDL‐c] lipoprotein cholesterol) and anthropometric (weight, BMI, waist circumference, waist‐to‐hip ratio [WHR]; Supporting Information S1).

### 2.3 Information sources and search strategy

A systematic search (Supporting Information S2) was conducted in Cochrane, Embase and Medline (Ovid) from inception to July 2024. The decision for these databases was based on included studies from previously published reviews. The search was limited to the English language. Unpublished studies and grey literature were not included.

### 2.4 Selection process and data collection process

Articles were independently screened by A.F. and A.S. using Covidence. Conflicts were reviewed by A.G. who made the final decision. Included studies were cross‐checked on the Retraction Watch Database by A.F., A.S. and A.G.

### 2.5 Data items

Data were independently collected by A.F. and A.S. for study and participant characteristics, interventions and outcome data (Supporting Information S3).

### 2.6 Study risk of bias assessment

Study quality was independently assessed using the Cochrane ‘Risk of Bias’ version 2 tool by A.F. and A.S.^14^ If there were any disagreements, this was reviewed by A.G.

### 2.7 Effect measures and synthesis methods

Mean difference (MD) and standard deviation (SD) were calculated for continuous outcomes and OR with 95% CI for dichotomous outcomes. All data were analysed on an intention‐to‐treat (ITT) basis, and we conducted pairwise quantitative meta‐analyses comparing intervention categories using a random‐effects model. Several included studies featured multiple arms (e.g., pharmacotherapy, lifestyle intervention and combination), which may introduce statistical dependence due to shared comparators. While pairwise comparisons were assessed, we did not apply statistical methods to explicitly model the correlation between arms in multi‐arm trials. *I* ^2^ statistic assessed heterogeneity, with *I* ^2^ >50% indicating substantial heterogeneity. A two‐sided *p*‐value <0.05 was considered statistically significant. Data analysis was performed with Review Manager (Revman) 5.4.

### 2.8 Certainty assessment

The Grading of Recommendations Assessment, Development and Evaluation (GRADE) was used for grading the certainty of evidence.^15^

## 3 RESULTS

### 3.1 Study selection

**FIGURE 1:**
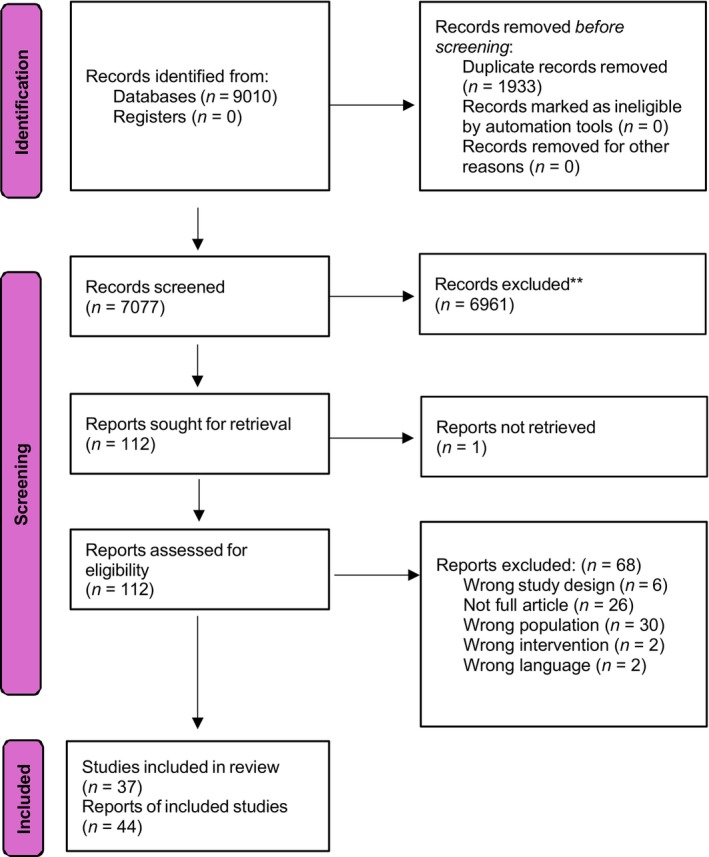
PRISMA study flow diagram.

A total of 7077 abstracts were screened, 116 full‐text articles were reviewed and 37 RCTs (44 publications) included (Figure 1).

### 3.2 Study characteristics

Table 1 summarises included study characteristics (additional details in Supporting Information S1).

**TABLE 1:** Summary characteristics of included studies.

Intervention type	Source, year	Country	Participant characteristics				Interventions and comparison			Outcomes	
			Sample size	PCOS criteria	Age (years)	BMI (kg/m^2^)	Duration of intervention	Intervention	Comparison	Prespecified primary outcomes assessed (yes/no)	Trial specific primary outcomes assessed
Lifestyle versus standard care	de Loos et al., 2023^16^ de Loos et al., 2022^17^ de Loos et al., 2021^18^ Jiskoot et al., 2020^19^	Netherlands	183	PCOS (Rotterdam)	18–38	>25	1 year	Cognitive behavioural therapy (CBT), diet and exercise with and without SMS (twenty 2.5 h group meetings)	Care as usual (advice to adopt a healthy lifestyle and to lose weight by methods of their own choosing)	Yes (pregnancy rates, live birth rate)	de Loos et al., 2023: conception (within 24 months after the start of the intervention) resulting in live birth de Loos et al., 2022: prevalence of MetS, the continuous MetS severity *z*‐score (cMetS *z*‐score), metabolic parameters (homeostatic model assessment for insulin resistance [HOMA‐IR], BP, WC, fasting glucose, fasting insulin, lipids), weight loss de Loos et al., 2021: metabolic parameters (HOMA‐IR, fasting glucose, fasting insulin) Jiskoot et al., 2020: weight
	Lee and Lee, 2023^20^	South Korea	28	PCOS (Rotterdam)	18–40	>25	12 week	Lifestyle modification (facilitated by a mobile application to complete questionnaires, access educational resources and communicate with researchers for counselling)	Usual care (maintain usual lifestyle and provided with an evidence‐based leaflet)	No	Weight loss
	Moeller et al., 2019^21^	Denmark	37	PCOS (Rotterdam)		≥30	6 months	Motivational interviewing and standard advice (advised on 1200–1500 calorie deficit per day, at least 30 min exercise per day)	Standard advice (advised on 1200–1500 calorie deficit per day, at least 30 min exercise per day)	No	Weight loss
	Oberg et al., 2019^22^	Sweden	68	PCOS (Rotterdam)	18–40	≥27	4 months	Behavioural modification programme. Group meetings three times a month on weight control, personal leadership, mindfulness and information regarding physical activity and diet in addition to monthly coaching sessions to discuss suitable individual training regimens, diet changes and to ensure compliance)	Minimal intervention (general healthy lifestyle recommendations given by a research midwife and supported by a pamphlet with written advice about diet and exercise)	No	Improved menstrual regularity
	Vigorito et al., 2007^23^	Italy	90	PCOS (European Society for Human Reproduction and Embryology/American Society for Reproductive Medicine criteria)		>25	3 months	Exercise (training sessions three times per week, structured ET programme)	No exercise (general dietary and behavioural advice without a structured caloric restriction programme)	No	Not specified
Lifestyle versus lifestyle	Asemi et al., 2014^25^, ^a^ Asemi and Esmaillzadeh, 2015^26^, ^a^	Iran	54	PCOS (Rotterdam)	18–40	>25	8 weeks	DASH hypocaloric diet (calorie‐restricted [350–700 kcal deficit]; rich in fruits, vegetables, whole grains and low‐fat dairy products and to be low in saturated fats, cholesterol, refined grains and sweets)	Control hypocaloric diet (350–700 kcal deficit)	No (pregnancy rates recorded)	Asemi 2014: not specified Asemi 2015: Hs‐CRP and LDL‐cholesterol
	Azadi‐Yazdi et al., 2017^27^	Iran	60	PCOS (ultrasound with menstrual dysfunction and/or hirsutism)	20–40	25–40	3 months	DASH calorie‐restricted eating pattern (calorie‐restricted [350–500 kcal deficit]; rich in fruits, vegetables, whole grains and low‐fat dairy products and to be low in saturated fats, cholesterol, refined grains and sweets)	Control calorie‐restricted eating pattern (350–500 kcal deficit)	No (pregnancy rates recorded)	Testosterone
	Atiomo et al., 2009^30^	United Kingdom	11	PCOS (Rotterdam but with oligo or amenorrhoea)		>30	6 months	Low GI calorie deficit diet (600 kcal deficit)	Healthy eating calorie deficit diet (600 kcal deficit hypocaloric healthy eating approach)	No	No specific biological primary outcome measures
	Cincione et al., 2023^44^	Italy	160	PCOS (ESHRE/ASRM criteria)	18–45	25–50	45 days	Mixed ketogenic diet (protein intake of 1.1–1.2 g/kg/die ideal body weight, maximum carbohydrate intake 30 g, 600 kcal per day)	Mediterranean diet (approximately 55% carbohydrates, 25% fat, 20% protein, 500 kcal deficit)	No	Not specified
	Deshmukh et al., 2023^45^	United Kingdom	40	PCOS (Rotterdam)	18–45	30–45	16 weeks	Very low calorie diet (VLCD) (8 weeks 800 kcal/day then increase by 200 kcal/2 weeks until 1600 kcal/day)	Moderate energy deficit diet (8 weeks 600 kcal/day deficit from requirements then diet introduction)	No	Free androgen index (FAI)
	Foroozanfard et al., 2017^28^	Iran	60	PCOS (Rotterdam)	18–40	>25	12 weeks	Low‐calorie DASH diet (52%–55% carbohydrates, 16%–18% proteins and 30% total fats; rich in fruits, vegetables, whole grains and low‐fat dairy products and low in saturated fats, cholesterol, refined grains and sweets; 350–700 kcal deficit)	Low calorie control diet (350–700 kcal deficit)	No (pregnancy rates recorded)	Serum AMH levels
	Johnson et al., 2015^46^	Norway	61	PCOS (Rotterdam)	18–40	35–40	8 weeks	Liquid meal replacement (flavoured meal replacement shakes with main ingredients soy protein, fructose and soy fibres in addition to unlimited intake of selected vegetables) low in fibre (e.g., salads, cucumber, tomatoes, onions), 150 g root vegetables (e.g., carrots, cabbage, kohlrabi, broccoli and cauliflower) and one fruit (5–10 g fructose) per day	Crisp bread diet (whole grain carbohydrate combined with low‐fat, high‐protein products for three of four daily meals)	No	Blood lipids, glucose metabolism, BP and uric acid
	Kasim‐Karakas et al., 2009^47^	United States	33	PCOS (by having ovarian dysfunction, as evidenced by amenorrhea [no periods for >6 months]) or oligomenorrhoea (<6 periods per year) and clinical (hirsutism) or laboratory evidence for hyperandrogenaemia (total testosterone >54 ng/dL or free testosterone >9.2 pg/ mL)	18–45	25–40	2 months	Partial meal replacement: Hypocaloric diet with sugar‐free whey protein (700 kcal deficit)	Partial meal replacement: Hypocaloric diet with simple sugars (glucose plus maltose) supplements (700 kcal deficit without altering the dietary macronutrient composition of the diet)	No	Weight, fat mass, fasting glucose and insulin, plasma lipoproteins, sex steroids.
	Mehrabani et al., 2012^48^	Iran	60	PCOS (menstrual irregularity (cycle length, 21 days or 35 days), hirsutism and biochemical hyperandrogenism)	20–40	25–38	12 weeks	Conventional hypocaloric diet (CHCD) (55% carbohydrate, 15% protein and 30% fat)	Modified hypocaloric diet (MHCD) high protein, low glycaemic load (40% low‐and medium‐GL carbohydrates, 30% protein, 30% fat, limitation of high‐glycaemic foods)	No	Not specified
	Moran et al., 2003^24^ Moran et al., 2010^49^	Not mentioned	28	PCOS (diagnosis of PCOS by menstrual irregularity (cycle length, 21 or 35 days or variation between consecutive cycles of 3 days) and clinical (hirsutism/acne) and/or biochemical hyperandrogenism)		>25	16 weeks	High protein diet (40% carbohydrate, 30% protein and 30% fat; energy‐restricted diet (6000 kJ/day) was prescribed for 12 weeks, followed by a weight‐maintenance diet for the final 4 weeks)	Low protein diet (55% carbohydrate, 15% protein and 30% fat; energy‐restricted diet (6000 kJ/day) was prescribed for 12 weeks, followed by a weight‐maintenance diet for the final 4 weeks)	No (pregnancy rates recorded)	Moran 2003: weight, body composition, dietary compliance, menstrual cyclicity, ovulation, fasting and postprandial glucose and insulin, surrogate measures of insulin sensitivity (homeostasis model of assessment), and lipid profile Moran 2010: proximal (large capacitance) LAE and distal (small resistance) SAE arterial compliance, FFA, triglycerides
	Moran et al., 2006^29^	Not mentioned	43	PCOS (Rotterdam)		>25	8 months	8‐week weight loss with 6‐month weight‐maintenance carbohydrate restriction regimen (energy‐restricted diet in which 2 meals/day were replaced with commercially available meal; then for Weeks 9–32, 120 g carbohydrate/day regimen)	8‐week weight loss with 6‐month weight‐maintenance fat restriction regimen (energy‐restricted diet in which 2 meals/day were replaced with commercially available meal; then for Weeks 9–32, 50 g fat/day regimen)	No	Not specified
	Nybacka et al., 2011^50^ Nybacka et al., 2013^51^ Nybacka et al., 2017^52^	Sweden	57	PCOS (Rotterdam)	18–40	>27	4 months	Diet (600 kcal/day deficit, 55%–60% carbohydrates, 25%–30% fat (10% saturated) and 10%–15% proteins; three main meals and two or three snacks)	Exercise (moderate to vigorous exertion level, two to three times per week duration of 45–60 min each session) versus Diet and exercise (600 kcal/day deficit, 55%–60% carbohydrates, 25%–30% fat (10% saturated) and 10%–15% proteins; three main meals and two or three snacks; moderate to vigorous exertion level, two to three times per week duration of 45–60 min each session)	No	Nybacka 2013: Serum AMH levels before and after the interventions and correlations to reproductive function, body composition and endocrine and metabolic variables Nybacka 2011: Ovarian function, endocrinologic and metabolic status and body composition. Nybacka 2017: BMI
	Pandurevic et al., 2023^53^	Italy	30	PCOS (NIH criteria)	18–45	28–40	16 weeks	8 weeks very low calorie diet (VLCKD) followed by 8 weeks low calorie diet (LCD) (three steps of 600–800 kcal/day with high‐biological‐value protein preparations obtained from cow's milk, soy, eggs, green peas and cereals, <50 g daily of carbohydrates from vegetables and 10 g of olive oil per day)	Mediterranean low calorie diet (caloric intake in the control group ranged from 1200 to 1420 kcal/day, with 15%, 30% and 55% contributions from proteins, lipids and carbohydrates, respectively)	No	Body weight, body composition
	Stamets et al., 2004^54^	United States	35	PCOS (based on a history of chronic anovulation (≤six spontaneous menstrual cycles per year) and unexplained elevated circulating T levels)	21–37	≥25	1 month	High protein diet (30% protein, 40% carbohydrate and 30% fat)	High carbohydrate diet (15% protein, 55% carbohydrate and 30% fat)	No	Change in body weight
	Vosnakis et al., 2012^55^		76	PCOS (Rotterdam)	>18	>27	7 months	7 months hypocaloric diet, physical exercise plus sibutramine 10 mg daily (hypocaloric diet, physical exercise plus sibutramine 10 mg daily for the first month and then on a hypocaloric diet plus sibutramine for the subsequent 6 months). Moderate physical activity (3 h per week)	1 month hypocaloric diet, physical exercise plus sibutramine 10 mg daily then 6 months hypocaloric diet plus exercise (hypocaloric diet, physical exercise plus sibutramine 10 mg daily for the first month and then on a hypocaloric diet and exercise for the subsequent 6 months). Moderate physical activity (3 h per week)	No	Serum AMH
	Veena Kirthika et al., 2019^56^		24	PCOS (Rotterdam)	18–25	25–29	24 weeks	PRE + aerobic exercises + diet (60 min PRE exercise session 2 days per week on consecutive days plus moderate‐intensity aerobic exercises in the form of brisk walking in a tread mill for 30 min in a day for 5 days in a week plus dietary advice from a nutritionist)	Aerobic exercises + diet (moderate‐intensity aerobic exercises in the form of brisk walking for 30 min a day for 5 days in a week plus dietary advice from a nutritionist)	No	Not specified
Pharmacotherapy versus lifestyle	Esfahanian et al., 2013^31^	Not mentioned	40	PCOS (Rotterdam)	20–30	≥27	12 weeks	Metformin 1 g/day gradually built up to 2 g/day	Hypocaloric diet (dietitian referral for 5%–10% weight reduction)	No	Not specified
	Hoeger et al., 2004^32^	Not mentioned	38	PCOS (fewer than six menses per year and evidence of hyperandrogenism)		>25	48 weeks	Metformin 850 mg twice daily	Lifestyle modification plus metformin (metformin 850 mg twice daily and meal plan with 500–1000 calorie deficit, 150 min of exercise per week) versus Lifestyle plus placebo (meal plan with 500–1000 calorie deficit, 150 min of exercise per week) versus Placebo	No	Recruitment, dropout and compliance with a long‐term lifestyle intervention in PCOS; preliminary estimates of treatment effect on ovulation, as measured by weekly urinary pregnanediol glucuronide, and on total T and free androgen index
Pharmacotherapy and lifestyle versus lifestyle alone	Elkind‐Hirsch et al., 2022^34^	Not mentioned	82	PCOS (irregular periods [cycle length outside 21–35 days] or 50 ng/dL, or free androgen index [FAI] >3.87)	18–45	≥30	32 weeks	Liraglutide 3 mg with lifestyle intervention (500–800 kcal/day; 50% carbohydrates, 20% proteins and 30% of fat; increased consumption of fibre, whole grains, cereals, fruits and vegetables; at least 30 min of moderate‐intensity physical activity daily)	Placebo with lifestyle intervention (500–800 kcal/day; 50% carbohydrates, 20% proteins and 30% of fat; increased consumption of fibre, whole grains, cereals, fruits and vegetables; at least 30 min of moderate‐intensity physical activity daily)	No	Changes in BW and FAI
	Florakis et al., 2008^57^		84	PCOS (hyperandrogenaemia [free androgen index, FAI45]) with a history of oligomenorrhoea (cycle length of 21 or 435 days; 8 cycles per year)	>18	>27	7 months	4 weeks sibutramine 10 mg daily with a 600 kcal deficient diet followed by 6 months sibutramine 10 mg daily with hypocaloric diet	4 weeks sibutramine 10 mg daily with a 600 kcal deficient diet followed by 6‐month hypocaloric diet	No	Not specified
	Lindholm et al., 2008^33^	Sweden	42	PCOS (Rotterdam)	18–40	>27	24 weeks	Sibutramine 15 mg daily with lifestyle advice (three regular meals every day, reduce fat intake, encourage a minimum of 10 000 steps a day for most days of the week.)	Placebo with lifestyle advice (three regular meals every day, reduce fat intake, encourage a minimum of 10 000 steps a day for most days of the week.)	No (pregnancy rates recorded)	Weight loss
	Moini et al., 2015^58^	Iran	100	PCOS (Rotterdam)	19–38	>25	3 months	Orlistat 120 mg three times per day and hypocaloric diet (55% carbohydrates, 30% fat and 15% protein; 1200–1800 kcal/day; normal physical activity and were encouraged to walk for 30 min daily)	Hypocaloric diet (55% carbohydrates, 30% fat and 15% protein; 1200–1800 kcal/day; normal physical activity and were encouraged to walk for 30 min daily)	No	Not specified
	Munir et al., 2018^35^	Pakistan	45	PCOS (Rotterdam)	18–40	≥30	12 weeks	Orlistat 120 BD + low caloric diet exercise (hypocaloric balanced diet of 1400 kcal/day with 5%–10% fats). 30 min/day of moderate‐intensity aerobic exercise (e.g., brisk walk, cycling or tread meal)	Low caloric diet and exercise (hypocaloric balanced diet of 1400 kcal/day with 5%–10% fats). 30 min/day of moderate‐intensity aerobic exercise (e.g., brisk walk, cycling or tread meal)	No	Occurrence of ovulation, fall in BMI
Pharmacotherapy versus pharmacotherapy	Bruno et al., 2007^59^	Not mentioned	56	PCOS (Rotterdam)		>27	6 months	Metformin (2.5 g/day)	Metformin 1.5 g/day)	No	BMI, waist circumference
	Harborne et al., 2005^36^	United Kingdom	83	PCOS		≥30	8 months	Metformin 2550 mg/day	Metformin 1500 mg/day	No	Weight loss
	Ghandi et al., 2011^37^	Not mentioned	40	PCOS (Rotterdam)	18–40	≥30	3 months	Metformin 500 mg daily uptitrated by 500 mg weekly to 500 mg three times daily	Orlistat 120 mg three times daily	No	Ovulation
	Jensterle et al., 2015^38^	Not mentioned	32	PCOS (National Institute of Child Health and Human Development (NICHD) criteria)	>18	≥30	12 weeks	Liraglutide 0.6 uptitrated to 1.2 mg daily after 1 week	Metformin 500 mg daily uptitrated by 500 mg every 3 days up to 1000 mg twice daily	No	Body weight
	Gan et al., 2023^60^	China	29	PCOS (Rotterdam)	18–40	≥25	12 weeks	Exenatide 2 mg weekly with metformin 500 mg three times daily	Metformin 500 mg three times daily	No	Change in body weight
	Jensterle et al., 2016^61^	Slovenia	44	PCOS (Rotterdam)	>18 premenopausal	≥30	12 weeks	Metformin 1 g BD and liraglutide 1.2 mg daily (metformin 500 mg daily uptitrated by 500 mg every 3 days until 1 g twice daily then commenced 1.2 mg liraglutide 14 days later)	Liraglutide (0.6 mg daily uptitrated to 1.2 mg daily after 1 week)	No	Alteration in the levels of obesity
	Jensterle et al., 2017^62^	Slovenia	30	PCOS (Rotterdam type A phenotype)	>18 premenopausal	≥30	12 weeks	Metformin 1 g BD and liraglutide 1.2 mg daily (metformin 500 mg daily uptitrated to 1 g twice daily, liraglutide 0.6 mg daily uptitrated to 1.2 mg daily)	Liraglutide 3 mg daily (0.6 mg daily uptitrated to 3 mg daily)	No	Change in anthropometric measures of obesity
Pharmacotherapy versus placebo	Jensterle et al., 2023^63^	Slovenia	20	PCOS (Rotterdam type A phenotype)		≥30	12 weeks	Semaglutide 1.0 mg weekly (0.25 mg weekly uptitrated every 2 weeks to 0.5–1.0 mg weekly)	Placebo	No	Gastric retention
Surgical versus pharmacotherapy and lifestyle	Samarasinghe et al., 2024^39^	United Kingdom	80	PCOS (2018 international evidence‐based guidelines for assessing and managing PCOS)	>18	≥35	52 weeks	Laparoscopic sleeve gastrectomy	Lifestyle based on the 2018 international evidence‐based guidelines with regular dietician reviews and pharmacotherapy including metformin (maximum dose 1 g twice daily; oral administration), orlistat (maximum dose 120 mg three times a day; oral administration) or a combination of the two	No	Biochemically confirmed spontaneous ovulatory events

Abbreviation: ASRM, American Society for Reproductive Medicine; BD, bis in die; BMI, body mass index; BW, body weight; ET, Exercise training; FFA, free fatty acids; FAI, free androgen index; GI, glycaemic index; Hs‐CPR, high‐sensitivity C‐reactive protein; NIH, National Institutes of Health; LAE, large artery elasticity; LDL, low‐density lipoprotein; PCOS, polycystic ovary syndrome; PRE, progressive resisted exercises; SAE, small artery elasticity; WC, waist circumference.

^a^ Listed author has had other articles on the DASH diet listed for retraction.

Trial participants per study ranged from 11 to 183 and intervention duration from 1 to 12 months, with most trials (30 of 37) evaluating ≤6 months interventions.

#### 3.2.1 Primary outcome measures

One study reported on two of the three primary outcomes (pregnancy and live birth rate).^16^ An additional five studies incidentally recorded pregnancy rates (Table 1).

#### 3.2.2 Secondary outcome measures

One study evaluated pregnancy complications including time to conception, GDM, hypertensive disorders in pregnancy, stillbirth, preterm delivery and NICU admission (Table 1).

All 37 RCTs reported on anthropometric outcomes. Thirty‐five studies reported on reproductive outcomes and 35 studies assessed metabolic outcomes.

### 3.3 Risk of bias

**FIGURE 2:**
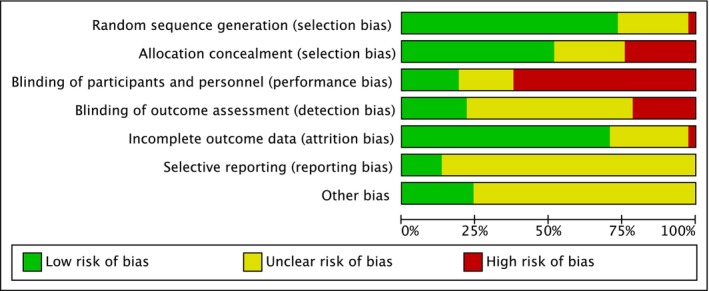
Risk of bias graph: Review authors' judgements about each risk of bias item presented as percentages across all included studies.

**FIGURE 3:**
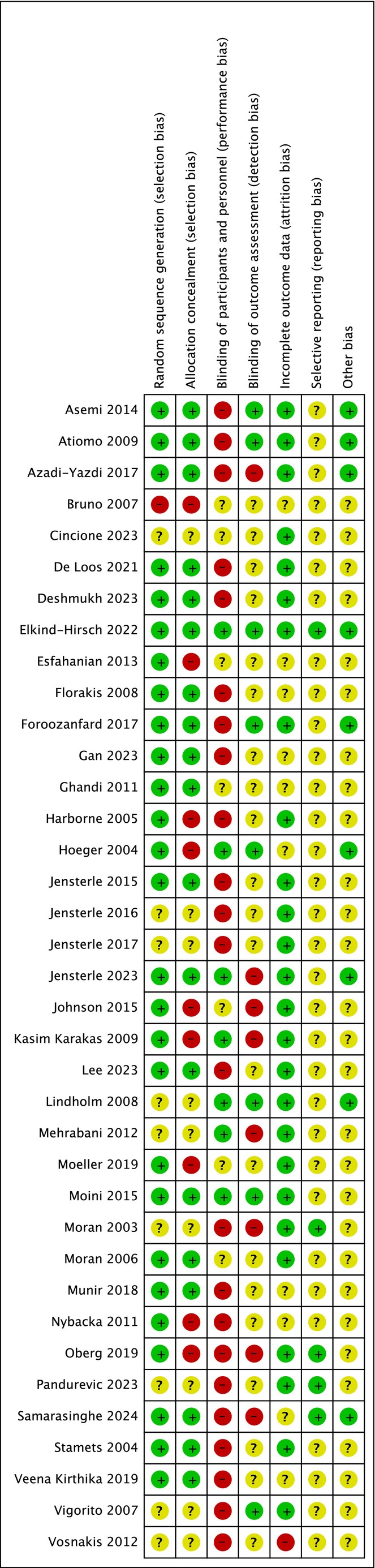
Risk of bias summary.

Risk of bias is summarised in Figures 2 and 3 (further details in Supporting Information S4). There was a low or unclear risk across most studies.

### 3.4 Results of individual studies and syntheses

Supporting Information S3 includes outcomes for included studies.

#### 3.4.1 Lifestyle intervention versus standard care

##### Pregnancy outcomes

Five studies compared lifestyle intervention to standard care, with only one of these assessing pregnancy rates as a primary outcome.^16^, ^17^, ^18^, ^19^, ^20^, ^21^, ^22^, ^23^ Rates of spontaneous pregnancy increased following 12 months of cognitive behavioural therapy (CBT), diet and exercise with/without text messaging (short message service [SMS support versus standard care (23.3% intervention with SMS, 26.7% intervention without SMS, 16.7% standard care).^16^ Weight loss was 7.87, 4.65 and 2.32 kg, respectively. However, there was no difference in live birth rate (39.8% vs. 38.3%, *p* = 0.845), mean time to conception (18.7 vs. 19.4 months, *p* = 0.646) or any antenatal outcomes when comparing this intervention with/without SMS to standard care.^16^

##### Androgenic outcomes

**FIGURE 4:**
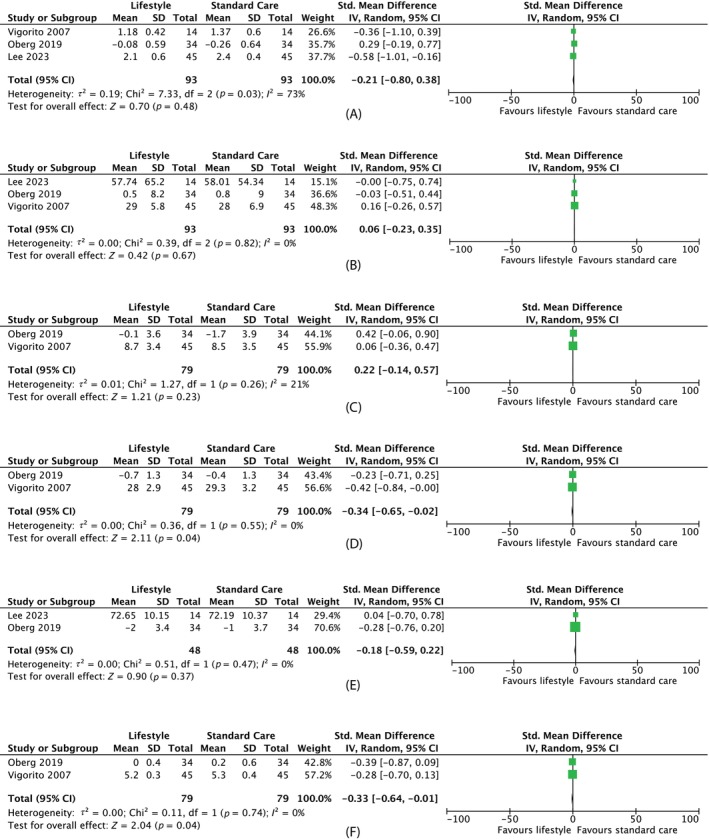
Forest plots on the effect of lifestyle intervention versus standard care. (A) Forest plot on effect of lifestyle intervention versus standard care on *testosterone* (nmol/L). Mean change from baseline was used for Oberg instead of post‐intervention data. (B) Forest plot on effect of lifestyle intervention versus standard care on sex hormone‐binding globulin (nmol/L). Mean change from baseline was used for Oberg instead of post‐intervention data. (C) Forest plot on effect of lifestyle intervention versus standard care on free androgen index (%). Mean change from baseline was used for Oberg instead of post‐intervention data. (D) Forest plot on effect of lifestyle intervention versus standard care on body mass index (kg/m^2^). Mean change from baseline was used for Oberg instead of post‐intervention data. (E) Forest plot on the effect of lifestyle intervention versus standard care on *weight* (kg). Mean change from baseline was used for Oberg instead of post‐intervention data. (F) Forest plot on effect of lifestyle intervention versus standard care on *fasting glucose* (mmol/L). Mean change from baseline was used for Oberg instead of post‐intervention data. CI, confidence interval; SD, standard deviation.

Meta‐analysis of lifestyle intervention versus standard care showed no significant change in total testosterone (MD −0.11 nmol/L, 95% CI –0.42 to 0.20, three studies, *N* = 186, *I* ^2^ = 71%), SHBG (MD 0.06 nmol/L, 95% CI −0.23 to 0.35, 3 studies, *N* = 186, *I* ^2^ = 0%) or FAI (MD 0.22, 95% CI −0.14 to 0.57, two studies, *N* = 158, *I* ^2^ = 21%) (Figure 4A–C).

Three of five studies assessed menstruation/ovulation, with only one reporting increased menstrual regularity (behavioural modification vs. standard care [MD 35% [95% CI 16–60], *p* = 0.003]) but no difference in ovulation rate.^22^ There were no significant differences in menstrual cycle after 6 months of motivational interviewing versus standard care or in ovulatory dysfunction with a 12‐month combined lifestyle programme versus standard care.^18^, ^21^

Three of five studies reported on hirsutism, with no difference between exercise versus no exercise, motivational interviewing versus standard care or lifestyle mobile app programme versus lifestyle advice.^20^, ^21^, ^23^

##### Anthropometric outcomes

Meta‐analysis of lifestyle interventions versus standard care found a significant reduction in BMI (MD −0.34 kg/m^2^, 95% CI −0.65 to −0.02, two studies, *N* = 158, *I* ^2^ = 0%) but no absolute weight loss change (MD −0.93 kg, 95% CI −2.58 to 0.72, two studies, *N* = 96, *I* ^2^ = 0%) (Figure 4D,E).

##### Metabolic outcomes

Meta‐analysis showed no difference in fasting glucose (MD −0.13 mmol/L, 95% CI −0.25 to 0.00, two studies, *N* = 158, *I* ^2^ = 0%) (Figure 4F).

#### 3.4.2 Lifestyle intervention versus other lifestyle intervention

##### Pregnancy outcomes

Out of 16 studies comparing different lifestyle interventions, four studies incidentally recorded pregnancy outcomes. Pregnancy rates were higher with a high protein versus low protein diet (8.7% vs. 4.5%), associated with greater weight loss (8.5 ± 1.1 kg vs. 6.9 ± 0.8 kg) but no difference in metabolic or androgenic profiles.^24^ Two of three studies comparing a low‐calorie DASH diet to a low‐calorie control recorded an increased pregnancy rate during the intervention (3.7% vs. 0%, 0% vs. 3.3%, 13.3% vs. 3.3%), with associated weight loss 4.4 versus 1.5 kg, 5.78 vs. 4.34 kg and 4.3 vs. 3.2 kg, respectively.^25^, ^26^, ^27^, ^28^

##### Androgenic outcomes

**FIGURE 5:**
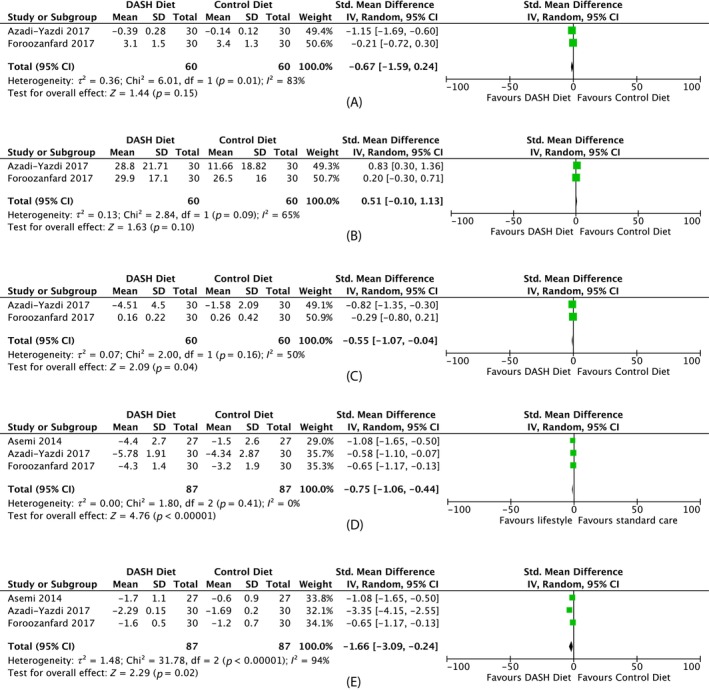
Forest plots on the effect of low‐calorie DASH diet versus low‐calorie control diet. (A) Forest plot on effect of low‐calorie DASH diet versus low‐calorie control diet on total testosterone (nmol/L). Mean change from baseline was used for Azadi‐Yazdi instead of post‐intervention data. (B) Forest plot on effect of low‐calorie DASH diet versus low‐calorie control diet on *sex hormone‐binding globulin* (nmol/L). Mean change from baseline was used for Azadi‐Yazdi instead of post‐intervention data. (C) Forest plot on the effect of low‐calorie DASH diet versus low‐calorie control diet on *free Androgen index*). Mean change from baseline was used for Azadi‐Yazdi instead of post‐intervention data. (D) Forest plot on effect of low‐calorie DASH diet versus low‐calorie control diet on *weight* (kg). Mean change from baseline was used for Asemi, Azadi‐Yazdi and Foroozanfard instead of post‐intervention data. (E) Forest plot on effect of low‐calorie DASH diet versus low‐calorie control diet on *body mass index* (kg/m^2^). Mean change from baseline was used for Asemi, Azadi‐Yazdi and Foroozanfard instead of post‐intervention data. CI, confidence interval; SD, standard deviation.

Three studies compared a low‐calorie DASH diet to a low‐calorie control diet. Meta‐analysis showed a reduction in FAI (MD −0.55, 95% CI −1.07 to −0.04, two studies, *N* = 120, *I* ^2^ = 50%) but no change in total testosterone (MD −0.67 nmol/L, 95% CI −1.59 to 0.24, 2 studies, *N* = 120, *I* ^2^ = 83%) or SHBG (MD 0.51 nmol/L, 95% CI −0.10 to 1.13, 2 studies, *N* = 120, *I* ^2^ = 65%). (Figure 5A–C). Notably, other studies by the same author evaluating the DASH diet have been listed for retraction on the Retraction Watch database. We have included these studies noting the limitations associated with this.

Five of 16 studies reported on menstrual or ovulatory changes, with only one showing improvement in ovulation from baseline with an 8‐week very low calorie diet followed by an 8‐week hypocaloric diet versus 16‐week Mediterranean diet (+46.1%, *p* = 0.031 and +21.4%, *p* > 0.05 from baseline, respectively). No other differences in menstrual regularity were seen comparing a low versus high protein diet, low glycaemic index (GI) versus hypocaloric diet or weight‐maintenance carbohydrate restriction versus fat restriction regimen.^24^, ^29^, ^30^

##### Anthropometric outcomes

Meta‐analysis comparing low‐calorie DASH diet versus low‐calorie control diet showed a reduction in weight (MD −0.75, 95% CI −1.06 to −0.44, three studies, *N* = 174, *I* ^2^ = 0%) and BMI (MD −1.66 kg/m^2^, 95% CI −3.09 to −0.24, three studies, *N* = 174, *I* ^2^ = 94%) (Figure 5D,E).

##### Metabolic outcomes

There were insufficient data to meta‐analyse.

#### 3.4.3 Pharmacotherapy versus lifestyle

##### Pregnancy outcomes

No studies reported on pregnancy outcomes.

##### Androgenic outcomes

Two studies comparing metformin to lifestyle intervention reported on menstrual function. One study found greater improvement in menstrual dysfunction with a 12‐week hypocaloric diet versus metformin 2 g daily (84% vs. 47%).^31^ The other found no difference in ovulation rate or menstrual cycle with 48 weeks of metformin 850 mg twice daily versus lifestyle versus metformin lifestyle combination versus placebo.^32^

##### Anthropometric outcomes

There were insufficient data to meta‐analyse.

##### Metabolic outcomes

**FIGURE 6:**
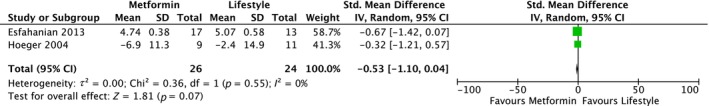
Forest plot on effect of metformin versus lifestyle intervention on *fasting glucose* (mmol/L). Mean percentage change from baseline was used for Hoeger instead of post‐intervention data. CI, confidence interval; SD, standard deviation.

Meta‐analysis on metformin compared to lifestyle showed no difference in fasting glucose (MD −0.53 mmol/L, 95% CI −1.10 to 0.04, two studies, *N* = 50, *I* ^2^ = 0%) (Figure 6).

#### 3.4.4 Pharmacotherapy with lifestyle versus lifestyle

##### Pregnancy outcomes

One study incidentally recorded pregnancy rates, with higher pregnancy rates on sibutramine and lifestyle versus placebo with lifestyle (14.2% vs. 5.0%).^33^ Sibutramine was overall associated with greater weight loss (7.8 ± 5.1 vs. 2.8 ± 6.2 kg).^33^

##### Androgenic outcomes

**FIGURE 7:**
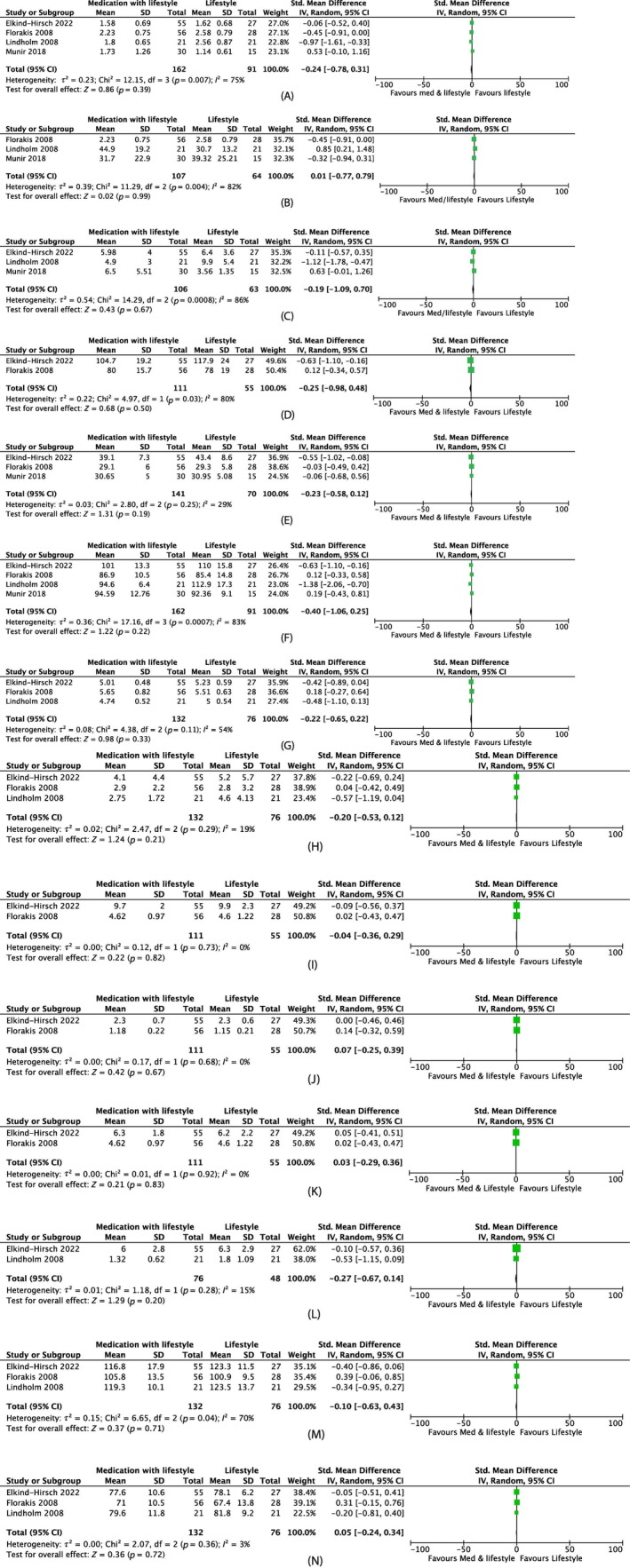
Forest plots on the effect of medication with lifestyle versus lifestyle. (A) Forest plot on the effect of medication with lifestyle versus lifestyle on total testosterone (nmol/L). (B) Forest plot on effect of medication with lifestyle versus lifestyle on s*ex hormone‐binding globulin* (nmol/L). (C) Forest plot on effect of medication with lifestyle versus lifestyle on *free androgen index*. (D) Forest plot on effect of medication with lifestyle versus lifestyle on *weight* (kg). (E) Forest plot on effect of medication with lifestyle versus lifestyle on *body mass index* (kg/m^2^). (F) Forest plot on effect of medication with lifestyle versus lifestyle on waist circumference (cm). (G) Forest plot on effect of medication with lifestyle versus lifestyle on *fasting glucose* (mmol/L). (H) Forest plot on effect of medication with lifestyle versus lifestyle on homeostatic model assessment for insulin resistance. (I) Forest plot on the effect of medication with lifestyle versus lifestyle on total cholesterol (mmol/L). (J) Forest plot on effect of medication with lifestyle versus lifestyle on high‐density lipoprotein (mmol/L). (K) Forest plot on effect of medication with lifestyle versus lifestyle on low‐density lipoprotein (mmol/L). (L) Forest plot on effect of medication with lifestyle versus lifestyle on triglycerides (mmol/L). (M) Forest plot on the effect of medication with lifestyle versus lifestyle on systolic blood pressure (mmHg). (N) Forest plot on effect of medication with lifestyle versus lifestyle on diastolic blood pressure (mmHg). CI, confidence interval; SD, standard deviation.

Meta‐analysis of pharmacotherapy with lifestyle versus lifestyle alone showed no reduction in total testosterone (MD −0.24 nmol/L, 95% CI −0.78 to 0.31, four studies, *N* = 253, *I* ^2^ = 75%), SHBG (MD 0.01 nmol/L, 95% CI −0.77 to 0.79, three studies, *N* = 171, *I* ^2^ = 82%) or FAI (MD −0.26, 95% CI −1.44 to 0.93, three studies, *N* = 169, *I* ^2^ = 91%) (Figure 7A–C), although increased statistical heterogeneity was present.

Liraglutide and lifestyle intervention resulted in almost doubling of menstrual cycles per year versus lifestyle alone (4.5 ± 0.3 to 8.65 ± 0.4 vs. 4.8 ± 0.5 to 4.8 ± 0.7, [*p* = 0.0001]).^34^ Similarly, there was a higher ovulation rate with orlistat and lifestyle versus lifestyle after 3 months follow‐up (69.02% vs. 9.09%, *p* = 0.001), although interestingly this was not associated with differences in menstrual cycle.^35^ There was no effect with sibutramine on menstrual cycles versus lifestyle.^33^

##### Anthropometric outcomes

Meta‐analysis of pharmacotherapy with lifestyle versus lifestyle showed no difference in weight (MD −0.25 kg, 95% CI –0.98 to 0.48, two studies, *N* = 166, *I* ^2^ = 80%), BMI (MD −0.23 kg/m^2^, 95% CI −0.58 to 0.12, three studies, *N* = 211, *I* ^2^ = 29%) or waist circumference (MD −0.40 cm, 95% CI −1.06 to 0.25, four studies, *N* = 253, *I* ^2^ = 83%) (Figure 7D–F).

##### Metabolic outcomes

Meta‐analysis of pharmacotherapy with lifestyle versus lifestyle showed no change in fasting glucose (MD −0.22 mmol/L, 95% CI −0.65 to 0.22, three studies, *N* = 208, *I* ^2^ = 54%), HOMA‐IR (MD −0.20, 95% CI −0.53 to 0.12, three studies, *N* = 208, *I* ^2^ = 19%), total cholesterol (MD −0.04 mmol/L, 95% CI 0.36–0.29, two studies, *N* = 166, *I* ^2^ = 0%), HDL (MD 0.07 mmol/L, 95% CI −0.25 to 0.39, two studies, *N* = 166, *I* ^2^ = 0%), LDL (MD 0.03 mmol/L, 95% CI −0.29 to 0.35, two studies, *N* = 166, *I* ^2^ = 0%) or triglycerides (MD −0.27 mmol/L, 95% CI −0.67 to 0.14, 2 studies, *N* = 124, *I* ^2^ = 15%). Similarly, there were differences in systolic BP (MD −0.10 mmHg, 95% CI −0.63 to 0.43, three studies, *N* = 208, *I* ^2^ = 70%) or diastolic BP (MD 0.05 mmHg, 95% CI −0.24 to 0.34, three studies, *N* = 108, *I* ^2^ = 3%) (Figure 7G–N).

#### 3.4.5 Pharmacotherapy versus pharmacotherapy

##### Pregnancy outcomes

No studies reported on pregnancy outcomes.

##### Androgenic outcomes

**FIGURE 8:**
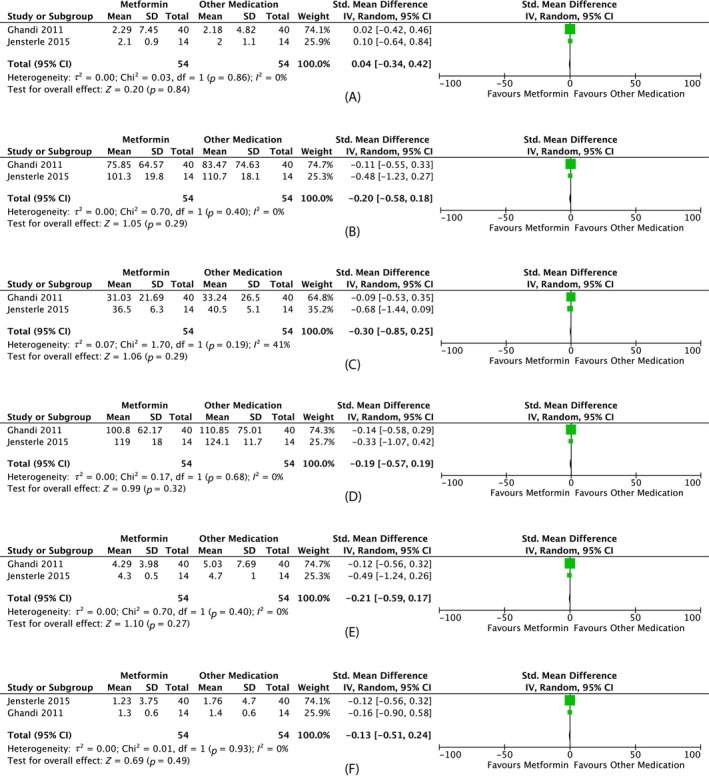
Forest plots on the effect of metformin versus other medication. (A) Forest plot on the effect of metformin versus other medication on total testosterone (nmol/L). (B) Forest plot on effect of metformin versus other medication on *weight* (kg). (C) Forest plot on effect of metformin versus other medication on *body mass index* (kg/m^2^). (D) Forest plot on the effect of metformin versus other medication on waist circumference (cm). (E) Forest plot on effect of metformin versus other medication on total cholesterol (mmol/L). (F) Forest plot on effect of metformin versus other medication on triglycerides (mg/dL). CI, confidence interval; SD, standard deviation.

Meta‐analysis of metformin versus other pharmacotherapy demonstrated no difference in total testosterone (MD 0.04 nmol/L, 95% CI −0.34 to 0.42, two studies, *N* = 108, *I* ^2^ = 0%) (Figure 8A). A single study showed a similar almost doubling of mean menstrual cycle and no difference with 1500 versus 2550 mg metformin.^36^ No ovulation or menstrual differences were seen with metformin versus orlistat^37^ or metformin versus liraglutide.^38^

##### Anthropometric outcomes

Meta‐analysis of metformin versus other pharmacotherapy showed no difference in weight (MD −0.20 kg, 95% CI −0.58 to 0.18, two studies, *N* = 108, *I* ^2^ = 0%), BMI (MD −0.30 kg/m^2^, 95% CI −0.85 to 0.25, two studies, *N* = 108, *I* ^2^ = 41%) or waist circumference (MD −0.19 kg/m^2^, 95% CI −0.57 to 0.19, two studies, *N* = 108, *I* ^2^ = 0%) (Figure 8B–D).

##### Metabolic outcomes

Meta‐analysis of metformin versus other pharmacotherapy showed no difference in total cholesterol (MD −0.21 mmol/L, 95% CI −0.59 to 0.17, two studies, *N* = 108, *I* ^2^ = 0%) or triglycerides (MD −0.13 mg/dL, 95% CI −0.51 to 0.24, two studies, *N* = 108, *I* ^2^ = 0%) (Figure 8E,F).

#### 3.4.6 Surgical versus lifestyle with pharmacotherapy

##### Pregnancy outcomes

No studies reported on pregnancy outcomes.

##### Androgenic outcomes

There were insufficient data to meta‐analyse. One study showed sleeve gastrectomy was associated with an increased ovulatory events versus lifestyle and pharmacotherapy (median 2.5 [interquartile range 1.5–4.2], *p* = 0.0007).^39^

##### Anthropometric outcomes

There were insufficient data to meta‐analyse. One study showed sleeve gastrectomy was associated with significant weight reduction (MD –29.1 kg [95% CI –37.5 to −20.8], *p* < 0.0001) and waist circumference (−17.8 cm [95% CI –24.0 to −11.6], *p* < 0.0001) versus medical care.^39^

##### Metabolic outcomes

There were insufficient data to meta‐analyse. One study demonstrated lower systolic BP (−7.9 mmHg [95% CI −13.8 to −2.0], *p* = 0.0088), diastolic BP (−9.3 mmHg [95% CI −13.9 to −4.7], *p* < 0.0001), fasting glucose (−0.5 mmol/L [95% CI −0.8 to −0.2] *p* = 0.001), fasting insulin (−12.4 mU/L [95% CI −17.6 to −7.2], *p* < 0.0001), HbA1c (−5.1 mmol/mol [95% CI −6.8 to −3.5], *p* < 0.0001), HOMA‐IR (−3.0 [95% CI −4.3 to −1.7], *p* < 0.0001) and triglycerides (−0.5 mmol/L [95% CI‐0.8 to −0.3], *p* = 0.0002) with surgical versus medical therapy.^39^

## 4 DISCUSSION

### 4.1 Main findings

This review of 37 RCTs on 2061 women assessed weight loss interventions for women with both PCOS and BMI ≥25 kg/m^2^. Meta‐analysis of lifestyle interventions versus standard care showed a BMI reduction (−0.34 kg/m^2^ [95% CI −0.65 to −0.02]), consistent with current literature.^2^, ^11^ There were insufficient data to suggest a specific lifestyle intervention, and all findings were graded as low certainty. A single RCT reporting on pregnancy rates as a primary outcome found that a 12‐month lifestyle intervention led to increased pregnancy rates (23.3%–26.7% vs. 16.7%) (low certainty) (Supporting Information S5), but no difference in live birth rate, time to conception or antenatal outcomes.^16^ Five additional studies incidentally recorded pregnancy rates during the intervention period^24^, ^25^, ^26^, ^27^, ^28^, ^33^; four showed greater pregnancy rates with intervention (3.7%–14.2% vs. 0%–5.0% for comparator), associated with 4.3–8.5 kg versus 1.5–6.9 kg weight loss, respectively.^24^, ^25^, ^26^, ^27^, ^28^, ^33^

### 4.2 Interpretation

PCOS guidelines recommend weight optimisation before pregnancy for women with BMI ≥25 kg/m^2^,^2^ but evidence for a specific approach is lacking.

Consistent with our findings, a 2019 Cochrane review of women with PCOS of any age or BMI showed lifestyle intervention (vs. minimal care) reduced BMI (MD −0.34 kg/m^2^ [−0.68 to −0.01]), weight (MD −1.68 kg [95% CI −2.66 to −0.7]) and FAI (MD −1.11% [95% CI −1.96 to −0.26]), noting this review was on women with PCOS of any age or BMI.^11^ These benefits could be attributed to behavioural changes and overall improved metabolic health and insulin resistance. A recent meta‐analysis and review on 18 RCTs comparing lifestyle to minimal treatment reported no significant difference in weight, BMI, SHBG, testosterone, FAI or fasting glucose, which was mostly in line with our findings. There was conversely an improvement in waist circumference, waist/hip ratio, hirsutism, fasting insulin, total cholesterol and LDL‐c; these studies again included women of any age and BMI and were noted to have a serious risk of bias across reported outcomes.^2^, ^41^

Our findings that metformin compared with lifestyle or pharmacotherapy interventions did not improve androgenic, anthropometric or metabolic outcomes are mostly consistent with previous data. A review comparing pharmacotherapies in women with PCOS reported greater reduction in fasting glucose only with metformin (versus Exenatide) (MD 0.10 mmol/L, 95% CI 0.02 to 0.17, *I* ^2^ = 18%, 2 RCTs).^40^ Another meta‐analysis comparing metformin to placebo for women with PCOS of any age, ethnicity or weight also demonstrated metformin benefit on fasting glucose (MD −0.13 mmol/L [95% CI −0.23 to −0.02]), as well as BMI (MD −0.89 kg/m^2^ [95% CI −1.43 to −0.35]), total cholesterol (MD −0.41 mmol/L [95% CI −0.68 to −0.14]) and LDL‐c (MD −0.35, [95% CI −0.62 to −0.08]).^2^, ^12^ However, on subgroup analysis of those with a BMI ≥25 kg/m^2^ there was a significant reduction in BMI (MD −0.89 kg/m^2^ [95% CI −1.43 to −0.35]), fasting glucose (MD −0.13 mmol/L [95% CI −0.23 to −0.02]), total cholesterol (MD −0.41 mmol/L [95% CI −0.68 to −0.14]) and LDL (MD −0.35 mmol/L, [95% CI −0.62 to −0.08]) but no difference in weight, WHR, hirsutism, FAI, SHBG, total testosterone, fasting insulin, HOMA‐IR, HDL, triglycerides or oligomenorrhoea.^12^ The contrasting limited benefit for metformin in our review may be explained by our population of interest: women of reproductive age with PCOS and BMI ≥25 kg/m^2^. Moreover, our review excluded studies which recruited women with fertility issues (i.e., anovulatory or ovulatory dysfunction or requiring fertility treatment), since this would have been a significant confounding factor for antenatal outcomes.

While GLP‐1 receptor agonists are currently not recommended immediately pre‐conception/pregnancy due to teratogenicity risks in animal studies,^42^ recent observational data in women with incidental pregnancies on these agents is reassuring.^43^

PCOS guidelines additionally suggest consideration of bariatric surgery.^2^ Our review identified one study showing significant weight loss with bariatric surgery (28.8% vs. 2.5%), associated with an increase in ovulatory events over 1 year post‐surgery. Notably, it was also associated with increased folic acid deficiency (19% vs. 5%), which is significant given the increased risk for neural tube defects.^39^ Of note, current bariatric surgery guidelines recommend avoiding pregnancy for at least 12 months post‐operatively until weight is stable.^41^

### 4.3 Strengths, limitations

Strengths of this review include it is the largest and most comprehensive evaluation of weight loss interventions in this population to date, with comparison between and within different interventions. We assessed a large number of clinically relevant outcomes and biochemical markers to provide mechanistic insights into the impact of weight loss interventions in this high risk PCOS subgroup.

Limitations included heterogeneity of interventions and outcome measures, such as inconsistent definitions, low use of core outcome sets and insufficient studies to conduct meta‐analyses across interventions. Short follow‐up times would additionally impact the ability to assess pregnancy‐related outcomes. Most included studies were of low quality, primarily due to high or unclear risk of bias, and the incidental reporting of pregnancies raises concerns of selective reporting. Most meta‐analyses relied on few studies with small sample sizes and high *I* ^2^ values. The frequently observed null results may potentially be related to lack of statistical power or type II error. The use of pairwise meta‐analysis in the context of a complex intervention network could lead to underestimation of standard errors and overstatement of precision.

### 4.4 Summary

For women with PCOS and BMI ≥25 kg/m^2^, there has only been one RCT reporting on pregnancy rates (23.3–26.7% with a 12‐month lifestyle intervention versus 16.7% with standard care) (low certainty). Meta‐analysis on lifestyle interventions compared to standard care reported a lower BMI by −0.34 kg/m^2^ ([−0.65 to −0.02]). Overall, there was modest clinical impact on metabolic, androgenic and anthropometric measures. Current recommendations for metformin and other pharmacotherapies may have less benefit for outcomes than lifestyle weight loss interventions.

Given only one study reported on the primary outcomes, this impacts the ability to assess our primary research question and highlights a significant research gap. For future studies, it would be important to have standardised interventions, outcome measures and longer follow‐up times particularly to assess pregnancy related outcomes.

## CONFLICT OF INTEREST STATEMENT

Amelia Fernandes received a postgraduate research scholarship stipend from the National Health and Medical Research Council. All other authors have no competing interests to declare.

## PEER REVIEW

The peer review history for this article is available at https://www.webofscience.com/api/gateway/wos/peer‐review/10.1111/dom.70116.

## Supporting information


**Data S1.** Supporting Information.

**Data S2.** Supporting Information.

**Data S3.** Supporting Information.

**Data S4.** Supporting Information.

**Data S5.** Supporting Information.

**Data S6.** Supporting Information.

**Data S7.** Supporting Information

## Data Availability

The data that supports the findings of this study are available in the supplementary material of this article.
