# Supplementary material for: Pre‐conception weight loss interventions in women with polycystic ovary syndrome and the effect on perinatal outcomes: A quantitative synthesis of surrogate outcomes
Source: Diabetes Obes Metab. 2025 Oct 1;27(12):7158–79. doi: 10.1111/dom.70116 (PMC12587233; doi:10.1111/dom.70116)
Supplement: Supplementary file 1 — Data S1. Supporting Information. [file DOM-27-7158-s007.docx]

**Supplementary Material 1**

***Table 2:*** *Inclusion and Exclusion Criteria*

| Search Strategy | Inclusion and Exclusion Criteria |
| --- | --- |
| Participant criteria | For a study to be included, the patient population required were woman of reproductive age with a body mass index (BMI) ≥ 25 kg/m^2^. PCOS diagnosis was based on the trialist’s definition.  We excluded studies on studies performed on women under 18 years age, who were currently pregnant, had a history of infertility or women seeking or undergoing fertility treatment or recruited from fertility clinics. Furthermore, subgroups of women with PCOS who were classified as metformin or clomiphene citrate resistant or required additional criteria such as depressive symptoms, hyperinsulinaemia or a sedentary or inactive lifestyle were not included. Participants were not excluded based on co-morbidities including type 2 diabetes or medication use, provided this medication did not affect either intervention role. |
| Intervention criteria | We included any pre-conception weight loss intervention compared to one another or to minimal intervention. Pre-conception weight loss intervention was defined as any structured lifestyle or non-pharmacological, pharmacological or surgical therapy before pregnancy to induce weight loss. These included:   - Non-pharmacological   - Lifestyle modification including education, motivational interviewing   - Behavioural or psychological therapy including behavioural therapy, cognitive behavioural therapy (CBT)   - Diet interventions including ketogenic, Mediterranean, therapeutic lifestyle changes (TLC), Pulse based, High onion, Fasting mimicking diet   - Exercise interventions including High Intensity Interval Training (HIIT), aerobic, anaerobic, strength training, yoga - Pharmacological   - Intestinal lipase inhibitors including Orlistat   - Noradrenaline and serotonin reuptake inhibitors including Sibutramine   - Glucagon Like Peptide 1 (GLP-1) receptor agonists including Exenatide, Liraglutide, Semaglutide, Dulaglutide   - Insulin sensitising medications including Metformin - Surgical   - Bariatric surgery including Gastric band, Sleeve gastrectomy, Roux-en-Y gastric bypass, Jejunoileal bypass   We excluded any interventions continued during pregnancy or fertility treatments such as hormonal therapy, the contraceptive pill, clomiphene citrate, letrozole or ovarian drilling. We did not include acupuncture, electroacupuncture, laser acupuncture or supplemental therapy such as fish oil, omega 3, Vitamin D, Vitamin E, Coenzyme Q10, ayurvedic or herbal therapy, melatonin, synbiotics or probiotics. Furthermore, interventions using anti-androgens, Myoinositol, Flutamide, Dipeptidyl-peptidase 4 (DPP4) inhibitors, Sodium-glucose Cotransporter-2 (SGLT2) Inhibitors or Phosphodiesterase-4 (PDE4) inhibitors were not included. |
| Study Criteria | RCTs were eligible for inclusion in the review including   - Any prospective interventions compared to minimal intervention or placebo - Any prospective interventions compared to another intervention   This included combination therapy or comparison of differing doses. We excluded animal studies, letters, conference abstracts, editorials, case reports. The search was limited to English language papers. |
| Outcome Measures | Primary outcomes   - Pregnancy - Live birth rate - Miscarriage   Secondary outcomes   - Perinatal   - Time to conception   - Gestational diabetes mellitus   - Pre-eclampsia   - Hypertension in pregnancy   - Stillbirth   - Preterm delivery   - NICU admission - Secondary reproductive (change after the intervention compared to baseline)   - Menstrual regularity (an initiation of menses or significant shortening of cycle length where possible), ovulation (number of ovulatory menstrual cycles where possible)   - Biochemical hyperandrogenism (total testosterone, sex hormone-binding globulin (SHBG), free androgen index (FAI)   - Clinical hyperandrogenism (hirsutism assessed clinically by Ferriman- Gallwey score) - Anthropometric (change after the intervention compared to baseline)   - Weight, BMI   - Adiposity distribution (by measures including waist circumference, waist-to-hip ratio (WHR)) - Metabolic (change after the intervention compared to baseline)   - Fasting glucose   - HbA1c   - Fasting insulin   - HOMA-IR   - Fasting lipids (total cholesterol, high-density lipoprotein cholesterol (HDL-C), low-density lipoprotein cholesterol (LDL-C), triglycerides)   - Blood pressure (systolic, diastolic) |
