# Supplementary material for: Pre‐conception weight loss interventions in women with polycystic ovary syndrome and the effect on perinatal outcomes: A quantitative synthesis of surrogate outcomes
Source: Diabetes Obes Metab. 2025 Oct 1;27(12):7158–79. doi: 10.1111/dom.70116 (PMC12587233; doi:10.1111/dom.70116)
Supplement: Supplementary file 2 — Data S2. Supporting Information. [file DOM-27-7158-s005.docx]

**Supplementary Material 2**

*MeSH terms for database search*

An independent reviewer used the following combinations of MeSH-terms (“Polycystic Ovar*”) OR (“Polycystic Ovary Syndrome”) OR (“PCOS”) AND (“Weight loss”) OR (“Weight Reduction Programs”) OR (“Weight reduc*”) OR (“Diet*”) OR (“Diet reducing”) OR (“Diet therapy”) OR (“Exercis*”) OR (“Exercise therapy”) OR (“Sports”) OR (“Life?style”) OR (“*Lifestyle Modification*”) OR (“*Lifestyle Change*”) OR (“*Behavio?r modification*”) OR (“*Behavio?r therapy*”) OR (“Cognitive Behavio?ral therapy”) OR (“*Psychotherapy*”) OR (“Bariatric surger*”) OR (“Gastric bypass”) OR (“Gastric band”) OR (“Roux-en-Y”) OR (“Gastroplasty”) OR (“Jejunoileal bypass”) OR (“Antiobesity agent*”) OR (“Orlistat”) OR (“Sibutramine”) OR (“Semaglutide”) OR (“Exenatide”) OR (“Liraglutide”) OR (“Dulaglutide”) OR (“Glucagon-Like Peptide 1*”) OR (“Appetite depressant*”) OR (“Metformin”). For Embase and Medline (Ovid), the additional keywords were used (“Randomi?ed control* trial”) OR (“Controlled clinical trial”) OR (“Clinical trial”) OR (“Trial”) OR (“Randomi?ed”) OR (“Random allocation”) OR (“Random* alloc*”) OR (“Placebo”) OR (“Control”) OR (“Single?Blind”) OR (“Single-blind Method”) OR (“Double?Blind”) OR (“Double-blind Method”).
