# Supplementary material for: Pre‐conception weight loss interventions in women with polycystic ovary syndrome and the effect on perinatal outcomes: A quantitative synthesis of surrogate outcomes
Source: Diabetes Obes Metab. 2025 Oct 1;27(12):7158–79. doi: 10.1111/dom.70116 (PMC12587233; doi:10.1111/dom.70116)
Supplement: Supplementary file 3 — Data S3. Supporting Information. [file DOM-27-7158-s006.docx]

**Supplementary Material 3**

***Table 3:*** *Characteristics of Included Studies*

| Study | Characteristics | Details |
| --- | --- | --- |
| Asemi 2014 (25)  Asemi 2015 (26) | Methods | **Study Design**  two-arm parallel, randomized controlled clinical trial (RCT)  **Duration**  8 week  **Location**  Kashan, Iran |
|  | Participants | **No of participants**  54  **Inclusion Criteria**  Quote: “Overweight or obese (body mass index [BMI] 25 kg/m2 ) women aged 18 to 40 y diagnosed with PCOS based on the Rotterdam criteria”  **PCOS Criteria**  Quote: “two of the following criteria: oligovulation, anovulation, or a combination of both; excess androgen activity (clinical or biochemical); and polycystic ovaries (by gynecologic ultrasound).”  **Exclusion Criteria**  Quote: “Ages <18 or >40 y; those with BMI < 25 kg/m2 ; women with neoplastic, hepatic, renal, cardiovascular or malabsorptive disorders; those with current or previous (within the last 6 mo) use of hormonal, antidiabetic, or antiobesity medications; and those intending to adopt a diet and/or a specific physical activity program.”  **Pre-conception intervention**  No |
|  | Interventions | **8 weeks calorie-restricted DASH eating pattern (n=24)**  Quote: “DASH diet that consisted of 52% carbohydrates, 18% proteins, and 30% total fats. The DASH diet was designed to be rich in fruits, vegetables, whole grains, and low-fat dairy products and to be low in saturated fats, cholesterol, refined grains, and sweets. Prescribed sodium in the DASH diet was <2400mg/d”; “both diets were designed to be calorie-restricted (350–700 kcal less than the computed energy requirement for each participant; 350 kcal for women with BMI 25–27.5 kg/m2 ; 500 kcal for those with BMI 27.5–31 kg/m2 ; and 700 kcal for those with BMI >31 kg/m2 ) to avoid ethical problems.”  **vs**  **8 weeks calorie-restricted control diet (n=24)**  Quote: “the control diet, also contained 52% carbohydrates, 18% protein, and 30% total fat; however, the two diets were different in terms of food groups contained”; “both diets were designed to be calorie-restricted (350–700 kcal less than the computed energy requirement for each participant; 350 kcal for women with BMI 25–27.5 kg/m2 ; 500 kcal for those with BMI 27.5–31 kg/m2 ; and 700 kcal for those with BMI >31 kg/m2 ) to avoid ethical problems.”  **Compliance**  Quote: “To facilitate compliance with the diets, participants were given and instructed to use an exchange list. To control for the participants’ dietary intakes throughout the study, the dietitian called the participants to resolve any problems. Furthermore, to examine compliance with the diets, we asked participants to record their dietary intakes every 2 wk. All participants spent about 45 min with a dietitian learning the basics of their diets.” |
|  | Outcomes | Weight, BMI, waist circumference, hip circumference  Total chol, triglycerides, VLDL, HDL, LDL, TC:HDL ratio, fasting glucose, insulin, HOMA-IR, HOMA-B, QUICKI, CRP  Plasma total antioxidant capacity (TAC) and total glutathione (GSH) |
| Atiomo 2009 (30) | Methods | **Study Design**  Pilot randomized controlled trial (RCT)  **Duration**  6 month  **Location**  Nottingham, UK |
|  | Participants | **No of participants**  11  **Inclusion Criteria** Quote: “polycystic ovary syndrome using the Rotterdam criteria (but with oligo or amenorrhoea) and a body mass index (BMI) above 30”  **PCOS Criteria**  Quote: “using the Rotterdam criteria”  **Exclusion Criteria**  Quote: “previous or current history of any cancer, use of the combined pill, progestogens or clomiphene, women about to undergo intrauterine insemination and in-vitro fertilisation”  **Pre-conception intervention**  No |
|  | Interventions | **6 month 600 calorie deficit low glycaemic index diet (n=6)** Quote: **“**600 kcal deficit low glycaemic diet”  **vs**  **6 month 600 calorie deficit healthy eating diet (n=5)** “Quote: “600 kcal deficit hypocaloric healthy eating approach using a web-based randomisation pro- gramme designed for the study. They were then provided with a menu of ideal meal plans and educated on food types to conform to the groups they had been assigned to. “  **Compliance** Quote: **“**compliance was measured based on their attendance and from completing a food diary ” |
|  | Outcomes | Weight, Waist circumference, Hip circumference, BMI  Systolic BP, Diastolic BP, Glucose, Total cholesterol, HDL, LDL, Triglycerides, HDL/LDL cholesterol ratio, Insulin  Estradiol, LH, SHBG, Testosterone, Change in number of periods in the last 6 months  Endometrial thickness on ultrasound  Eligibility and recruitment rates, compliance with the allocated diet for 6 months and with clinical assessments |
| Azadi-Yazdi 2017 (27) | Methods | **Study Design**  Parallel randomised controlled clinical trial  **Duration**  12 weeks  **Location**  Follow-up carried out in Yazd, Iran |
|  | Participants | **No of participants**  60  **Inclusion Criteria**  Quote: “Overweight or obese (BMI = 25–40 kg m^2^ ) women aged 20–40 years who were newly diagnosed with PCOS based on the Rotterdam criteria, did not use hormonal contraception or other medications that could alter the concentration of androgens, did not use hormones as medication for 3 months prior to the study, were without type 1 diabetes, and were not using anti-obesity medications or engaging in a specific physical activity program”  **PCOS Criteria**  Quote: “PCOS was identified by ultrasonography (12 or more small follicles observed in an ovary on ultrasound examination) in participants with menstrual dysfunction (the presence of chronic amenorrhea or a menstrual cycle length of less than 21 days or more than 35 days, or more than 4 days of variation between cycles) and/or hirsutism.”  **Exclusion Criteria**  Quote: “hormonal therapy or other medications that could affect PCOS or weight were initiated for them during the study”  **Pre-conception intervention**  No |
|  | Interventions | **Calorie-restricted DASH eating pattern (n=30)**  Quote: **“**Rich in fruits, vegetables, whole grains and low-fat dairy products, as well as low in saturated fats, cholesterol, refined grains and sweets. The amount of sodium intake was also designed to be less than 2400 mg day.**”** “50–55% carbohydrate, 15–20% protein and 25–30% total fat” diet. **“**The energy requirements of each participant were estimated based on physical activity level and resting energy expenditure by the use of the Harris–Benedict equation (18). Both diets were designed to be energy-restricted: 1.46–2.09 MJ (350– 500 kcal) less than the computed energy requirement for each participant; 1.46 MJ (350 kcal) for subjects with BMI in the range 25–29.9 kg/m^2^ ; 2.09 MJ (500 kcal) for those with the BMI in the range 30–39.9 kg/m^2^.”  **vs**  **Calorie-restricted control diet (n=30)**  Quote: “50–55% carbohydrate, 15–20% protein and 25–30% total fat” diet. **“**The energy requirements of each participant were estimated based on physical activity level and resting energy expenditure by the use of the Harris–Benedict equation (18). Both diets were designed to be energy-restricted: 1.46–2.09 MJ (350– 500 kcal) less than the computed energy requirement for each participant; 1.46 MJ (350 kcal) for subjects with BMI in the range 25–29.9 kg m–2 ; 2.09 MJ (500 kcal) for those with the BMI in the range 30–39.9 kg m–2 .”  **Compliance**  Quote: **“**To monitor adherence to the diet, participants were asked to record their dietary intakes for 3 days (2 week days and 1 weekend) in each month. Moreover, every month, a 24-h dietary recall was completed by dietitian and each participant received a bimonthly visit from a dietitian.” “The compliance of participants was maximised by frequent visits to the dietitian and the provision of detailed food plans with menu. Although controlled feeding is a gold standard study design, studies conducted in free-living populations are more reflective of nutritional management in routine clinical practice.” |
|  | Outcomes | Testosterone  Androstenedione  SHBG  Height, weight, waist and hip circumference  Body composition  Total antioxidant capacity |
| Bruno 2007 (59) | Methods | **Study Design**  prospective, randomized study  **Duration**  6 months  **Location**  Not mentioned |
|  | Participants | **No of participants**  56  **Inclusion Criteria**  BMI 27 kg/m2, PCOS  **PCOS Criteria**  Rotterdam criteria  Quote: “based on free androgen index (100 [T/sex hormone– binding globulin (SHBG)]) greater than 8; menstrual irregularities (oligo-/amenorrhea); Ferriman-Gallwey scale greater than 8; and the presence of polycystic ovaries”  **Exclusion Criteria**  Not mentioned  **Pre-conception intervention**  No |
|  | Interventions | **6 months of Metformin (2.5 g/day)**  **vs**  **6 months of Metformin 1.5 g/day)** |
|  | Outcomes | BMI Waist circumference |
| Cincione 2023 (44) | Methods | **Study Design**  Randomised controlled trial  **Duration**  45 days  **Location**  Foggia, Italy |
|  | Participants | **No of participants**  160  **Inclusion Criteria**  Quote: “The inclusion criteria for all groups were: premenopausal women who were over- weight or obese (until BMI 49.9 kg/m2), aged 18–45 years (fertile age), PCOS, a lack of underlying metabolic disease (type 2 diabetes, hypertension, diagnosed anaemia, or any other metabolic disease requiring a special diet).”  **PCOS Criteria**  Quote: “classified by the European Society for Human Reproduction and Embryology/American Society for Reproductive Medicine (ESHRE/ASRM) diagnosis. This includes the presence of two of the three features of hyper-androgenism (either clinical (hirsutism by elevated Ferriman–Gallwey score)) or biochemical (elevated tes- tosterone or free androgen index), oligomenorrhea (inter- val between two menstrual periods more than 35 days), or amenorrhea (no vaginal bleeding for at least six months) and the presence of polycystic ovaries on ultrasound scan (≥ 12 follicles measuring 2–9 mm in diameter, or ovarian volume > 10 mL in at least one ovary).”  **Exclusion Criteria**  Quote: “Age < 18 years and > 45 years, Menopause (defined as amenorrhea for ≥ 3 years or amenorrhea for ≥ 1 but < 3 years and plasma follicle- stimulating hormone concentrations elevated to the post- menopausal range), Pregnancy or lactation in the past 6 months, Kidney, liver or heart disease, Gout or hyperuricemia, Hyperandrogenism and/or biochemical hyper-androgenemia, oligomenorrhea due to secondary etiologies as per the Endocrine Society Clinical Practice Guidelines and previous publications including endocrine disorders (congenital adrenal hyperplasia, androgen-secreting tumors, Cushing’s syndrome, hyperprolactinaemia, thy- roid dysfunction and adrenal disorders), Pre-existing systemic or psychiatric disease, Use of medications that impact carbohydrate metabolism (oral contraceptive pills, metformin, anti-epileptics, anti-psychotics and glucocorticoids), Specific nutritional regimens or hypocaloric diet in the last three months, Occasional or current of use of drugs that could influence fluid balance, including non-steroidal anti-inflammatory drugs, diuretics, laxative use. ”  **Pre-conception intervention**  No |
|  | Interventions | **45 day Mixed ketogenic diet (n=73)**  Quote: **“**The “mixed ketogenic” diet included a daily protein intake, in part, isolated whey protein powder derived from milk with a high biological value and complete amino acid com- position profile with near-zero carbohydrates and fat con- tent, and partly from animal protein sources, such as meat, fish, or eggs. The whey protein powders adopted are iso- late milk whey protein enriched with free essential acids and plant protease (Aminexem, Named, Italy). The average nutritional values for two bags of isolate milk whey protein powder enriched with a specific mixture of essential free amino acids contained protein powder 31 gr, carbohydrates 1,2 gr, fats 0,4 gr, essential free amino acid 17,8 g, equiva- lent to a value of 57% of EAA, where the highest content in EAA found in nature has an EAA of 46–47%, isoleu- cine 2,69 gr, phenylalanine 0,69 gr, leucine 4,08 gr, lysine 3,58 gr, methionine 0.50 gr, threonine 1,52 gr, tryptophan 0,32 gr, histidine 1,81 gr, leucine isoleucine 4,08 gr, iso- leucine 2,69 gr and valine isoleucine 2,58 gr, vitamin D 50, thiamine 1,12 mg, riboflavin 1,4 mg, vitamin B6 1,4 mg, and plant protease, papain 250 mg with enzymatic activ- ity 50.000 TU, tyrosine unit/mg/min, bromelain 250 with enzymatic activity 250 GDU, gelatin digestion unit, for a total of 133 kcal for two bags. A protein intake of 1.1–1.2 g/ kg/die ideal body weight was used. The maximum allowable intake of daily carbohydrates was set at 30 g, the total daily caloric intake was established at around 600 kcal; thus, the diet was a very-low-calorie ketogenic diet, this induced a drastic reduction of calories aimed at obtaining weight loss mainly from the FM. The lipid component was set to 30 g/ day, mainly consumed in the form of extra virgin olive oil in the amount of 10 g taken during the evening meal, to which the lipid quotas contained in meat and fish and in oil-dried nuts and oilseeds were added. It is essential to highlight that the KD is not balanced from the point of micronutrients, and for this reason, multivitamin and multi-mineral supplements were administered throughout the mixed KD period to avoid nutritional deficiencies.”  **Vs 45 day Mediterranean diet (n=71)**  Quote: “The MD diet was rich in whole grains (pasta, bread, and whole wheat), eggs, poultry, fish, vegetables, legumes, fruits and olive oil as the main condiment, and low in red and processed meat, according to the Mediterranean-style diet pyramid. The initial assigned energy intake of the MD diet was determined based on the individual’s habitual energy intake evaluated by a nutritional visit by qualified nutritionist during a face-to-face interview, adjusted for body weight and clinical judgment of the nutritionists, to take care of a possible underreporting, common in overweight/obese individuals. The energy distribution of macronutrients was composed of approximately 55% carbohydrates (especially whole wheat), 25% fat (PUFA from olive oil, almonds and pistachios) and 20% protein (especially fish and legumes). The caloric intake was 500 kcal less than the individual daily caloric requirement, and dietary profiles were calculated on the basis of the portion sizes recommended by the Italian Recommended Dietary Allowances”  **Compliance** Adherence to ketogenic diet not discussed  Quote: “adherence to MD was evaluated by the nutritionist through counseling every 2 weeks and reinforced by phone calls every 2–3 d” |
|  | Outcomes | Weight, BMI, waist circumference, hip circumference, waist hip ratio, fat mass, free fat mass, basal metabolism, total body water  Glucose, insulin, HOMA-IR, C-peptide, serum albumin  LH, FSH, LH/FSH, free testosterone, total testosterone, SHBG |
| De Loos 2021 (18)  De Loos 2022 (17)  De Loos 2023 (16)  Jiskoot 2020 (19) | Methods | **Study Design**  Randomised controlled trial  **Duration**  1 year  **Location**  the Erasmus MC, the Netherlands. |
|  | Participants | **No of participants**  183  **Inclusion Criteria**  Quote: “women who were actively trying to conceive with a BMI > 25 kg/m2, between 18 and 38 years of age, and a diagnosis of PCOS”  **PCOS Criteria**  Quote: “according to the Rotterdam 2003 consensus criteria”  Quote: “at least two of the following key features were present: ovulatory dysfunction (cycle interval length > 35 or < 21 days), clinical (modified Ferriman Gallwey score ≥ 5), and/or biochemical (testosterone measured with RIA: free androgen index (FAI) cut off > 4.5 and/or total testosterone > 3.0, testosterone measured with liquid chromatography-tandem mass spectrometry (LC-MS/MS): FAI cut off > 2.9 and/or total testosterone > 2.0 nmol/L) hyperandrogenism and polycystic ovarian morphology (PCOM; ≥12 follicles (measuring 2–9 mm in diameter) and/or ovarian volume > 10 cm3 in at least one ovary using an ultrasound machine with a transvaginal probe of less than 8 MHz)”  **Exclusion Criteria**  Quote: “the lack of proficient use of the Dutch language, severe mental illness, adrenal diseases or ovarian tumours, and other causes leading to androgen excess, and other malformations of the internal genitalia. With the use of an extensive endocrine screening, which is specified below, women with other secondary endocrine (e.g. hypothyroidism, Cushing’s disease, hypothalamic obesity, hypogonadism, and insulinoma), drug-related, and, when indicated, genetic causes of secondary obesity were identified and excluded.”  Quote: “Participants did not use any medication like oral contraceptives or metformin during the study period. We excluded women who became pregnant during the study.””  **Pre-conception intervention**  Yes |
|  | Interventions | **SMS+: 1 year of CBT, diet, and exercise intervention with SMS support (n=60)**  Quote: “covered three components in twenty 2.5-h group meetings: cognitive behavioural therapy (CBT), diet, and exercise. CBT techniques were used to, for example, create awareness and to restructure dysfunctional thoughts about lifestyle, weight (loss), and self-esteem. The ‘Dutch Food Guide’ was used as a guideline for healthy eating (29), and the global recommendations for physical activity by the World Health Organization (30) formed the basis for the exercise component of the LSI. After 3 months, half of the LSI group received additional patient-tailored SMS feedback. Participants sent weekly self-monitored information regarding their diet. physical activity, and emotions by SMS. A semi-automated software programme generated feedback in response to the incoming messages with the goal to encourage positive behaviour and provide social support. Participants also received two additional messages per week addressing eating behaviour and physical activity. ”  **vs**  **SMS-: 1 year of CBT, diet, and exercise intervention (n=63)**  Quote: “covered three components in twenty 2.5-h group meetings: cognitive behavioural therapy (CBT), diet, and exercise. CBT techniques were used to, for example, create awareness and to restructure dysfunctional thoughts about lifestyle, weight (loss), and self-esteem. The ‘Dutch Food Guide’ was used as a guideline for healthy eating (29), and the global recommendations for physical activity by the World Health Organization (30) formed the basis for the exercise component of the LSI.”  **vs**  **Care as usual (CAU): 1 year of Weight-loss advice only (n=60)**  Quote: “comprised an advice to adopt a healthy lifestyle and to lose weight by methods of their own choosing (e.g. visit a dietician or gym) and consultations with their treating physician.” |
|  | Outcomes | Waist circumference  Fasting glucose, fasting insulin, Cholesterol, HDL, LDL, Triglyceride, metabolic syndrome, metabolic parameters, homeostasis model assessment (HOMA-IR), systolic BP, diastolic BP  Ovulatory dysfunction, Clinical hyperandrogenism, Biochemical hyperandrogenism  PCOM AFC, PCOM Volume  Conception resulting in live birth, stillbirth, spontaneous conception, conception with ART, conception (unknown mechanism), vaginal birth, instrument-assisted/ caesarean section, unknown delivery, gestation diabetes, hypertensive disorders in pregnancy, preterm birth, postpartum haemorrhage, APGAR score < 7 after 5 minutes, NICU admission, small for gestational age, large for gestational age, congenital abnormalities, birth weight (grams/percentile), gestational age at delivery, APGAR at 5 minutes |
| Deshmukh (45) | Methods | **Study Design**  Open-label randomised controlled trial  **Duration**  16 weeks  **Location**  Hull, United Kingdom |
|  | Participants | **No of participants**  **40**  **Inclusion Criteria**  Quote: “women wishing to lose weight, aged between 18 and 45 years, had a body mass index (BMI) between 30 and ≤45 kg/m2 (based on the dimensions of the DEXA scanner), and were diagnosed with PCOS based on the Rotterdam criteria” and “willing to use a reliable form of non-hormonal contraception throughout the duration of the study”  **PCOS Criteria**  Quote: “(biochemical hyperandrogenism, as indicated by a FAI > 4, and self-reported oligomenorrhoea (cycle length > 35 days and nine or fewer periods per year) or amenorrhoea (absence of menses for a period ≥ 3 months)”  **Exclusion Criteria**  Quote: “Women with differential diagnoses of non-classical 21-hydroxylase deficiency, hyperprolactinaemia, Cushing’s disease, and androgen-secreting tumours were excluded from participation. Additional exclusion criteria included menopause and perimenopause, pregnancy or intention to become pregnant, breastfeeding, weight loss > 5 kg within the last 6 months, substance abuse, acute illness, diagnosis of diabetes, history or presence of malignant neoplasms within the last 5 years, history of gallstones/gout, inadequately controlled thyroid disorder, diagnosis of eating disorder or purging in the last 12 months (based on patient reporting, results of Eating Disorder Inventory, 3 Referral Form (EDI-3 RF) interpreted by a clinical psychologist), known intolerance to the ingredients of investiga- tional products used in the study (e.g., soy, lactose, gluten), or coeliac disease. Participants who were using the following drugs (within the last three months) were also excluded from participation unless cessation of the drug was agreed upon between the medical team and the patient and a wash-out period of 4–8 weeks was achieved; these drugs were: oral hor- monal contraceptives and hormone-releasing implants, anti-androgen (e.g., spironolactone, flutamide, finasteride), metformin or other insulin-sensitising medications, clomiphene citrate or estrogen modulators, gonadotropin-releasing hormone (GnRH) modulators (e.g., leuprolide), Minoxidil, anti-obesity drugs, or other medication that may affect appetite (e.g., oral steroids).  **Pre-conception intervention**  No |
|  | Interventions | **16 week Very Low Calorie Diet (VLCD) (8 weeks 800kcal/day then increase by 200 kcal/2 weeks until 1600 kcal/day) (n=21)**  Quote: **“**800 kcal a day (irrespective of their baseline body weight), in the form of soups and drinks made from pre-prepared sachets provided by the Cambridge Weight PlanTM company (Corby, UK). Each meal replacement drink provided 200 kcal, 21 g CHO, 15 g of protein, 3–4 g fats, and was nutritionally complete for micronutrients as they are specifically designed to be used as a sole source of nutrition and total meal replacement diets (The Cambridge Weight Plan Ltd., (Corby, UK)). Participants in this group were provided support and information regarding the consumption of the food replacement sachets, fibre supplement prescription, and fluid consumption. After these first eight weeks, participants in the VLCD arm were given a stepped return, an increase of 200 kcal/2 weeks whilst reducing meal replacement drinks until ~1600 kcal/d was reached. Both groups received dietetic support and education on different aspects including portion sizes and kcals, practical measures to achieve the given energy prescription, and healthy eating practices based on the “Eat Well Guidelines””  **Vs 16 week moderate energy deficit (8 weeks 600kcal/day deficit from requirements then diet introduction (n=19)**  Quote: “kcal prescription was bespoke for each patient and calculated using the Henry equation based on gender, age, and weight to ascertain basal metabolic rate, which was then multiplied by physical activity level (PAL). Once the patients’ daily kcal requirements had been calculated, a deficit of 600 kcal from requirements was applied. Both groups received dietetic support and education on different aspects including portion sizes and kcals, practical measures to achieve the given energy prescription, and healthy eating practices based on the “Eat Well Guidelines””  **Compliance**  Review at two weeks after commencement of intervention including an assessment of compliance |
|  | Outcomes | FAI, total testosterone, DHEAS, Androstenedione, SHBG, LH, FSH  Fasting glucose, 2 hour glucose on OGTT, Hba1c, total cholesterol, triglycerides  Weight, BMI, waist circumference, waist hip ratio  Body composition (Total fat, trunk fat, lean body mass, fat free mass, BMD, BMC) CRP, ALT, AST |
| Elkind-Hirsch 2022 (34) | Methods | **Study Design**  Randomized, double-blind, placebo-controlled study  **Duration**  32 weeks  **Location**  Not mentioned |
|  | Participants | **No of participants**  82  **Inclusion Criteria**  Quote: “nondiabetic, premenopausal women, aged 18–45 years, diagnosed with PCOS with a body mass index of at least 30 kg/m2”  Quote: “agreement to use effective contraception consistently during the therapy”  Quote: “Eligible subjects were required to have irregular periods (cycle length outside 21–35 days or 50 ng/dL, or free androgen index [FAI] >3.87)”  **Exclusion Criteria**  Quote: “exclusion of known disorders for bleeding irregularities and androgen excess”  Quote: “diabetes diagnosis, smoking within 6 months, pregnancy or lactation, clinically significant systemic disease, uncontrolled hypertension, acute pancreatitis, injectable hormonal contraceptive use within 6 months, use of oral contraceptives, other steroid hormones, drugs that affect gastrointestinal motility or carbohydrate metabolism, and/or anti-obesity drugs within 3 months before study entry.”  **Pre-conception intervention**  No |
|  | Interventions | **32 weeks of Liraglutide 3 mg with lifestyle intervention**  Quote: “LIRA 3 mg and PL were provided by Novo Nordisk A/S in identical prefilled pens labeled with serial numbers and accompanied by a dispensing unit list. Printed directions for use were provided by Novo Nordisk A/S and were handed out to participants at the first dispensing visit.”  Quote: “all participants met with the registered dietician and received lifestyle recommendations, including individualized advice for a healthy food diet and physical activity. A diet of 500–800 kcal/day reduction made up of 50% carbohydrates, 20% proteins, and 30% of fat with increased consumption of fiber, whole grains, cereals, fruits, and vegetables along with at least 30 minutes of moderate-intensity physical activity daily was recommended to all women. During the study, lifestyle intervention was actively promoted although this was not formally assessed. The participants received further encouragement to adhere to the regime during follow-up phone calls.”  **Vs**  **32 weeks of placebo with lifestyle intervention**  Quote: “LIRA 3 mg and PL were provided by Novo Nordisk A/S in identical prefilled pens labeled with serial numbers and accompanied by a dispensing unit list. Printed directions for use were provided by Novo Nordisk A/S and were handed out to participants at the first dispensing visit.”  Quote: “all participants met with the registered dietician and received lifestyle recommendations, including individualized advice for a healthy food diet and physical activity. A diet of 500–800 kcal/day reduction made up of 50% carbohydrates, 20% proteins, and 30% of fat with increased consumption of fiber, whole grains, cereals, fruits, and vegetables along with at least 30 minutes of moderate-intensity physical activity daily was recommended to all women. During the study, lifestyle intervention was actively promoted although this was not formally assessed. The participants received further encouragement to adhere to the regime during follow-up phone calls.”  **Compliance**  During the whole study period, compliance with the treatment was documented. |
|  | Outcomes | Body weight, Waist circumference, BMI  Total testosterone, DHEAS, Menstrual frequency  Fasting blood glucose, MBG during an OGTT, surrogate measures of insulin action (HOMA-IR, SIOGTT, IGI/HOMA), Lipids, TRG/HDL-C ratio, BP  Body composition (total fat mass percentage, lean body mass, and abdominal [AGR]) |
| Esfahanian 2013 (31) | Methods | **Study Design**  Randomised controlled trial  **Duration**  12 weeks  **Location**  Not mentioned |
|  | Participants | **No of participants**  40  **Inclusion Criteria**  Quote: “aged 20–30 years, with body mass index (BMI) 27 kg/m2 .”  **PCOS Criteria**  Quote: “Criteria for PCOS were as defined by Rotterdam and at least two of the three following: oligo-ovulation or anovulation (12 weeks); clinical and/or biochemical signs of hyperandrogenism (hirsutism, acne, male alopecia and/or elevated serum total of testosterone 60 ng/dL); and polycystic ovaries. Hirsutism was defined as the presence of excess terminal hairs in androgen-dependent areas on a woman, and it was measured objectively using the modified Ferriman–Gallway (mF-G) scoring system. Hirsutism was determined as a scale of more than 7.”  **Exclusion Criteria**  Quote: “Other causes of hyperandrogenism and menstrual irregularity were excluded after careful examination and valid laboratory tests.”  Quote: “Diabetic subjects, smokers, alcohol users and those taking sex steroids or drugs known to affect lipid metabolism and weight during the 3 months preceding the study were excluded.”  Quote: “Because oral contraceptive pills affect our results and use of barrier methods was difficult for our subjects, we excluded the patient if pregnancy occurred”  **Pre-conception intervention**  No |
|  | Interventions | **12 weeks of Metformin**  Quote: “Metformin was given at a dose of 1000 mg/day in divided doses and built gradually to 2000 mg/day”  **Vs**  **12 weeks of Hypocaloric diet**  Quote: “Subjects in the diet group were referred to a dietitian for 5–10% weight reduction. Frequent contacts with participants (telephone, electronic communication and visit) were performed every week to support continued drug consumption and diet program.” |
|  | Outcomes | Weight, BMI, Waist and hip circumference  Total and free testosterone  high-sensitivity CRP (hs-CRP)  total cholesterol (Tch), high-density lipoprotein cholesterol (HDL-C), low-density lipoprotein cholesterol (LDL-C), triglycerides (TG)  fasting insulin  oral glucose tolerance test (OGTT)  homeostasis model assessment-IR (HOMA-IR), quantitative insulin-sensitivity check index (QUICKI) and fasting glucose to insulin ratio (FGIR).  Improvement of cycle disorder (defined as alteration of menstrual habit in these turns: amenorrhea, oligomenorrhea, eumenorrhea or reduction in cycle length of at least 4 weeks or pregnancy.) |
| Florakis 2008 (57) | Methods | **Study Design**  prospective, open-label, randomized, comparative trial  **Duration**  4 weeks run in period then randomised to 6 month intervention  **Location** |
|  | Participants | **No of participants**  84  **Inclusion Criteria**  **“**Outpatients, premenopausal, nonpregnant, nonlactating, overweight and obese women (body mass index, BMI427), 18 years of age and older with PCOS **”**  **PCOS Criteria**  “based on evidence of hyperandrogenemia (free androgen index, FAI45) with a history of oligomenorrhea (cycle length o21 or435 days; o8 cycles per year). ”  **Exclusion Criteria**  Women with no classical 21-hydroxylase deficiency, hyperprolactinemia, adrenal or ovarian tumor and Cushing’s disease were excluded by the appropriate tests. Other exclusion criteria were hypertension, thyroid dysfunc- tion, overt diabetes mellitus and concomitant treatment, such as antihypertensive drugs, selective serotonin reuptake inhibitor or other SNRI drug, oral contraceptive pills or any other antiandrogen treatment (cyproterone acetate, spiro- lactone, luteinizing hormone (LH) release hormone agonist) and insulin-sensitizing agents (metformin, pioglitazone, rosiglitazone) that may interact with insulin sensitivity and lipid profile.  **Pre-conception intervention** |
|  | Interventions | 4 week sibutramine 10mg daily with a 600 kcal deficient diet followed by 6 month sibutramine 10mg daily with hypocaloric diet **(n=56)** ”Before entering the run-in period all subjects were prescribed an energy-restricted diet containing 50% of energy as carbohydrate, 30% of fat (10% saturated) and 20% of protein. Subjects were advised not to modify their eating habits throughout the study period. **” “**a run-in period (4 weeks duration) that all patients received 10mg per day sibutramine plus a 600 kcal deficient diet, a treatment period (for the subsequent 6 months) that subjects were rando- mized in a 2:1 ratio to the S group (10mg per day of sibutramine plus hypocaloric diet)**” “**Diet was based on individual basal metabolic rate as defined by the Harris- Benedict equation, adjusted for moderate physical activity.”  **Vs** 4 week sibutramine 10mg daily with a 600 kcal deficient diet followed by 6 month hypocaloric diet **(n=28)** ”Before entering the run-in period all subjects were prescribed an energy-restricted diet containing 50% of energy as carbohydrate, 30% of fat (10% saturated) and 20% of protein. Subjects were advised not to modify their eating habits throughout the study period. **” “**all patients received 10mg per day sibutramine plus a 600 kcal deficient diet, a treatment period (for the subsequent 6 months) that subjects were rando- mized in a 2:1 ratio to the S group (10mg per day of sibutramine plus hypocaloric diet) and the D group (hypo- caloric diet only).**” “**Diet was based on individual basal metabolic rate as defined by the Harris- Benedict equation, adjusted for moderate physical activity.”  **Compliance**  “Monthly, subjects’ body weight was recorded, adverse events, heart rate, blood pressure and study drug compliance were determined ” |
|  | Outcomes | Body weight, BMI, waist circumference, waist hip ratio total testosterone (T), SHBG, dehydroepiandrosterone sulfate (DHEAS), androstenedione (D4A), 17a-hydroxyprogesterone, follicle-stimulating hormone (FSH), LH, thyroid-stimulating hormone, prolactin total cholesterol (TC), triglycerides (TG), low-density lipoprotein cholesterol (LDL-C), high-density lipoprotein cholesterol (HDL-C), fasting glucose, fasting insulin, HOMA-IR, systolic BP, diastolic BP, heart rate serum glutamic oxaloacetic transaminase (SGOT), serum glutamic pyruvic transaminase (SGPT) |
| Foroozanfard 2017 (28) | Methods | **Study Design**  Randomized controlled trial  **Duration**  12 weeks  **Location**  Naghavi Clinic in Kashan, Iran |
|  | Participants | **No of participants**  60  **Inclusion Criteria**  Quote: “overweight and/or obese (BMI >25 kg/m2 ) patients aged 18-40 years old with PCOS”  **PCOS Criteria**  Quote: “Diagnosis of PCOS was performed according to the Rotterdam criteria: those with the two of the following criteria were considered as having PCOS: (i) oligo- and/or anovulation (defined as delayed menses >35 days or 10 ml^3^).”  **Exclusion Criteria**  Quote: “We excluded women who were pregnant during the intervention, and adrenal hyperplasia, androgen-secreting tumours, hyperprolactinemia, thyroid dysfunction, diabetes or impaired glucose tolerance at enrolment.”  **Pre-conception intervention**  No |
|  | Interventions | **12 weeks Low-calorie DASH diet (n=30)**  Quote: “DASH eating pattern that was consisted of 52%-55% carbohydrates, 16%-18% proteins and 30% total fats. The DASH diet was rich in fruits, vegetables, whole grains and low-fat dairy products and low in saturated fats, cholesterol, refined grains and sweets. Suggested sodium intake in the DASH diet was <2400mg/d”  Quote: “both diets were designed to be calorie-restricted (350- 700 kcal less than the computed energy requirement for each person; 350 kcal for patients with the BMI in the range of 25-27.5 kg/ m2 ; 500 kcal for those with the BMI in the range of 27.5-31 kg/m2 and 700 kcal for those with the BMI >31 kg/m2. ”  **vs**  **12 weeks Low calorie control diet (n=30)**  Quote: “The control eating plan was also designed to contain 52%-55% carbohydrates, 16%-18% protein and 30% total fats23; however, DASH and control diets were different in terms of food groups contained”  Quote: “both diets were designed to be calorie-restricted (350- 700 kcal less than the computed energy requirement for each person; 350 kcal for patients with the BMI in the range of 25-27.5 kg/ m2 ; 500 kcal for those with the BMI in the range of 27.5-31 kg/m2 and 700 kcal for those with the BMI >31 kg/m2. ”  **Compliance**  Quote: “All participants provided 3-days dietary recalls and three physical activity records to verify that they maintained their usual diet and physical activity during the intervention.” |
|  | Outcomes | Serum AMH levels  Fasting glucose  Fasting insulin  HOMA-IR  HOMA-B  QUICKI  Total testosterone  SHBG  FAI  FSH  LH  17OH Progesterone  plasma nitric oxide (NO)  malondialdehyde (MDA) |
| Gan 2023 (60) | Methods | **Study Design**  Subset of patients from a randomised single-centre controlled trial  **Duration** 12 weeks  **Location**  Beijing, China |
|  | Participants | **No of participants**  29  **Inclusion Criteria**  Quote: “PCOS females aged 18–40 fulfilled the Rotterdam criteria; overweight or obese (body mass index ≥ 25kg/m2); no fertility plans within 6 months”  **PCOS Criteria**  Rotterdam criteria  **Exclusion Criteria**  Quote: “Anyone who meets any of the following conditions cannot participate in the study. (1) Combined with other endocrine diseases, such as diabetes, adrenal hyperplasia or tumor, Cushing’s syndrome, clinical thy- roid disease, acromegaly, hyperprolactinemia, androgen-secreting tumor, etc.; (2) Medullary thyroid disease patients with personal or family history of cancer (MTC) or patients with multiple endocrine neoplasia type 2 (MEN2); (3) combined with severe or unstable diseases, including liver, kidney, cardiovascular, respi- ratory, neurological, and blood system dysfunction; (4) with a history of thromboembolic disease or thrombosis; (5) heavy smoking or alcoholism women; (6) lactating or pregnant women; (7) women with a history of other malignant tumors; (8) Use of statins or other drugs known or suspected to affect repro- ductive or metabolic function within the past 3months; (9) Drug allergy; (10) Participating in other clinical trials.”  **Pre-conception intervention**  No |
|  | Interventions | **12 weeks Exenatide 2mg weekly with Metformin 500mg three times daily (n=14)**  Quote: “metformin (China-US Shanghai Squibb, SFDA Approval No. H20023371) 500mg orally, three times a day, combined with exenatide (AstraZeneca, USA, SFDA Approval No. H20170389) 2mg subcutaneously, once a week. All patients and healthy controls did not use antibiotics within 1month before collecting stool samples, and did not drink yogurt within the first 7days.”  **vs**  **12 weeks Metformin 500mg three times daily (n=15)**  Quote: “same metformin 500mg Ter in die. All patients and healthy controls did not use antibiotics within 1month before collecting stool samples, and did not drink yogurt within the first 7days.”  **Compliance**  Not specified |
|  | Outcomes | BMI  Total testosterone, dehydroepiandrosterone sulfate (DHAS), LH, FSH, E2  fasting glucose, OGTT 2 hour glucose, fasting insulin, HbA1c, HOMA-IR, triglyceride, HDL-c, LDL-c, ApoA1, ApoB |
| Ghandi 2011 (37) | Methods | **Study Design**  Randomized controlled trial  **Duration**  3 months  **Location**  Not mentioned |
|  | Participants | **No of participants**  40  **Inclusion Criteria**  Quote: “diagnosed to have PCOS, aged between 18–40, BMI ≥30 kg/m2 , no history of taking medication or dietary modification currently or for the preceding 3 months.”  **PCOS Criteria**  Quote: “The diagnosis of PCOS was made according to the revised 2003 European Society for Human Reproduction & Embryology / American Society for Reproductive medicine (ESHRE / ASRM) Rotterdam Criteria (Rotterdam ESHRE / ASRM— sponsored PCOS consensus workshop Group, 2004) with the presence of at least two of the following three features after exclusion of other etiologies: oligo- or anovulation, clinical and/ or biochemical hyperandrogenism and ultrasound finding of polycystic ovaries (presence of 12 or more follicles in each ovary measuring 2–9 millimeter in diameter, and / or increased ovarian volume (>10 ml).”  **Exclusion Criteria**  Quote: “eumenorrheic PCOS, presence of impaired fasting glycemia, untreated hypothyroidism, renal or hepatic impairment, hyperprolactinemia and nonclassical 21 – hydroxylase deficiency.”  **Pre-conception intervention**  No |
|  | Interventions | **3 months Metformin**  Quote: “The dose of metformin was increased step – wise, from 500 mg once daily for the first week to 500 mg twice daily for the next week, and to 500 mg three times daily for the remaining study period.”  **vs**  **3 months Orlistat**  Quote: “The dose of 120 mg orlistat was taken three times daily and the dose remained constant throughout the study period.”  **Compliance**  Not mentioned |
|  | Outcomes | Ovulation evaluated by serum progesterone level (>4 ng/ml) on day 21  Weight, BMI and waist circumference  Serum LH and total testosterone  Lipid profile |
| Harborne 2005 (36) | Methods | **Study Design**  Randomised controlled trial  **Duration**  8 months  **Location**  Royal Infirmary (Glasgow, UK) and surrounding hospitals |
|  | Participants | **No of participants**  83  **Inclusion Criteria**  Quote: “obese (body mass index, 30 to 37 kg/m2 ; n 42) and morbidly obese (body mass index, 37 kg/m2 ; n 41) women with PCOS.”  **PCOS Criteria**  Quote “at least two of the three features: oligomenorrhea (fewer than eight cycles per year)/amenorrhea (fewer than two cycles per year), polycystic ovaries determined by ultrasonography using the criteria of Adams et al. (12), or an elevated free androgen index.”  **Exclusion Criteria**  Quote: “Exclusion criteria included contraindications to metformin or its use within the previous 4 months or oral contraceptive use within the previous 2 months. None of the women had thyroid dysfunction, hyperprolactinemia, diabetes mellitus, or late-onset or congenital adrenal hyperplasia. Women taking medication known to affect weight loss, gonadal or adrenal function, or carbohydrate or lipid metabolism were also excluded. Women were advised to use barrier contraception and were excluded if interest was expressed in immediate pregnancy.”  **Pre-conception intervention**  No |
|  | Interventions | **8 months Metformin 1500 mg/d**  Quote: “Metformin (Glucophage, Merck & Co., West Drayton, UK) doses were 500 and 850 mg, three times daily.”  **vs**  **8 months Metformin 2550 mg/d**  Quote: “Metformin (Glucophage, Merck & Co., West Drayton, UK) doses were 500 and 850 mg, three times daily.”  **Compliance**  Not mentioned |
|  | Outcomes | Height, weight (BMI), and waist/hip ratio  Blood pressure, menstrual cyclicity, and hirsutism using the modified Ferriman-Gallwey score  Ovarian morphology and follicular growth (ovarian volume, numbers of follicles with diameter 10 mm, and diameter of the largest follicle).  fasting insulin, glucose, LH, FSH, estradiol, testosterone, SHBG, free androgen index, dehydroepiandrosterone sulfate (DHEAS), androstenedione, 17-hydroxyprogesterone, high sensitivity CRP, total cholesterol, triglycerides, low-density lipoprotein cholesterol (LDL-C), high-density lipoprotein cholesterol (HDL-C), leptin, IGF-I, and IGFbinding protein-3. Liver function, basic blood biochemistry, and thyroid function tests |
| Hoeger 2004 (32) | Methods | **Study Design**  Prospective, randomized, placebo-controlled pilot trial.  **Duration**  48 weeks  **Location**  Not mentioned |
|  | Participants | **No of participants**  38  **Inclusion Criteria**  Quote: “overweight or obese, with a minimum body mass index (BMI) of >25 mg/kg^2^”  Quote: “Normal TSH, prolactin, FSH, and metabolic profile were required for eligibility. Adrenal disease was evaluated according to DHEAS, serum 17-OH-progesterone, and 24- hour urinary free cortisol values. Values in the normal range for the clinical laboratory were required for eligibility.”  **PCOS Criteria**  Polycystic ovary syndrome was diagnosed by fewer than six menses per year and evidence of hyperandrogenism.  **Exclusion Criteria**  As per inclusion criteria  **Pre-conception intervention**  No |
|  | Interventions | **48 weeks Metformin (n=9)**  Quote: “metformin 850 mg by mouth two times per day”  **vs**  **48 weeks Lifestyle modification plus metformin (n=9)**  Quote: “lifestyle modification with metformin 850 mg by mouth two times per day”  Quote: “The lifestyle modification program was a comprehensive approach in the areas of nutrition, behavior, and physical activity. The lifestyle modification team consisted of a registered dietitian and exercise physiologist. Subjects were provided with an individualized healthy balanced meal plan with regular food, aiming for a 500–1000 calorie deficit per day. Average weight reduction goals were 7%–10%. Individualized exercise programs were recommended to achieve 150 minutes of exercise per week. The nutritional composition of the meals was 50% of calories from carbohydrate, 25% from protein, and 25% from fat. Intake of low glycemic index foods was encouraged. The program’s intensive phase was 24 weeks in duration, with weekly interactive group education meetings and progress monitoring. Two groups were started during the study, with 10 subjects per group. The maintenance phase was 24 weeks in duration, consisting of biweekly progress monitoring and group support meetings.”  **vs**  **48 weeks Lifestyle modification plus placebo (n=11)**  Quote: “a lifestyle modification program with placebo by mouth two times per day”  Quote: “The lifestyle modification program was a comprehensive approach in the areas of nutrition, behavior, and physical activity. The lifestyle modification team consisted of a registered dietitian and exercise physiologist. Subjects were provided with an individualized healthy balanced meal plan with regular food, aiming for a 500–1000 calorie deficit per day. Average weight reduction goals were 7%–10%. Individualized exercise programs were recommended to achieve 150 minutes of exercise per week. The nutritional composition of the meals was 50% of calories from carbohydrate, 25% from protein, and 25% from fat. Intake of low glycemic index foods was encouraged. The program’s intensive phase was 24 weeks in duration, with weekly interactive group education meetings and progress monitoring. Two groups were started during the study, with 10 subjects per group. The maintenance phase was 24 weeks in duration, consisting of biweekly progress monitoring and group support meetings.”  **vs**  **48 weeks Placebo alone (n=9)**  Quote: “placebo by mouth two times per day alone”  **Compliance**  Quote: “All subjects were given a 1-month supply of capsules and returned for monthly visits, at which time the remaining capsules were counted and a new supply of capsules given.” |
|  | Outcomes | Weight  Hirsutism  Total testosterone  SHBG  Free androgen index  OGTT  Fasting insulin  Fasting glucose  Ovulation |
| Jensterle 2015 (38) | Methods | **Study Design**  Open-label, randomized, prospective study  **Duration**  12 weeks  **Location**  Not mentioned |
|  | Participants | **No of participants**  32  **Inclusion Criteria**  Quote: “Women with diagnosed PCOS were eligible for enrollment if they were aged 18 years to menopause and were obese (body mass index: BMI ≥30). All patients had normal serum prolactin concentrations and thyroid function tests.”  **PCOS Criteria**  Quote: “diagnosed by the National Institute of Child Health and Human Development (NICHD) criteria. Clinical hyperandrogenism was definedby the presence of hirsutism, represented by a modified Ferriman-Gallwey (FG) score of eight or more, persistence of acne during the third decade of life or later or the presence of androgenic alopecia. No attemptswere made to grade the severity of acne or alopecia. Hyperandrogenemia was defined as a total or free testosterone, androstenedione and/or dehydroepiandrosterone sulphate (DHEAS) level above the 95th percentile of normal population values. Menstrual dysfunctionwas defined bymore than six cycles with a length of more than 35 days (oligomenorrhea) and/or when the patient had not had any menstrual bleeding for 3 consecutive months (amenorrhea) during the previous year.”  **Exclusion Criteria**  Quote: “known type 1 or type 2 diabetes mellitus, history of carcinoma, personal or family history of MEN 2, significant cardiovascular, kidney or hepatic disease and the use of medications known or suspected to affectreproductive or metabolic functions, or statins within 90 days prior to study entry. None of the patients had ever taken insulin-sensitizing drugs prior to the study. Possible Cushing’s syndrome or congenital (non-classic) adrenal hyperplasia were excluded”  **Pre-conception intervention**  No |
|  | Interventions | **12 week Liraglutide 1.2mg daily subcut (n=17)**  Quote: “liraglutide was initiated at a dose of 0.6 mg injected sc once per day and increased to 1.2 mg/day after 1 week.”  **vs**  **12 week Metformin 1000mg BD (n=15)**  Quote: “metformin was initiated at a dose of 500 mg once per day and increased by 500 mg every 3 days up to 1000 mg BID.”  **Compliance**  Not mentioned |
|  | Outcomes | height, weight, waist circumference and blood pressure.  whole-body composition  fasting glucose, insulin, HOMA-IR, Matsuda index  75 g oral glucose tolerance test (OGTT)  complete blood count, liver and renal function and serum electrolytes  lipid panel  total testosterone, free testosterone, SHBG, FAI |
| Jensterle 2016 (61) | Methods | **Study Design**  prospective randomized open-label design  **Duration**  12 weeks  **Location**  Ljubljana, Slovenia |
|  | Participants | **No of participants**  44  **Inclusion Criteria**  **“**obese women with PCOS (mean age, 30.3±.4.4 years; BMI, 37.2±4.5 kg/m2) diagnosed according to the American Society for Reproductive Medicine-European Society of Human Reproduction and Embryology (ASRM-ESHRE) Rotterdam criteria**” “**aged >18 years, had yet to undergo the menopause, and were obese [body mass index (BMI) ≥30]. **”**  **PCOS Criteria**  “All subjects had PCOS phenotype A, which included the concomitant presence of a) hyperandrogenemia at either the biochemical or clinical level, b) menstrual abnormalities, and c) PCO morphology. ”  **Exclusion Criteria**  “history of carcinoma, significant cardiovascular, kidney or hepatic disease, and the use of medications known to affect reproductive or metabolic functions prior to study entry. Certain subjects did, however, use oral contraceptives as advised by their gynecologists >6 months prior to being recruited. ”  **Pre-conception intervention**  No |
|  | Interventions | **12 week Metformin and Liraglutide (n=22)**  Quote: **“**Metformin was provided at a dose of 500 mg once daily and increased by 500 mg every 3 days until the treatment dose reached 1,000 mg twice daily”. “patients were treated with ≥1,000 mg twice daily metformin for 14 days prior to treatment with 1.2 mg LIRA once daily.”  **vs**  **12 week Liraglutide (n=22)**  Quote: “liraglutide treatment was provided at a dose of 0.6 mg subcutaneously injected once daily and increased to 1.2 mg/day after 1 week.”  **Compliance**  Not mentioned |
|  | Outcomes | Weight, BMI, waist circumference  Systolic BP, diastolic BP, fasting glucose, 2 hour glucose, fasting insulin, 2 hour insulin, HOMA-IR, total body fat, android/gynoid ratio, VAT mass  LH, FSH, Androstenedione, total testosterone, free testosterone, SHBG |
| Jensterle 2017 (62) | Methods | **Study Design**  Prospective randomized open-label design  **Duration**  12 weeks  **Location**  Ljubljana, Slovenia |
|  | Participants | **No of participants**  30  **Inclusion Criteria**  Quote: “type A phenotype of PCOS, age 18 years to menopause and obesity (body mass index: BMI ≥ 30).”  **PCOS Criteria**  Quote: “type A phenotype ASRM-ESHRE Rotterdam criteria including concomitant presence of a) hyper- androgenemia on either the biochemical or the clinical level, b) menses abnormalities and c) PCO morphology”  **Exclusion Criteria**  Quote: “Patients with history of carcinoma, significant cardiovascular, kidney or hepatic disease and the use of medications known to affect reproductive or metabolic functions within prior to study entry were excluded.”  **Pre-conception intervention**  No |
|  | Interventions | **12 weeks Metformin and Liraglutide (n=15)**  Quote: **“**metformin was initiated at a dose of 500 mg once per day and increased up to 1000 mg BID. Liraglutide was initiated at a dose of 0.6 mg injected s.c. once per day and increased to 1.2 mg”  **vs**  **12 weeks Liraglutide (n=15)**  Quote: “liraglutide was initiated at a dose of 0.6 mg injected s.c. once per day with increments to 3 mg”  **Compliance**  Not specified |
|  | Outcomes | Weight, BMI, waist circumference  Androstenedione, total testosterone, free testosterone, SHBG  Fasting glucose, OGTT 2 hour glucose, fasting insulin OGTT 2 hour insulin, HOMA IR, cholesterol, HDL, LDL, triglyceride |
| Jensterle 2023 (63) | Methods | **Study Design**  Single blind, placebo controlled trial  **Duration**  12 weeks  **Location**  Ljubljana, Slovenia |
|  | Participants | **No of participants**  20  **Inclusion Criteria**  Quote: “diagnosed with PCOS and obesity”  **PCOS Criteria**  Quote: “using the Rotterdam criteria, specifically, phenotype A, characterized as concomitant presence of irregular menstrual cycles, hyperandrogenism, and polycystic ovarian morphology ”  **Exclusion Criteria**  Quote: “any known serious chronic ill- ness, including diabetes mellitus, angina pectoris, coronary heart dis- ease, congestive heart failure, severe renal and hepatic impairment, inflammatory bowel disease, gastroparesis, cancer, chronic obstructive lung disease, psychiatric and neurological disease. Additional exclusion criteria were other clinical features, including GE disorders such as gas- troesophageal reflux, hypothyroidism, gastric resection, or medication that may alter GE (eg, metoclopramide, domperidone, cimetidine, para- sympatholytics and sympathomimetics). Given that metformin slows GE in patients with type 2 diabetes,23 10 subjects who had been treated with metformin before the enrolment were instructed to dis- continue metformin 1 month before the start of the study. Further exclusion criteria were: failure of the subject to ingest the entire meal; the use of medications that cause clinically significant weight gain or loss; previous bariatric surgery; history of idiopathic acute pancreatitis; family or personal history of multiple endocrine neoplasia type 2 or familial medullary thyroid carcinoma; current smoking; pregnancy, expecting pregnancy or breast feeding; and allergy to any of the ingredients in the study medication.”  **Pre-conception intervention**  No |
|  | Interventions | **Semaglutide (n=10)**  Quote: **“**semaglutide (Ozempyc; Novo Nordisk A/S, Bagsvaerd, Denmark) once weekly as s.c. injections in the abdomen.”  “Semaglutide was initiated at a dose of 0.25 mg once weekly for the first 2 weeks, was escalated by rapid titration to 0.5 mg/week for 2 weeks and then was increased to 1.0 mg once weekly for the remaining treatment period for 8 weeks.”  **vs**  **Placebo (n=10)**  Quote: “administered saline with prefilled saline syringes in the placebo pen using the same method and volume as the semaglutide group”  **Compliance**  Not specified |
|  | Outcomes | Weight, BMI, neck circumference, waist circumference  Systolic BP, diastolic BP, pulse  Glucose 0 min OGTT, glucose 120 min OGTT, insulin 0 min OGTT, insulin 120 min OGTT, HOMA-IR, HbA1c, cholesterol, HDL, LDL, triglycerides  FSH, LH, DHEAS, SHBG, FAI, total testosterone, free testosterone, androstenedione |
| Johnson 2015 (46) | Methods | **Study Design**  Parallel-group randomised controlled clinical trial  **Duration**  8 weeks  **Location**  southeast Norway |
|  | Participants | **No of participants**  61  **Inclusion Criteria**  18–40 years old treatment seeking morbidly obese women (either BMI R40 or 35–40 kg/m2 combined with at least one weight-related co-morbidity) (19) with PCOS diagnosed according to the Rotterdam Consensus Workshop Group Criteria  **PCOS Criteria**  according to the Rotterdam Consensus Workshop Group Criteria  **Exclusion Criteria**  Not specified  **Pre-conception intervention**  No |
|  | Interventions | **8 week Liquid meal replacement (LMR) (<1100 kcal/day) (n=31)** Quote “flavoured meal replacement shakes (nZ8/day, Nutrilett, Contract Foods Ltd, Redditch, Worcestershire, UK for Axellus AS, Oslo, Norway) with main ingredients soy protein, fructose (w9 g/shake) and soy fibres. Additionally, an unlimited intake of selected vegetables low in fibre (e.g. salads, cucumber, tomatoes, onions), !150 g root vegetables (e.g. carrots, cabbage, kohlrabi, broccoli, cauliflower) and one fruit (5–10 g fructose) per day were allowed. Fructose content of the LMR-diet was w85 g/day (w72 g from the LMR, w7g in one 150g fruit and w6g in 150g root vegetables plus 350 g tomatoes, salad and other low-fibre vegetables together). The women were encouraged to drink O2 l of water or other energy-free liquids daily in addition to the shakes.”  **vs**  **8 week Crisp bread diet (<1100 kcal/day) (n=30)**  Quote **“**The CB-diet was based on whole grain CB (provided by Wasa, Ideal Wasa AS) combined with low-fat, high-protein products for three of four daily meals. The CB-diet contained w17g fructose per day (w7g in one 150g fruit, w10 g in 850 g vegetables). Dinner consisted of a specified amount of fish, poultry or lean meat combined with vegetables, potatoes, rice or pasta. Participants were instructed to drink O1.5 l of water or other energy-free liquid per day. Supplement of one daily multivitamin and mineral pill was recommended (Collett Kostpluss, Axellus Oslo, Oslo, Norway). Additionally, participants could eat an unlimited amount of vegetables and one fruit per day. Estimated macronutrient intakes from fat, protein, total carbohydrate and fructose are shown in Table 1.”  **Compliance**  **Quote “**To strengthen motivation, answer possible questions and ensure dietary compliance, a registered dietician or a study nurse had weekly telephone contact with all participants.**”** |
|  | Outcomes | Body weight, BMI, waist circumference, hip circumference, neck circumference, fat mass, fat free mass  Glucose, HbA1c, HOMA-IR, insulin, C-peptide, total cholesterol, HDL, LDL, Triacylglycerol, Apolipoprotein-A1, Apolipoprotein-B, Apo-B/Apo-A1-ratio, LDL-cholesterol/Apo-B ratio, systolic BP, diastolic BP  Prolactin, SHBG, Estradiol, Testosterone, Free testosterone Index, FSH, LH, Progesterone, Androstenedione, Hirsutism  Uric acid, IGF 1, CRP, Fibrinogen, TSH, fT4, Gastrointestinal symptom rating scale (GSRS) |
| Kasim Karakas 2009 (47) | Methods | **Study Design**  Randomised single-blinded study  **Duration**  2 months  **Location**  United States |
|  | Participants | **No of participants**  33  **Inclusion Criteria “**patients with PCOS aged 18–45 years and with a body mass index of 25–40 kg/m2 **”**  **PCOS Criteria  “**by having ovarian dysfunction, as evidenced by amenorrhea (no periods for >6 months) or oligomenorrhea (<6 periods per year) and clinical (hirsutism) or laboratory evidence for hyperandrogenemia (total T >54 ng/dL or free T >9.2 pg/ mL). Ultrasound evaluation of the ovarian structure was not carried out. **”**  **Exclusion Criteria “**Adult-onset 21-hydroxylase deficiency was ruled out **” and “**Prolactinoma was ruled out by measuring the serum PRL concentration, and an- drogen-secreting tumors were ruled out on the basis of serum T. Cushing’s disease was ruled out clinically because the Rotterdam criteria require biochemical testing only when there is clinical suspicion. Patients were excluded if they used oral contraceptives, antiandrogenic medications, insulin sensitizers, d-chiro inositol, or any other medications or supplements that affect weight or insulin sensitivity during the preceding 2 months; had impaired glucose tolerance, diabe- tes mellitus, untreated hypothyroidism, and any other systemic illness, such as renal, hepatic, and gastrointestinal disease; were smokers; or drank more than two alcoholic drinks per week. **”**  **Pre-conception intervention**  No |
|  | Interventions | **Partial meal replacement: Hypocaloric diet with sugar-free whey protein isolate (n=17)**  “daily energy intake was decreased by 700 kcal from the habitual level without altering the dietary macronu- trient composition of the diet. ” “powdered supplements, providing 240 kcal and containing either sugar-free whey protein isolate (96% pure) ”  “Because whey is naturally calcium enriched, and calcium can independently promote weight loss (22), calcium contents of the supplements were equal- ized by adding tricalcium phosphate to the carbohydrate supplement. The whey protein supplement was sweetened by adding a non-calorie sugar substitute ”  **vs**  **Partial meal replacement: Hypocaloric diet with simple sugars (glucose plus maltose) supplements (n=16)**  “daily energy intake was decreased by 700 kcal from the habitual level without altering the dietary macronutrient composition of the diet. ” “powdered supplements, providing 240 kcal and containing simple sugars (glucose plus maltose)”  **Compliance**  Seven-day food records were obtained at study entry and once per month throughout the study. The data were analyzed with Food Processor software |
|  | Outcomes | Weight, BMI, fat mass, lean mass Glucose, Insulin, triglyceride, cholesterol, high-density lipoprotein (HDL) cholesterol, triglyceride, apoprotein B (apo B), leptin, adiponectin, HOMA, HgBA1, CRP Total Testosterone, sex hormone-binding globulin (SHBG), DHEAS, Free androgen index (FAI) |
| Lee 2023 (20) | Methods | **Study Design**  Randomised controlled trial  **Duration**  12 weeks  **Location**  Incheon, South Korea |
|  | Participants | **No of participants**  28  **Inclusion Criteria**  Quote: “women aged 18–40 years, who were diagnosed with PCOS based on the Rotterdam criteria, had a BMI greater than 25 kg/m2, and reported menstrual irregularities”  **PCOS Criteria**  Quote: “based on the Rotterdam criteria”  **Exclusion Criteria**  Quote: “Women who were pregnant with endocrine disorders, those taking insulin resistance-reducing drugs, and those with a history of eating disorders or significant weight change in the past year were excluded from the study.”  **Pre-conception intervention**  No |
|  | Interventions | **Lifestyle modification (n=14)**  The treatment group participated in a 12-week lifestyle modification program faciliitated by a mobile application developed by our research team. Upon registration, the participants were provided with an evidence-based booklet containing comprehensive information about PCOS, including details about the disease, symptoms, diet, and exercise. Throughout the 12 weeks, participants were required to enter various details daily into the mobile application, including intake amount, exercise duration, daily body weight, and gynecological information such as menstrual cycles, pregnancy, and other data. The application automatically calculated the total calories, exercise time, hirsutism, and acne scale scores based on the recorded data. The application was designed to enable users to complete questionnaires, access educational resources, and communicate with researchers for counseling. To motivate participation, users were provided with scores calls. The feedback aimed to assist participants in achieving their target exercise time of based on their progress towards their daily goals. The researcher reviewed these records an hour (or 10,000 steps of walk) with moderate intensity and maintaining their caloric and contacted each participant twice a week through mobile feedback messages or phone intake between 1400 and 1500 kcal/day. The prescribed macronutrient composition of the calls. The feedback aimed to assist participants in achieving their target exercise time of an hour (or 10,000 steps of walk) with moderate intensity and maintaining their caloric intake between 1400 and 1500 kcal/day. The prescribed macronutrient composition of the diet consisted of approximately 20% of total energy intake from protein, 30% from fat and 50% from carbohydrates. Emotional health was monitored using app notifications or text messages. Participants who successfully achieved their goals received positive feedback, whereas those who faced challenges were provided with feedback to encourage and motivate them. Furthermore, monthly phone calls or visits to the research centre were conducted to address any difficulties in using the application or following the lifestyle modification program.”  **vs**  **Usual care (n=14)**  Quote: “The control group was instructed to maintain their usual lifestyle and received an evidence-based leaflet containing information about PCOS at the beginning of the study.”  **Compliance**  Not mentioned |
|  | Outcomes | Weight  Hirsutism, Acne, Luteinizing hormone (LH), follicle-stimulating hormone (FSH), estradiol, testosterone, sex hormone-binding globulin (SHBG), and dehydroepiandrosterone sulfate (DHEA-S)  Fasting insulin, OGTT 2 hour insulin |
| Lindholm 2008 (33) | Methods | **Study Design**  Prospective, double-blind, multicenter, placebo-controlled study  **Duration**  24 weeks  **Location**  Quote: “multicenter study involving the gynecologic departments at Sunderby Hospital, Umea University Hospital, Uppsala University Hospital, L€akarhuset Bj€ornen, Pitea, and Lund University Hospital in Sweden.” |
|  | Participants | **No of participants**  42  **Inclusion Criteria**  Quote: “confirmed PCOS, age 18 to 40 years, body mass index (BMI) >27 kg/m2 , and consent to participation after written and oral information.”  Quote: “Diagnosis of PCOS also implied that no evidence of thyroid disease (normal serumTSH level), adrenocortical dysfunction (normal 17-hydroxyprogesterone level), or hyperprolactinemia (prolactin<30 ug/mL) was present. In addition, subjects had normal fasting levels of glucose, creatinine, aspartate aminotransferase, and alanine aminotransferase before inclusion.  **PCOS Criteria**  Quote: “Polycystic ovary syndrome was defined according to the Rotterdam criteria (33). Two of the following three features had to be present for the PCOS diagnosis: [1] oligomenorrhea with eight or fewer menstruations in the previous 12 months or amenorrhea; [2] clinical and/or biochemical signs of hyperandrogenism such as T >2.7 nmol/L, elevated DHEAS, free androgen index R1.0, or hirsutism (>7 on the Ferriman and Gallway scale); and [3] polycystic ovaries on ultrasound examination (>12 follicles 2 to 9 mm in diameter and/or increased ovarian volume (>10 mL).”  **Exclusion Criteria**  Quote: “obesity due to organic disorder, psychiatric disorder in need of medical treatment, pregnancy or lactation, use of hormonal treatments 6 months before inclusion in the study (oral contraceptives, gestagens, ovulatory stimulants, antidiabetic agents [including metformin], cortisone, antiandrogens), history of serious eating disorder (anorexia nervosa and/or bulimia nervosa), use of drugs with a central nervous system effect later than 2 weeks before study start (antidepressants, benzodiazepines, and antipsychotic drugs), history of coronary heart disease, heart failure, arrhythmia, tachycardia, peripheral arterial disease, history of stroke or a transient ischemic attack, uncontrolled hypertension (>140/90 mm Hg), severe renal or hepatic insufficiency, impaired liver function, Gilles de la Tourette’s syndrome, pheochromocytoma, glaucoma, and abuse of drugs (illegal or otherwise) or alcohol.”  **Pre-conception intervention**  No |
|  | Interventions | **24 weeks Sibutramine 15mg daily with lifestyle advice (n=21)**  Quote: “Lifestyle modification advice was given at the start of the study, but diet and physical activity were discussed with the patient at each visit as well.”  Quote: “Lifestyle modification advice was administered in a way that is possible to reproduce in regular clinical practice. All patients received lifestyle modification in form of diet advice and physical activity advice. Subjects were advised to eat three regular meals every day and to reduce the intake of fat in favor of carbohydrates. All subjects were given a step recorder and were encouraged to walk a minimum of 10,000 steps a day, most days of the week. They were also given homework assignments where food intake, reason for food intake, and physical activity were recorded. These records were repeated throughout the study and were reviewed together with the research nurse at every visit.”  **Vs**  **24 weeks Placebo with lifestyle advice (n=20)**  Quote: “Lifestyle modification advice was given at the start of the study, but diet and physical activity were discussed with the patient at each visit as well.”  Quote: “Lifestyle modification advice was administered in a way that is possible to reproduce in regular clinical practice. All patients received lifestyle modification in form of diet advice and physical activity advice. Subjects were advised to eat three regular meals every day and to reduce the intake of fat in favor of carbohydrates. All subjects were given a step recorder and were encouraged to walk a minimum of 10,000 steps a day, most days of the week. They were also given homework assignments where food intake, reason for food intake, and physical activity were recorded. These records were repeated throughout the study and were reviewed together with the research nurse at every visit.”  **Compliance**  Compliance was assessed by counting the remaining capsules at each visit |
|  | Outcomes | Height, weight, BMI, waist circumference, blood pressure  glucose, insulin,  apolipoprotein A-I (apo A-I), apolipoprotein B (apo B), triglycerides, high-sensitive C-reactive protein,  T, sex hormone–binding globulin (SHBG),  cystatin C, and creatinine  menstrual periods |
| Mehrabani 2012 (48) | Methods | **Study Design**  Randomised controlled trial  **Duration**  12 weeks  **Location**  Tehran, Iran |
|  | Participants | **No of participants**  60  **Inclusion Criteria**  Quote: “aged between 20 and 40 years, had a body mass index (BMI) greater than 25 and less than 38 kg/m2 , and no history of using an insulin-sensitizing agent such as metformin or oral contraceptives.”  **PCOS Criteria**  Quote: “menstrual irregularity (cycle length, ,21 days or .35 days), hirsutism, and biochemical hyperandrogenism.”  **Exclusion Criteria**  Quote: “Volunteers were excluded if they were smokers, exercised heavily, and/or had any history of cardiovascular, renal, gastrointestinal disorders, liver or metabolic diseases. Women diagnosed with hyperprolactinemia, thyroid abnormalities, and/or nonclassic adrenal hyperplasia were also excluded from this study.”  **Pre-conception intervention**  No |
|  | Interventions | **12 weeks Conventional hypocaloric diet (CHCD) (n= 26)**  Quote: “consisted of 55% of energy from carbohydrate, 15% of energy from protein, and 30% of energy from fat.”  **Vs**  **12 weeks modified hypocaloric diet (MHCD) high-protein, low-glycemic load (n=23)**  **Quote: ”**MHCD included 40% of energy from low-and medium-GL carbohydrates, 30% of energy from protein, and 30% of energy from fat, with limitation of high-glycemic foods. The MHCD group refrained from consuming high-GL foods (GL >= 20).**”**  **Compliance**  Quote: “A dietitian initially instructed the subjects on quantification and recording of their daily food intakes. They were followed weekly and asked to prepare dietary records for 3 days every month during the intervention (weeks 0, 4, 8, and 12) to enhance compliance and to enable the dietitian to monitor the prescribed diet.” |
|  | Outcomes | Weight, BMI, Waist and hip circumference  Total body fat, lean body mass  Follicle stimulating hormone (FSH), luteinizing hormone (LH), SHBG, estradiol, total testosterone, dehydroepiandrosterone sulfate (DHEAS), and androstenedione  Total cholesterol, triglyceride, LDL, HDL |
| Moeller 2019 (21) | Methods | **Study Design**  Randomized, controlled trial with two parallel arms  **Duration**  6 months  **Location**  Odense University Hospital, Denmark |
|  | Participants | **No of participants**  37  **Inclusion Criteria**  Quote: “PCOS diagnosed by the Rotterdam criteria and body mass index (BMI) >= 30 kg/m2 . Participants accepted to use barrier control as contraception during the study”  **Exclusion Criteria**  Quote: “Women were excluded if they used oral contraceptives or initiated metformin treatment within 3 months prior to study inclusion. Women with current pregnancy wish were excluded.”  **Pre-conception intervention**  No |
|  | Interventions | **6 months Motivational interviewing (MI) and standard advice (SA) (n=14)**  Quote: “SA was provided in accordance with guideline of Danish Board of Health”  Quote: “Participants were advised to decrease calorie intake to 1200–1500 calories per day and target a diet low in content of sugar and high in fiber rich vegetables and protein. At least 30 min of exercise per day was recommended. The SA included no extra appointments, and this group was therefore only seen at inclusion and follow-up.”  Quote: “In MI + SA group, women received MI by coauthor, CL, who is a certified member of Motivational Interview Network of Trainers (MINT). MI was conducted using a mix of face-to-face or Skype video interviews. Field notes and mind mapping were used to keep track of the interviews. MI was planned once every 2 weeks, that is a total of 12 times for each participant in MI group. Cancellation of MI was handled by text messaging instead. Follow up in out-patient clinic was arranged at end of study.”  **vs.**  **6 months standard advice (SA) (n=14)**  Quote: “SA was provided in accordance with guideline of Danish Board of Health”  Quote: “Participants were advised to decrease calorie intake to 1200–1500 calories per day and target a diet low in content of sugar and high in fiber rich vegetables and protein. At least 30 min of exercise per day was recommended. The SA included no extra appointments, and this group was therefore only seen at inclusion and follow-up.” |
|  | Outcomes | Weight  BMI  World Health Organization 5 well-being index (WHO-5)  Major Depression Inventory (MDI)  Short-Form-36 (SF-36)  Polycystic Ovary Syndrome Questionnaire (PCOS-Q).  Serum testosterone and serum lipids |
| Moini 2015 (58) | Methods | **Study Design**  randomized double-blind, placebo-controlled clinical trial.  **Duration**  3 months  **Location**  Arash Hospital, Iran |
|  | Participants | **No of participants**  100  **Inclusion Criteria**  Quote: “All patients were of reproductive age (19–38 years) and had a body mass index (BMI)>25. Study participants had no histories of taking hormonal medications in last six months, no current dietary modifications or dietary modifications for the preceding six months prior to study entry.”  **PCOS Criteria**  Quote: “according to Rotterdam Criteria (2004)”  **Exclusion Criteria**  Quote: “a history of cholestasis, liver disease, renal disease, malabsorption, or hypothyroidism”  **Pre-conception intervention**  No |
|  | Interventions | **3 months Orlistat**  Quote: “All patients received a hypocaloric diet that consisted of 55 % carbohydrates, 30 % fat, and 15 % protein. Each serving of this diet provided approximately 1,200–1,800 kilocalories per day according to each individual’s primary BMI. This is monounsaturated fatty acid (MUFA) diet—one of the diet protocols which is used in PCOS. The patients had normal physical activity and were encouraged to walk for 30 min daily. Participants completed weekly exercise diaries to monitor for compliance. The consistency of exercise was 77 % in the control group and 74.8 % in the intervention group. The intervention group received orlistat (120 mg) three times per day. The control group received a placebo.”  **Vs**  **3 months Placebo**  Quote: “All patients received a hypocaloric diet that consisted of 55 % carbohydrates, 30 % fat, and 15 % protein. Each serving of this diet provided approximately 1,200–1,800 kilocalories per day according to each individual’s primary BMI. This is monounsaturated fatty acid (MUFA) diet—one of the diet protocols which is used in PCOS. The patients had normal physical activity and were encouraged to walk for 30 min daily. Participants completed weekly exercise diaries to monitor for compliance. The consistency of exercise was 77 % in the control group and 74.8 % in the intervention group. The intervention group received orlistat (120 mg) three times per day. The control group received a placebo.” |
|  | Outcomes | Weight  BMI  Waist to hip ratio  Testosterone  Fasting insulin  Fasting glucose  HOMA-IR  Triglyceride  LDL  HDL |
| Moran 2003 (24)  Moran 2010 (49) | Methods | **Study Design**  Randomized controlled trial  **Duration**  16 weeks  **Location**  Not mentioned |
|  | Participants | **No of participants**  28  **Inclusion Criteria**  Overweight women (European Caucasian) with PCOS  **PCOS Criteria**  Quote: “diagnosis of PCOS by menstrual irregularity (cycle length, 21 d or 35 d or variation between consecutive cycles of 3 d) and clinical (hirsutism/acne) and/or biochemical hyperandrogenism. If a definition of menstrual irregularity as fewer than 9 menses/yr is used, 10 of 22 women fulfilled this criteria. However, this is an approximation, as a 12-month retrospective menses calendar was not available for all subjects, and 8 subjects were previously using hormonal medication. Hyperandrogenism was defined as a free androgen index (FAI) more than 2.85 (FAI testosterone/SHBG 100). This range was obtained from a representative population of non-PCOS women (n 80).”  **Exclusion Criteria**  Quote: “Exclusion criteria were inability to comply with study requirements, weight greater than 140 kg, smoking, and use of oral contraceptives/ hormone treatment/ Insulin-sensitizing agents. Subjects were eligible for the study if they had not been taking oral contraceptives for more than 4 wk or hormone treatment/insulin-sensitizing agents for more than 2 wk. Subjects with hyperprolactinemia, thyroid abnormalities, or nonclassic adrenal hyperplasia were excluded through appropriate hormone assessment.”  **Pre-conception intervention**  Yes |
|  | Interventions | **16 weeks Low protein diet**  Quote: “The dietary interventions were low protein (LP; 55% carbohydrate, 15% protein, and 30% fat) and high protein (HP; 40% carbohydrate, 30% protein, and 30% fat) with macronutrient composition calculated as a percentage of the total calories. Both diets were nutritionally complete, and alcohol intake was not permitted throughout the 16 wk. An energy-restricted diet (6000 kJ/d) was prescribed for 12 wk, followed by a weight maintenance diet for the final 4 wk with the same dietary composition adhered to in both phases (divide by 4.186 for conversion from kilojoules to kilocalories). Subjects attended a weekly exercise/education class (11, 12, 18) and were advised to increase exercise to a minimum of three times per week. Exercise levels were categorized according to National Health and Medical Research Council standards and were documented monthly and at baseline and study completion”  **Vs**  **16 weeks High protein diet**  Quote: “The dietary interventions were low protein (LP; 55% carbohydrate, 15% protein, and 30% fat) and high protein (HP; 40% carbohydrate, 30% protein, and 30% fat) with macronutrient composition calculated as a percentage of the total calories. Both diets were nutritionally complete, and alcohol intake was not permitted throughout the 16 wk. An energy-restricted diet (6000 kJ/d) was prescribed for 12 wk, followed by a weight maintenance diet for the final 4 wk with the same dietary composition adhered to in both phases (divide by 4.186 for conversion from kilojoules to kilocalories). Subjects attended a weekly exercise/education class (11, 12, 18) and were advised to increase exercise to a minimum of three times per week. Exercise levels were categorized according to National Health and Medical Research Council standards and were documented monthly and at baseline and study completion”  **Compliance**  Quote: “Subjects met with a registered dietitian fortnightly for initial education on quantification and recording of their daily food intake and to assess and modify the dietary regimen based on compliance and weight loss. Nutrient intakes were calculated with Diet 1/Nutrient Calculation software (Xyris Software, Highgate Hill, Australia) based on data from Australian food composition tables. Nutritional intake was assessed from monthly 3-d consecutive dietary food records (1 weekday and 2 weekend days) and daily dietary checklists. Dietary compliance was determined by subject adherence to the macronutrient profiles (protein, carbohydrate, and fat) and from assessment of random urine samples (wk 0, 12, and 16) for urea excretion relative to urinary creatinine.” |
|  | Outcomes | weight, body composition  dietary compliance  menstrual cyclicity, ovulation  hirsutism  fasting and postprandial glucose and insulin  surrogate measures of insulin sensitivity (homeostasis model of assessment)  lipid profile (Total cholesterol, low density lipoprotein cholesterol (LDL-C), high density lipoprotein cholesterol (HDL-C), triglycerides)  reproductive hormone profile (SHBG, total testosterone (bound and unbound), LH, FSH, progesterone, estradiol, TSH, PRL, and 17-hydroxyprogesterone)  insulin, and glucose, HOMA-IR |
| Moran 2006 (29) | Methods | **Study Design**  Randomised controlled trial  **Duration**  8 weeks  **Location**  Not mentioned |
|  | Participants | **No of participants**  43  **Inclusion Criteria**  Quote: “Overweight women (European whites) with PCOS”  **PCOS Criteria**  Quote: “diagnosis of PCOS, according to the Rotterdam ConsensusWorkshop Group, by 2 of the following 3 criteria: menstrual irregularity (cycle length < 26 d or > 31 d or variation between consecutive cycles of > 3 d); clinical (hirsutism assessed by a Ferriman-Gallwey score > 8) or biochemical [free androgen index (FAI) > 5.4 or testosterone > 1.4 nmol/L] hyperandrogenism; or positive ultrasound presentation of polycystic ovaries by transvaginal scan”  **Exclusion Criteria**  Quote: “pregnancy, breastfeeding, body mass index (BMI; in kg/m2 ) 25, type 2 diabetes mellitus, and related endocrinopathic disorders [identified by assessment of thyroid-stimulating hormone (TSH), prolactin, and 17- hydroxyprogesterone]. The use of endocrine hormonal treatment or insulin-sensitizing agents was not permitted during either phase of the study, and the use of oral contraceptives was not permitted during phase 1 of the study. Subjects were required to cease taking oral contraceptives 4 wk and hormonal treatment or insulin-sensitizing agents 2 wk before commencement of the short-term study phase. From weeks 8 –32 (phase 2), subjects were allowed to take oral contraceptives with containing 35g ethinyl estrogen.”  **Pre-conception intervention**  Yes |
|  | Interventions | 8 week weight-loss with 6 month weight-maintenance carbohydrate restriction regimen  Quote: “In phase 1 (weeks 0 – 8), subjects followed an energy-restricted diet in which 2 meals/d were replaced with commercially available meal replacements (Slimfast; Unilever Australasia, Epping, Australia) that were provided fortnightly). Alcohol consumption was not permitted. For phase 2 (weeks 9 –32), subjects followed either a CC (120 g carbohydrate/d) or an FC (50 g fat/d) regimen. Subjects were provided with a CC or FC resource and asked to document their daily intake of carbohydrate or fat from selected foods. All subjects also received advice on reducing the GI and the saturated fat content of their diet. A semi–ad libitum approach was followed. Subjects were allowed to consume their specified fat (50 g) or carbohydrate (120 g) intake. In addition, subjects were advised to eat until full from unlimited quantities of additional foods whose fat or carbohydrate they were not required to count. Subjects were allowed to consume moderate amounts of alcohol (2 standard drinks/d and 2 alcohol-free days/wk).”  Vs  8 week weight-loss with 6 month weight-maintenance fat restriction regimen  Quote: “In phase 1 (weeks 0 – 8), subjects followed an energy-restricted diet in which 2 meals/d were replaced with commercially available meal replacements (Slimfast; Unilever Australasia, Epping, Australia) that were provided fortnightly). Alcohol consumption was not permitted. For phase 2 (weeks 9 –32), subjects followed either a CC (120 g carbohydrate/d) or an FC (50 g fat/d) regimen. Subjects were provided with a CC or FC resource and asked to document their daily intake of carbohydrate or fat from selected foods. All subjects also received advice on reducing the GI and the saturated fat content of their diet. A semi–ad libitum approach was followed. Subjects were allowed to consume their specified fat (50 g) or carbohydrate (120 g) intake. In addition, subjects were advised to eat until full from unlimited quantities of additional foods whose fat or carbohydrate they were not required to count. Subjects were allowed to consume moderate amounts of alcohol (2 standard drinks/d and 2 alcohol-free days/wk).”  **Compliance**  Quote: “In phase 1, nutritional intake was assessed from fortnightly 3-d consecutive dietary food records (1 weekday and 2 weekend days) and daily dietary checklists. For prestudy dietary intake and during phase 2, nutritional intake and GI and GL were assessed from 3-mo food-frequency questionnaires (AntiCancer Foundation, Carlton, Australia) and a monthly 24-h dietary recall. Dietary compliance was ascertained by subject adherence to the meal-replacement regimen in phase 1 and to daily fat and carbohydrate counting in phase 2.” |
|  | Outcomes | Weight  Blood pressure  plasma glucose, insulin, ghrelin, and serum lipid concentrations of C-reactive protein (CRP), testosterone, and SHBG  Menstrual cycle, ovulation  Pregnancy |
| Munir 2018 (35) | Methods | **Study Design**  Randomised controlled trial  **Duration**  12 weeks  **Location**  Shalamar Hospital Lahore, Pakistan |
|  | Participants | **No of participants**  45  **Inclusion Criteria**  Women who are diagnosed suffering from PCOS  BMI ≥ 30kg/m2  Age between 18-40years  Not taking any medication that alters hypothalamic pituitary axis  **PCOS Criteria**  The diagnosis of PCOS was made according to the revised European society for Human Reproduction and Embryology/American society for reproductive Medicine (ESHRE/ASRM) Rottermdam criteria 2004 with presence of at least two of the following three features after exclusion of other etiologies through history, clinical examination and laboratory investigations if needed, oligomenorrhoea or amenorrhoea, anovulation, clinical and biochemical features of hyperandrogenism (Hirsutism, Acne) and ultrasound finding of polycystic ovaries that is presence of 12 or more follicles in each ovary measuring 2- 9mm in diameter and/or increased ovarian volume >10ml.  **Exclusion Criteria**  Patients with hepatic or renal impairment  Malabsorption syndrome, cholestasis  Pregnancy, breast feeding mothers  **Pre-conception intervention**  No |
|  | Interventions | **12 weeks Orlistat 120 BD + low caloric diet exercise**  Quote: “It consisted hypocaloric balanced diet of 1400kcal/day with 5-10%fats. A printed diet plan was given to the patients.30minutes/day of moderate intensity aerobic exercise (e.g.,) brisk walk, cycling or tread meal.”  **Vs**  **12 weeks Low caloric diet and exercise**  Quote: “It consisted hypocaloric balanced diet of 1400kcal/day with 5-10%fats. A printed diet plan was given to the patients.30minutes/day of moderate intensity aerobic exercise (e.g.,) brisk walk, cycling or tread meal.” |
|  | Outcomes | Weight, height and waist circumference, BMI  Serum LH, progesterone Testosterone & SHBG  Baseline and day 21 serum progesterone level >4ng/ml as taken sign of ovulation |
| Nybacka 2011 (50)  Nybacka 2013 (51)  Nybacka 2017 (52) | Methods | **Study Design**  Randomized controlled trial  **Duration**  4 months  **Location**  Women's Health Research Unit, Karolinska University Hospital, Stockholm, Sweden |
|  | Participants | **No of participants**  57  **Inclusion Criteria**  Quote: “all three PCOS criteria according to the Rotterdam Consensus (i.e., oligo- or anovulation, hyperandrogenism, and polycystic ovaries on ultrasound) (14) and also the following criteria: age 18–40 years; body mass index (BMI) >27 kg/m2 ; absence of hormonal treatment for the last 3 months; and no pregnancy, lactation, or change in weight during the past year.”  **PCOS Criteria**  Quote: “all three PCOS criteria according to the Rotterdam Consensus (i.e., oligo- or anovulation, hyperandrogenism, and polycystic ovaries on ultrasound)”  **Exclusion Criteria**  Quote: “the presence of other disease or a different endocrine disorder; an eating disorder; smoking; or continuous medication”  **Pre-conception intervention**  No |
|  | Interventions | **4 month Diet (n=19)**  Quote: “The diets were designed individually under close supervision of a dietician (A.N.). It was recommended that total daily caloric intake be reduced by at least 600 kcal/d in comparison with preintervention intake, while maintaining a wellbalanced diet containing 55–60E% carbohydrates, 25–30E% fat (10E% saturated), and 10–15E% proteins, according to Swedish nutritional recommendations in 2005 (15). A strict schedule of three main meals and two or three snacks was also introduced. Food intake was assessed by self-reporting once every 24 hours during 4 days immediately before and at the end of intervention.”  **Vs**  **4 month Exercise (n=19)**  Quote: “The exercise program, which was supervised by a physiotherapist, was based on each individual's condition, interest, and experience and aimed at increasing physical exercise to a moderate level. The exercise included walking (with or without poles), aerobics, jogging, swimming, and muscle strength training, with a moderate to vigorous exertion level, performed two to three times per week and a duration of 45– 60 minutes each time during the intervention period. Physical activity was assessed using pedometers (Yamax SW-200) during the 4 days immediately before and at the end of the program.”  **Vs**  **Diet and exercise (n=19)**  Quote: “The diets were designed individually under close supervision of a dietician (A.N.). It was recommended that total daily caloric intake be reduced by at least 600 kcal/d in comparison with preintervention intake, while maintaining a wellbalanced diet containing 55–60E% carbohydrates, 25–30E% fat (10E% saturated), and 10–15E% proteins, according to Swedish nutritional recommendations in 2005 (15). A strict schedule of three main meals and two or three snacks was also introduced. Food intake was assessed by self-reporting once every 24 hours during 4 days immediately before and at the end of intervention. The exercise program, which was supervised by a physiotherapist, was based on each individual's condition, interest, and experience and aimed at increasing physical exercise to a moderate level. The exercise included walking (with or without poles), aerobics, jogging, swimming, and muscle strength training, with a moderate to vigorous exertion level, performed two to three times per week and a duration of 45– 60 minutes each time during the intervention period. Physical activity was assessed using pedometers (Yamax SW-200) during the 4 days immediately before and at the end of the program.” |
|  | Outcomes | BMI, fat %, body fat, body mass  LH, FSH, T, SHBG, fT, AMH  DHEAS, 17OHP  IGF-1, IGFBP-1, Insulin, Glucose, HOMA index  Mean follicle no, mean ovarian volume, menstrual cyclicity, ovulation  Waist circumference, systolic blood pressure, diastolic blood pressure, HOMA index, insulin AUC, glucose AUC, insulinogenic index, hsCRP  Total cholesterol, LDL, HDL, Triglycerides |
| Oberg 2019 (22) | Methods | **Study Design**  Randomised controlled trial  **Duration**  4 months  **Location**  Karolinska University Hospital, Stockholm, Sweden |
|  | Participants | **No of participants**  68  **Inclusion Criteria**  Quote: “Women aged 18‐40 years, with a body mass index (BMI) of at least 27 kg/m2 and fulfilling all three PCOS diagnostic criteria according to the Rotterdam Consensus”  **PCOS Criteria**  Quote: “all three PCOS diagnostic criteria according to the Rotterdam Consensus of having oligomenorrhea or amenorrhoea, displaying polycystic ovaries on a transvaginal ultrasound scan and having clinical or biochemical hyperandrogenism were eligible to enter the study”  **Exclusion Criteria**  Quote: “taking regular medication, another ongoing medical condition, smoking, pregnancy or breastfeeding, having a history of an eating disorder or a substantial weight change during the past year. A wash‐out period of 3 months was used if taking hormonal contraceptives. Several women had a desire to become pregnant, but all women accepted to use nonhormonal contraception during the first 4 months of the study.”  **Pre-conception intervention**  Yes |
|  | Interventions | **4 months Behavioural modification programme**  Quote: “s consisted of a structured approach to achieve long‐term weight control for improvement of reproductive and metabolic function, as well as quality of life. Patients underwent a formal course in small groups together with fellow study partici‐ pants. Group meetings were held three times a month throughout the intervention. The course included up to date knowledge con‐ cerning weight control, personal leadership, mindfulness and infor‐ mation regarding physical activity and diet and was delivered by a lifestyle coach (PTE) with a PhD in endocrinology and metabolism. The patients were given reading material22 and homework to reflect upon before each group meeting, which then formed the basis for group discussions about goal setting, stimulus control, problem‐ solving, stress management and techniques to avoid the temptation of instant gratification. In addition, the patients received individu‐ alized coaching sessions once a month with the course leader to discuss suitable individual training regimens, diet changes and to ensure compliance with the intervention as well as offer personal support.”  **vs**  **4 months Minimal intervention**  Quote: “The control group received an intervention designed to reflect or‐ dinary patient care and consisted of general healthy lifestyle rec‐ ommendations given by a research midwife and supported by a pamphlet with written advice about diet and exercise. During both types of intervention, monthly visits were sched‐ uled for control of weight, waist/hip measurements and vital signs. The women in both groups were also encouraged to carry an accelerometer, ActiGraph GT3X, for 7 days at baseline and at 4 months that measured the energy expenditure and step count of the patients. In addition, all patients were given a protein dink free of charge, Natural Balance Shake, Formulation Pre Mix™ (Indevex Biotech(TM)®, Växsjö, Sweden) containing 65 kcal, which they were encouraged to drink 30 minutes before each major meal (breakfast, lunch and dinner) to reduce hunger feelings” |
|  | Outcomes | Quote: “Our primary reproductive outcome measure was improved menstrual regularity at 4 months defined as shifting from having amenorrhoea to either oligomenorrhea or regular cycles, or from oligomenorrhea to regular cycles. Information on this was ob‐ tained from menstrual diaries or apps. Amenorrhoea was defined as no spontaneous menstrual bleeding during the last 3 months, oligomenorrhea as a cycle length greater than 35 days but shorter than 3 months and regular menstrual periods as a cycle length of 21‐35 days. Secondary outcomes were initiation of ovulation at 4 months, and improved menstrual regularity, initiation of ovula‐ tion and regular menstrual periods at 12 months, all as defined above. In addition, we report pregnancies within 1 year of study completion.” |
|  | Methods | **Study Design**  Randomised controlled trial  **Duration**  24 weeks  **Location**  Not mentioned |
| Pandurevic 2023 (53) | Methods | **Study Design**  Randomised controlled open-label trial  **Duration**  8 weeks  **Location**  Bologna, Italy |
|  | Participants | **No of participants**  30  **Inclusion Criteria** Quote: “women of a reproductive age (18–45 years) and with a BMI between 28 and 40 kg/ m2 ”  **PCOS Criteria**  Quote: “according to the NIH criteria included the presence of oligo/amenorrhea with hirsutism (modified Ferriman-Gallwey-mFG ≥ 8) and/or hyperandrogenemia (free androgen index (FAI) ≥ 4), after excluding other causes of hyperandrogenism)”  **Exclusion Criteria**  Quote: “any of the following: type 1 diabetes mellitus, type 2 diabetes mellitus treated with insulin, obesity caused by endocrine diseases unrelated to PCOS or drug-induced obesity, any weight loss diet or estro-progestin therapy up to 3 months preceding the start of the trial, severe depression or other psychiatric disorder, alcohol and drug abuse, severe hepatic insufficiency, any level of renal insufficiency or calculosis of the urinary tract, gout, malignancy, previous cardio- or cerebrovascular events, uncontrolled hypertension, electrolytic disturbances, any drug capable of interfering with glucose or steroid metabolism, menopause, pregnancy, breastfeeding, and refusal to sign the informed consent form.”  **Pre-conception intervention**  No |
|  | Interventions | **8 weeks very low calorie diet (VLCKD) followed by 8 weeks low calorie diet (LCD) (n=15)**  Quote: **“**The VLCKD phase consisted of three steps of 600–800 kcal/day with high-biological-value protein preparations obtained from cow’s milk, soy, eggs, green peas, and cereals, <50 g daily of carbohydrates from vegetables, and 10 g of olive oil per day. In step 1, which lasted for 6 weeks, the patients ate high biological value protein preparations five times a day and vegetables with a low glycemic index. In step 2 (1 week), one of the protein servings was substituted with a natural protein (e.g. meat or fish) either at lunch or at dinner. In the step 3 (1 week), a second serving of a low-fat natural protein replaced the second serving of a biological protein preparation. Throughout these steps, supplements of vitamins and minerals were provided, such as K, Na, Mg, Ca, and omega-3 fatty acids. The LCD phase consisted of a gradual incorporation of different food groups with a daily calorie intake rising progressively from 1050 to 1400 kcal/day. In this phase, carbohydrates were gradually reintroduced in the following order: foods with the lowest glycemic index (fruit and dairy products), followed by foods with a moderate glycemic index (legumes), and finally by foods with a high glycemic index (bread, pasta, and cereals). The experimental group was instructed to do muscle tone exercises during the 8 weeks of VLCKD, while both groups were instructed to do aerobic and muscle tone exercises during the time on LCD, following the exercise tables of the Pronokal® method.”  **Vs Mediterranean low calorie diet (n=15)**  Quote: “The caloric intake in the control group ranged from 1200 to 1420 kcal/day, with 15, 30, and 55% contributions from proteins, lipids, and carbohydrates, respectively. The dietary energy intake was calculated by subtracting 500 kcal from the usual individual energy intake, according to the LARN guidelines. Habitual energy intake was estimated based on the diet history method and a 3-day recall questionnaire. Both groups were instructed to do aerobic and muscle tone exercises during the time on LCD, following the exercise tables of the Pronokal® method”  **Compliance**  Not mentioned |
|  | Outcomes | BMI, waist circumference, fast mass, fat-free mas  Fasting glucose, fasting insulin, HOMA-IR, total cholesterol, HDL, LDL, triglycerides, AST, ALT, systolic blood pressure, diastolic blood pressure, heart rate  Total testosterone, free testosterone, SHBG, Ovulation, Hirsutism (total hair count, hair thickness, hair density, cumulative hair index at chin/thorax/lower abdomen), ovarian volume, stromal to total area ratio of the largest ovary  Psychological distress (anxiety, depression, somatization, hostility-irritability, total distress, total score psychological wellbeing scale) |
| Samarasinghe 2024 (39) | Methods | **Study Design**  Open label randomised controlled trial  **Duration**  1 year  **Location**  United Kingdom |
|  | Participants | **No of participants**  80  **Inclusion Criteria**  Quote: “women older than 18 years, with a diagnosis of PCOS based on the 2018 international evidence-based guidelines for assessing and managing PCOS,11 and a BMI of 35 kg/m2 or higher. ”  **PCOS Criteria**  Quote: “based on the 2018 international evidence-based guidelines for assessing and managing PCOS”, “Oligomenorrhoea or amenorrhoea was defined as a cycle length of less than 21 days or more than 35 days, or fewer than eight cycles in the preceding 12 months or no menstruation.”  **Exclusion Criteria**  Quote: “having diabetes, being unable to maintain effective non-hormonal contraception, current pregnancy or breastfeeding, and gastro-oesophageal reflux disease. We followed national guidance by the British Obesity Metabolic Surgery Society, according to which, women should not conceive for 12–18 months after bariatric surgery.”  **Pre-conception intervention**  No |
|  | Interventions | **Laparoscopic sleeve gastrectomy (n=40)**  Quote: **“**Laparoscopic sleeve gastrectomy was performed using a standardised technique at both sites (appendix p 4). Participants attended group sessions led by dietitians, nurses, and psychologists before surgery. At these sessions, participants were informed about the eating behaviour and psychological changes that take place after surgery. Postoperatively, individuals were assessed by a clinical nurse specialist at 10 days, 6 months, and 12 months, and by a dietitian at 3 months. Specific attention was placed on adequate contraception and monitoring for surgical, medical, nutritional, and psychological complications. Participants had access to psychological interventions if that was considered necessary.” “Two weeks before either intervention, participants were administered oral medroxyprogesterone acetate 10 mg once daily for 10 days to induce a withdrawal bleed and to ensure that all participants started the intervention on the follicular phase of their menstrual cycle.”  **vs**  **Medical care (n=40)**  Quote: “Medical care was based on the 2018 international evidence-based guidelines.11 Lifestyle modification was provided by specialist dietitians at online group sessions and one-to-one consultations. The frequency of these sessions was every 2 weeks for the first 26 weeks of the trial and subsequently once every 4 weeks for the remaining 26 weeks. Specific attention was placed on wellbeing and the psychological features of PCOS. Participants were offered oral pharmacotherapy, including metformin (maximum dose 1 g twice daily; oral administration), orlistat (maximum dose 120 mg three times a day; oral administration), or a combination of the two. The study was performed before glucagon- like peptide 1 receptor analogues (GLP-1 receptor agonists) became widely available in the UK NHS. ”  “Two weeks before either intervention, participants were administered oral medroxyprogesterone acetate 10 mg once daily for 10 days to induce a withdrawal bleed and to ensure that all participants started the intervention on the follicular phase of their menstrual cycle.”  **Compliance**  Not mentioned |
|  | Outcomes | Spontaneous ovulatory events  Weight, percentage total bodyweight loss, body fat percentage)  Waist circumference, systolic and diastolic blood pressure, fasting glucose, fasting insulin, glycated haemoglobin, homeostatic model assessment for insulin resistance [HOMA-IR], glucose concentration incremental area under the curve, cholesterol, triglycerides, non-HDL, HDL, LDL, liver enzymes) reproductive (follicle stimulating hormone, oestradiol, luteinising hormone, sex hormone binding globulin, testosterone, free androgen index, dehydroepiandrosterone sulphate, androstenedione, anti-Müllerian hormone, self-reported menses), number of medications, androgenic (Ferriman- Gallwey hirsutism score, Ludwig visual score of androgenic alopecia, Savin alopecia scale score, Cardiff Acne Disability index) quality of life (Polycystic Ovary Syndrome Health-Related Quality of Life Questionnaire [PCOSQ ] score Hospital Anxiety scale Hospital Depression scale RAND 36-Item Short Form Health Survey [SF-36]) |
| Stamets 2004 (54) | Methods | **Study Design**  Randomized clinical trial.  **Duration**  1 month  **Location**  Academic medical center. |
|  | Participants | **No of participants**  35  **Inclusion Criteria**  Quote: “Participants were selected based on a diagnosis of PCOS: based on a history of chronic anovulation (=< six spontaneous menstrual cycles per year) and unexplained elevated circulating T levels”  Quote: “between the ages of 21 and 37”  Quote: “All participants were obese, which was defined as a body mass index (BMI) 25 kg/m2 (19). All women were in good health and, for at least 1 month before the study, were not taking any medication (except for oral contraceptive agents which were stopped 3 months prior to the study) known to affect sex hormone levels, carbohydrate metabolism, or appetite. The participants were required to be nonsmokers and to exercise no more than three times per week.”  **Exclusion Criteria**  Quote: “Other causes of androgen excess (e.g., nonclassical adrenal 21-hydroxylase deficiency, hyperprolactinemia, and androgen-secreting tumors) were excluded by appropriate testing. Ultrasonography was not performed as part of the study because the presence or absence of polycystic ovaries has not been included in consensus criteria for the definition of the endocrine syndrome of PCOS (2). None of the participants had been diagnosed previously with diabetes mellitus.”  **Pre-conception intervention**  No |
|  | Interventions | **1 month high protein**  Quote**: “**high protein (HP: 30% protein, 40% carbohydrate, and 30% fat. The fat content was held constant in both diets. Each participant’s energy needs were calculated with the Harris– Benedict equation using an adjusted body weight for obesity and an activity factor of 1.5. Energy needs were then adjusted to create a 1,000-kcal deficit per day to produce an approximately 1,000 g/week rate of weight loss. The study dietitian translated the diet into practical food choices for the participants using limited exchange lists from the American Diabetes Association Exchange Lists for Meal Planning. Specific instructions were not given about types of carbohydrates or glycemic indices. All participants were instructed to consume a multivitamin/mineral supplement daily. During the initial encounter, the dietitian met with each of the participants to describe the diet and its options, as well as to answer any questions the participants may have had. To ensure compliance with the diet, participants completed daily self-monitoring diet charts and reported their weight all of which they faxed to the dietitian weekly. They also filled out satiety questionnaires and spoke with the dietitian weekly, reviewing their charts and answering questions related to each participant’s prescribed diet plan.**”**  **vs**  **1 month high carbohydrate**  Quote**: “**high carbohydrate (HC: 15% protein, 55% carbohydrate, and 30% fat). The fat content was held constant in both diets. Each participant’s energy needs were calculated with the Harris– Benedict equation using an adjusted body weight for obesity and an activity factor of 1.5. Energy needs were then adjusted to create a 1,000-kcal deficit per day to produce an approximately 1,000 g/week rate of weight loss. The study dietitian translated the diet into practical food choices for the participants using limited exchange lists from the American Diabetes Association Exchange Lists for Meal Planning. Specific instructions were not given about types of carbohydrates or glycemic indices. All participants were instructed to consume a multivitamin/mineral supplement daily. During the initial encounter, the dietitian met with each of the participants to describe the diet and its options, as well as to answer any questions the participants may have had. To ensure compliance with the diet, participants completed daily self-monitoring diet charts and reported their weight all of which they faxed to the dietitian weekly. They also filled out satiety questionnaires and spoke with the dietitian weekly, reviewing their charts and answering questions related to each participant’s prescribed diet plan.**”** |
|  | Outcomes | Height, weight, waist/hip girth ratio, and Ferriman–Gallwey measurements  3-hour OGTT and fasting T, uT, PRL, 17-OH P, DHEAS, LH, FSH, total cholesterol, HDL, LDL, and serum triglyceride levels, glucose |
| Veena Kirthika 2019 (56) | Methods | **Study Design**  Randomised controlled trial  **Duration**  24 weeks  **Location**  the Faculty of physiotherapy Dr MGR educational and research institute Deemed to be university |
|  | Participants | **No of participants**  24  **Inclusion Criteria**  Quote: “age group between 18-25 years diagnosed with PCOS based on the Rotterdam criteria and BMI range between 25-29”  **Exclusion Criteria**  Quote “Subjects with thyroid disease, prolactin excess, non-classical congenital adrenal hyperplasia, glucocorticoid dysfunction, subjects under anti hypersensitivity medications and Lipid lowering medications were excluded from the study.”  **Pre-conception intervention**  No |
|  | Interventions | **Group A (n=12) 24 week PRE +aerobic exercises +diet**  Quote: “Supervised PRE exercise session was carried out for 2 days in a week on consecutive days for 24 weeks at the Faculty of Physiotherapy. The exercise session lasted for 60 minutes including standardized (5 minute) warm-up and cool-down. The exercise protocol followed were lateral pull down, leg curl, seated row, calf raise, chest press, split squat, shoulder press, biceps curl, triceps extension and abdominal curl. Exercises like chest press, shoulder press, biceps curl, and triceps extension were also performed. All sets of exercise (except abdominal curl) were performed to neuromuscular fatigue i.e. 8-12 repetitions maximum. Two sets of each exercise were given in the first 2 weeks. From week 3, all exercises except split squat and shoulder press was progressed to 3 sets. The subjects also performed calisthenic exercises on non PRE days, 4 days in a week which included lying external hip rotations (‘clam shells’), side leg raises, push-ups on knees, wall squats, oblique curls and core stabilization exercises (‘bird dog’ and abdominal hollowing), performed for 3 sets x 10 repetitions each. The numbers of repetitions of each exercise performed were recorded. Additionally the subjects performed moderate intensity aerobic exercises in the form of brisk walking in a tread mill for 30 minutes in a day for 5 days in a week. Dietary advice from a nutritionist, which consisted of high protein, low fat and carbohydrate diet, was given to all the subjects.”  **vs**  **group B (n=12) 24 week aerobic exercises + diet**  Quote: “The subjects performed moderate intensity aerobic exercises in the form of brisk walking for 30 minutes a day for 5 days in a week. Dietary advice from a nutritionist was given to all the subjects.” |
|  | Outcomes | BMI, PCOSQ and hormonal levels (SHBG, Free testosterone, HOMA IR, Hs CRP). |
| Vigorito 2007 (23) | Methods | **Study Design**  Prospective baseline-randomized clinical study  **Duration**  3 month  **Location**  University “Federico II” of Naples, School of Medicine (Italy) |
|  | Participants | **No of participants**  90  **Inclusion Criteria**  Quote: “overweight nonsmoking women with PCOS were enrolled in the study protocol.”  **PCOS Criteria**  Quote “All the PCOS patients achieved the European Society for Human Reproduction and Embryology/American Society for Reproductive Medicine criteria for the PCOS diagnosis (19). Polycystic ovaries were identified by transvaginal ultrasonography examination (20) and hirsutism by Ferriman-Gallwey score greater than 8”  **Exclusion Criteria**  Quote: “pregnancy, glucose intolerance (as screened by a 2-h oral glucose tolerance test) and diabetes, hypothyroidism, hyperprolactinemia, Cushing’s syndrome, nonclassical congenital adrenal hyperplasia, and use of oral contraceptives, glucocorticoids, antiandrogens, ovulation induction agents, antidiabetic or antiobesity drugs, or other hormonal drugs within the previous 6 months. Subjects with neoplastic, hepatic, respiratory, and any cardiovascular disorder or other concurrent medical illness (i.e. heart failure, lung or renal disease) were also excluded from the study. None of the study patients drank alcoholic beverages.”  **Pre-conception intervention**  No |
|  | Interventions | **PCOS-T: Exercise (n=45)**  Quote: “a 3-month structured ET program on a hospital ambulatory-based regimen. Training sessions, performed three times per week under continuous electrocardiographic monitoring, were supervised by a cardiologist, a physiotherapist, and a graduate nurse. Each session was preceded by a 5-min warm-up and followed by a 5-min cool-down. Exercise was performed for 30 min on a bicycle ergometer with the target of 60 –70% of the maximal oxygen consumption (VO2max) achieved at the initial cardiopulmonary exercise test monitored by a wearable device. Exercise workload was gradually increased until the achievement of the predefined target.”  Quote: “general dietary and behavioral advice without a structured caloric restriction program was given to the entire PCOS study population. All of the PCOS population was counseled to achieve a healthy balanced meal plan with regular food with a nutritional composition of 50% of calories from carbohydrate, 25% from protein, and 25% from fat. Intake of low glycemic index foods was encouraged.”  **Vs**  **PCOS UnT: No exercise (n=45)**  Quote: “general dietary and behavioral advice without a structured caloric restriction program was given to the entire PCOS study population. All of the PCOS population was counseled to achieve a healthy balanced meal plan with regular food with a nutritional composition of 50% of calories from carbohydrate, 25% from protein, and 25% from fat. Intake of low glycemic index foods was encouraged.” |
|  | Outcomes | hormonal assessment (Plasma LH, FSH, prolactin, estradiol, progesterone, 17-hydroxyprogesterone, testosterone, androstenedione, and dehydroepiandrosterone sulfate levels), lipid profile, and fasting glucose and insulin levels, cardiovascular and endocrinological examination, 12-lead electrocardiography, transvaginal ultrasonography, cardiopulmonary exercise test, anthropometric measurements, including height, weight, body mass index (BMI), waist circumference, waist/ hip ratio (WHR), and leisure-time physical activity (LTPA) questionnaire |
| Vosnakis 2012 (55) | Methods | **Study Design**  Randomised controlled trial  **Duration**  7 month  **Location**  the Division of Endocrinology and Human Reproduction outpatient clinic |
|  | Participants | **No of participants**  76  **Inclusion Criteria**  Quote: “premenopausal, non-pregnant, non-lactating, overweight and obese women (body mass index (BMI) > 27), 18 years old with PCOS”  **PCOS Criteria:**  Quote: “Diagnosis of PCOS was based on the 2003 Rotterdam ESHRE/ASRM-Sponsored PCOS Consensus criteria”  **Exclusion Criteria**  Quote: “Women with non-classical 21-hydroxylase deficiency, hyperprolactinemia, adrenal or ovarian tumor, Cushing’s disease, hypertension, thyroid dysfunction, overt diabetes mellitus and concomitant treatment were excluded.”  **Pre-conception intervention**  No |
|  | Interventions | **7 months of hypocaloric diet, physical exercise plus sibutramine**  Quote: “all patients were placed on a hypocaloric diet, physical exercise plus sibutramine (10 mg per day) for the first month and then on a hypocaloric diet plus sibutramine (10 mg per day) or a hypocaloric diet only for the subsequent 6 months. Moderate physical activity (3 h per week) and a normal-protein, energy restricted diet were prescribed based on a calculated individual basal metabolic rate (BMR) of all women (BMR – 600 kcal/day), for a period of 6 months.”  **vs**  **1 month hypocaloric diet, physical exercise plus sibutramine then 6 months hypocaloric diet plus exercise** |
|  | Outcomes | Weight, waist and hip circumferences  AMH, FSH, LH, PRL, T, D4A, DHEAS, 17a-hydroxyprogesterone (17a-OHP), SHBG, glucose, and insulin  changes of number of follicles (expressed as the mean number of follicles of both ovaries = follicles of right ovary + follicles of left ovary/2)  mean ovarian volume (expressed as the mean volume of both ovaries = volume of right ovary + volume of left ovary/2) |
