# Supplementary material for: Pre‐conception weight loss interventions in women with polycystic ovary syndrome and the effect on perinatal outcomes: A quantitative synthesis of surrogate outcomes
Source: Diabetes Obes Metab. 2025 Oct 1;27(12):7158–79. doi: 10.1111/dom.70116 (PMC12587233; doi:10.1111/dom.70116)
Supplement: Supplementary file 4 — Data S4. Supporting Information. [file DOM-27-7158-s003.docx]

**Supplementary Material 4**

***Table 4.*** *Risk of bias table: review authors' judgements about each risk of bias*

| Study | Bias | Judgement | Support for Judgement |
| --- | --- | --- | --- |
| Asemi 2014 | **Random sequence generation (selection bias)** | Low risk | The investigators describe a random component in the sequence generation process  Quote: “stratified according to BMI (<30 and > 30) and age (<30 and > 30) and then were randomly assigned to the control (n = 27) or the DASH diet (n = 27) for 8 wk. Random assignment was done by the use of computer-generated random numbers.” |
|  | **Allocation concealment (selection bias)** | Low risk | Participants and investigators enrolling participants could not foresee assignment |
|  | **Blinding of participants and personnel (performance bias)** | High risk | No blinding or incomplete blinding, and the outcome is likely to be influenced by lack of blinding |
|  | **Blinding of outcome assessment (detection bias)** | Low risk | No blinding of outcome assessment, but the review authors judge that the outcome measurement is not likely to be influenced by lack of blinding  Quote “with the exception of the study dietitian, all study personnel and participants were blinded to dietary assignment” |
|  | **Incomplete outcome data (attrition bias)** | Low risk | Missing outcome data balanced in numbers across intervention groups, with similar reasons for missing data across group  The attrition rate was 3/27 in the intervention group and 3/27 in the control group. Intention-to-treat analysis was performed. |
|  | **Selective reporting (reporting bias)** | Unclear risk | Insufficient information to permit judgement |
|  | **Other bias** | Low risk | Listed author has had other articles on DASH diet listed for retraction |
| Atiomo 2009 | **Random sequence generation (selection bias)** | Low risk | The investigators describe a random component in the sequence generation process  Quote: “women were then randomised to 600kcal deficit low glycaemic diet or a 600 kcal deficit hypocaloric healthy eating approach using a web-based randomisation programme designed for the study” |
|  | **Allocation concealment (selection bias)** | Low risk | Participants and investigators enrolling participants could not foresee assignment |
|  | **Blinding of participants and personnel (performance bias)** | High risk | No blinding or incomplete blinding, and the outcome is likely to be influenced by lack of blinding |
|  | **Blinding of outcome assessment (detection bias)** | Low risk | No blinding of outcome assessment, but the review authors judge that the outcome measurement is not likely to be influenced by lack of blinding  Quote: “clinician and radiologist assessing the subjects at month 6 were blind to the allocation groups. The biochemist, histopathologist and statistician evaluating the endocrine and metabolic assays, endometrial biopsies and analysing the data were also blind to the allocations” |
|  | **Incomplete outcome data (attrition bias)** | Low risk | Missing outcome data balanced in numbers across intervention groups, with similar reasons for missing data across group |
|  | **Selective reporting (reporting bias)** | Unclear risk | Insufficient information to permit judgement |
|  | **Other bias** | Low risk | The study appears to be free of other sources of bias. |
| Azadi-Yazdi 2017 | **Random sequence generation (selection bias)** | Low risk | The investigators describe a random component in the sequence generation process  Quote: “Equally randomised into DASH diet and control diet groups using block randomisation. Random assignment was conducted using computer-generated random numbers (DASH group, control group).” |
|  | **Allocation concealment (selection bias)** | Low risk | Participants and investigators enrolling participants could not foresee assignment |
|  | **Blinding of participants and personnel (performance bias)** | High risk | No blinding or incomplete blinding, and the outcome is likely to be influenced by lack of blinding |
|  | **Blinding of outcome assessment (detection bias)** | High risk | No blinding of outcome assessment, and the outcome measurement is likely to be influenced by lack of blinding |
|  | **Incomplete outcome data (attrition bias)** | Low risk | Missing outcome data balanced in numbers across intervention groups, with similar reasons for missing data across group  The attrition rate was 2/30 in the intervention group and 3/30 in the control group. Intention-to-treat analysis was not performed. |
|  | **Selective reporting (reporting bias)** | Unclear risk | Insufficient information to permit judgement |
|  | **Other bias** | Low risk | The study appears to be free of other sources of bias. |
| Bruno 2007 | **Random sequence generation (selection bias)** | High risk | The investigators describe a non-random component in the sequence generation process.  Quote: “The odd numbered patients, according to the order of arrival, constituted the 2.5 g group (receiving 2.5 g of metformin per day), and the even patients constituted the 1.5 g group (receiving 1.5 g of metformin per day).” |
|  | **Allocation concealment (selection bias)** | High risk | Participants or investigators enrolling participants could possibly foresee assignments and thus introduce selection bias |
|  | **Blinding of participants and personnel (performance bias)** | Unclear risk | Insufficient information to permit judgement |
|  | **Blinding of outcome assessment (detection bias)** | Unclear risk | Insufficient information to permit judgement |
|  | **Incomplete outcome data (attrition bias)** | Unclear risk | Insufficient information to permit judgement |
|  | **Selective reporting (reporting bias)** | Unclear risk | Insufficient information to permit judgement |
|  | **Other bias** | Unclear risk | Insufficient information to permit judgement |
| Cincione 2023 | **Random sequence generation (selection bias)** | Unclear risk | Insufficient information about the sequence generation process to permit judgement |
|  | **Allocation concealment (selection bias)** | Unclear risk | Insufficient information to permit judgement |
|  | **Blinding of participants and personnel (performance bias)** | Unclear risk | Insufficient information to permit judgement |
|  | **Blinding of outcome assessment (detection bias)** | Unclear risk | Insufficient information to permit judgement |
|  | **Incomplete outcome data (attrition bias)** | Low risk | Missing outcome data balanced in numbers across intervention groups, with similar reasons for missing data across group |
|  | **Selective reporting (reporting bias)** | Unclear risk | Insufficient information to permit judgement |
|  | **Other bias** | Unclear risk | Insufficient information to permit judgement |
| deLoos 2021  deLoos 2022  deLoos 2023  Jiskoot 2020 | **Random sequence generation (selection bias)** | Low risk | The investigators describe a random component in the sequence generation process  Quote: “women were randomly assigned in a 1:1:1 ratio to one of the three arms of the study: SMS+, SMS−, or CAU. Randomization was performed using a computer-generated random numbers table.” |
|  | **Allocation concealment (selection bias)** | Low risk | Participants and investigators enrolling participants could not foresee assignment |
|  | **Blinding of participants and personnel (performance bias)** | High risk | No blinding or incomplete blinding, and the outcome is likely to be influenced by lack of blinding |
|  | **Blinding of outcome assessment (detection bias)** | Unclear risk | Insufficient information to permit judgement |
|  | **Incomplete outcome data (attrition bias)** | Low risk | Missing outcome data balanced in numbers across intervention groups, with similar reasons for missing data across group  The attrition rate was 44/60 in the SMS+ group, 26/60 in the SMS- group and 36/60 in the control group. Intention-to-treat analysis was performed. |
|  | **Selective reporting (reporting bias)** | Unclear risk | Insufficient information to permit judgement |
|  | **Other bias** | Unclear risk | Insufficient information to permit judgement |
| Deshmukh | **Random sequence generation (selection bias)** | Low risk | The investigators describe a random component in the sequence generation process  “Eligible participants were randomised on a 1:1 ratio using an online web-based randomisation service” |
|  | **Allocation concealment (selection bias)** | Low risk | Participants and investigators enrolling participants could not foresee assignment |
|  | **Blinding of participants and personnel (performance bias)** | High risk | No blinding or incomplete blinding, and the outcome is likely to be influenced by lack of blinding |
|  | **Blinding of outcome assessment (detection bias)** | Unclear risk | Insufficient information to permit judgement |
|  | **Incomplete outcome data (attrition bias)** | Low risk | Missing outcome data balanced in numbers across intervention groups, with similar reasons for missing data across group |
|  | **Selective reporting (reporting bias)** | Unclear risk | Insufficient information to permit judgement |
|  | **Other bias** | Unclear risk | Insufficient information to permit judgement |
| Elkind-Hirsch 2022 | **Random sequence generation (selection bias)** | Low risk | The investigators describe a random component in the sequence generation process  Quote: “Participants were randomly allocated (2:1) on the basis of computer-generated random numbers using the block randomization method for treatment” |
|  | **Allocation concealment (selection bias)** | Low risk | Participants and investigators enrolling participants could not foresee assignment |
|  | **Blinding of participants and personnel (performance bias)** | Low risk | Blinding of participants and key study personnel ensured, and unlikely that the blinding could have been broken  Quote: “LIRA 3 mg and PL were provided by Novo Nordisk A/S in identical prefilled pens labeled with serial numbers and accompanied by a dispensing unit list”  Quote: “participants, investigators, and those assessing outcomes were blinded to drug assignment.” |
|  | **Blinding of outcome assessment (detection bias)** | Low risk | Blinding of outcome assessment ensured, and unlikely that the blinding could have been broken.  Quote: “participants, investigators, and those assessing outcomes were blinded to drug assignment.” |
|  | **Incomplete outcome data (attrition bias)** | Low risk | Missing outcome data balanced in numbers across intervention groups, with similar reasons for missing data across group  The attrition rate was 11/55 in the Liraglutide group and 4/27 in the Placebo group. Intention-to-treat analysis was performed. |
|  | **Selective reporting (reporting bias)** | Low risk | All pre-specified outcomes are reported. |
|  | **Other bias** | Low risk | The study appears to be free of other sources of bias. |
| Esfahanian 2013 | **Random sequence generation (selection bias)** | Low risk | The investigators describe a random component in the sequence generation process  Quote: “The block randomization method was designed to randomized participants into Metformin or hypocaloric diet groups.”  Quote: “Subjects were randomized into one of two arms by random number assignment” |
|  | **Allocation concealment (selection bias)** | High risk | Participants or investigators enrolling participants could possibly foresee assignments and thus introduce selection bias |
|  | **Blinding of participants and personnel (performance bias)** | Unclear risk | Insufficient information to permit judgement |
|  | **Blinding of outcome assessment (detection bias)** | Unclear risk | Insufficient information to permit judgement |
|  | **Incomplete outcome data (attrition bias)** | Unclear risk | Insufficient information to permit judgement |
|  | **Selective reporting (reporting bias)** | Unclear risk | Insufficient information to permit judgement |
|  | **Other bias** | Unclear risk | Insufficient information to permit judgement |
| Florakis 2008 | **Random sequence generation (selection bias)** | Low risk | The investigators describe a random component in the sequence generation process  Quote: “A randomization table was created using blocks of three numbers with all possible combinations, to achieve the randomization 2:1 ratio to S and D groups. A random number generator was used to keep balance between treatment groups.” |
|  | **Allocation concealment (selection bias)** | Low risk | Participants and investigators enrolling participants could not foresee assignment |
|  | **Blinding of participants and personnel (performance bias)** | High risk | No blinding or incomplete blinding, and the outcome is likely to be influenced by lack of blinding |
|  | **Blinding of outcome assessment (detection bias)** | Unclear risk | Insufficient information to permit judgement |
|  | **Incomplete outcome data (attrition bias)** | Unclear risk | Insufficient information to permit judgement |
|  | **Selective reporting (reporting bias)** | Unclear risk | Insufficient information to permit judgement |
|  | **Other bias** | Unclear risk | Insufficient information to permit judgement |
| Foroozanfard 2017 | **Random sequence generation (selection bias)** | Low risk | The investigators describe a random component in the sequence generation process eg. computer-generated random numbers. |
|  | **Allocation concealment (selection bias)** | Low risk | Participants and investigators enrolling participants could not foresee assignment |
|  | **Blinding of participants and personnel (performance bias)** | High risk | No blinding or incomplete blinding, and the outcome is likely to be influenced by lack of blinding  Quote: “With the exception of the study dietitian (Z.A.), who provided the dietary education, all the study personnel and subjects were blinded to dietary assignment.” |
|  | **Blinding of outcome assessment (detection bias)** | Low risk | Blinding of outcome assessment ensured, and unlikely that the blinding could have been broken.  Quote: “With the exception of the study dietitian (Z.A.), who provided the dietary education, all the study personnel and subjects were blinded to dietary assignment.” |
|  | **Incomplete outcome data (attrition bias)** | Low risk | Missing outcome data balanced in numbers across intervention groups, with similar reasons for missing data across group  The attrition rate was 4/30 in the DASH diet group and 3/30 in the Control diet group. Intention-to-treat analysis was performed. |
|  | **Selective reporting (reporting bias)** | Unclear risk | Insufficient information to permit judgement |
|  | **Other bias** | Low risk | The study appears to be free of other sources of bias. |
| Gan 2023 | **Random sequence generation (selection bias)** | Low risk | The investigators describe a random component in the sequence generation process  Quote: “According to the method of random number table, the patients were randomly divided into two treatment groups according to the ratio of 1:1” |
|  | **Allocation concealment (selection bias)** | Low risk | Participants and investigators enrolling participants could not foresee assignment |
|  | **Blinding of participants and personnel (performance bias)** | High risk | No blinding or incomplete blinding, and the outcome is likely to be influenced by lack of blinding |
|  | **Blinding of outcome assessment (detection bias)** | Unclear risk | Insufficient information to permit judgement |
|  | **Incomplete outcome data (attrition bias)** | Unclear risk | Insufficient information to permit judgement |
|  | **Selective reporting (reporting bias)** | Unclear risk | Insufficient information to permit judgement |
|  | **Other bias** | Unclear risk | Insufficient information to permit judgement |
| Ghandi 2011 | **Random sequence generation (selection bias)** | Low risk | The investigators describe a random component in the sequence generation process  Quote: “Randomization was performed using random number table.” |
|  | **Allocation concealment (selection bias)** | Low risk | Participants and investigators enrolling participants could not foresee assignment |
|  | **Blinding of participants and personnel (performance bias)** | Unclear risk | Insufficient information to permit judgement |
|  | **Blinding of outcome assessment (detection bias)** | Unclear risk | Insufficient information to permit judgement |
|  | **Incomplete outcome data (attrition bias)** | Unclear risk | Insufficient information to permit judgement |
|  | **Selective reporting (reporting bias)** | Unclear risk | Insufficient information to permit judgement |
|  | **Other bias** | Unclear risk | Insufficient information to permit judgement |
| Harborne 2005 | **Random sequence generation (selection bias)** | Low risk | The investigators describe a random component in the sequence generation process  Quote: “dose-block randomization in two groups of patients with PCOS, defined as Ob (BMI, >=30 and <37 kg/m2 ) and Mob (BMI, >37 kg/m2 ).” |
|  | **Allocation concealment (selection bias)** | High risk | Participants or investigators enrolling participants could possibly foresee assignments and thus introduce selection bias |
|  | **Blinding of participants and personnel (performance bias)** | High risk | No blinding or incomplete blinding, and the outcome is likely to be influenced by lack of blinding  Quote: “Patients were not blinded to treatment dose.” |
|  | **Blinding of outcome assessment (detection bias)** | Unclear risk | Insufficient information to permit judgement |
|  | **Incomplete outcome data (attrition bias)** | Low risk | Missing outcome data balanced in numbers across intervention groups, with similar reasons for missing data across group  Quote: “Sixty-eight patients (82%) completed the course to the final 8 month assessment point. There was no difference in the proportions of patients completing the study in each group. Intention to treat analysis was performed.” |
|  | **Selective reporting (reporting bias)** | Unclear risk | Insufficient information to permit judgement |
|  | **Other bias** | Unclear risk | Insufficient information to permit judgement |
| Hoeger 2004 | **Random sequence generation (selection bias)** | Low risk | The investigators describe a random component in the sequence generation process  Quote: “The randomization schedule was computer generated in blocks by an independent pharmacy representative” |
|  | **Allocation concealment (selection bias)** | High risk | Participants or investigators enrolling participants could possibly foresee assignments and thus introduce selection bias |
|  | **Blinding of participants and personnel (performance bias)** | Low risk | Blinding of participants and key study personnel ensured, and unlikely that the blinding could have been broken  Quote: “Identically appearing placebo capsules were also formulated. Drug and placebo were packaged and labeled according to subject number by the pharmacy in a double-blind fashion.” |
|  | **Blinding of outcome assessment (detection bias)** | Low risk | Blinding of outcome assessment ensured, and unlikely that the blinding could have been broken  Quote: “the block schedule was blinded to the investigators” |
|  | **Incomplete outcome data (attrition bias)** | Unclear risk | Insufficient information to permit judgement |
|  | **Selective reporting (reporting bias)** | Unclear risk | Insufficient information to permit judgement |
|  | **Other bias** | Low risk | The study appears to be free of other sources of bias. |
| Jensterle 2015 | **Random sequence generation (selection bias)** | Low risk | The investigators describe a random component in the sequence generation process  Quote: “As a method of randomization the RAND programme in Excel was used.” |
|  | **Allocation concealment (selection bias)** | Low risk | Participants and investigators enrolling participants could not foresee assignment |
|  | **Blinding of participants and personnel (performance bias)** | High risk | No blinding or incomplete blinding, and the outcome is likely to be influenced by lack of blinding |
|  | **Blinding of outcome assessment (detection bias)** | Unclear risk | Insufficient information to permit judgement |
|  | **Incomplete outcome data (attrition bias)** | Low risk | Missing outcome data balanced in numbers across intervention groups, with similar reasons for missing data across group  Quote: “Twenty-eight (87.5%) patients (14 on LIRA and 14 on MET) finished the study according to the protocol.” |
|  | **Selective reporting (reporting bias)** | Unclear risk | Insufficient information to permit judgement |
|  | **Other bias** | Unclear risk | Insufficient information to permit judgement |
| Jernsterle 2016 | **Random sequence generation (selection bias)** | Unclear risk | Insufficient information about the sequence generation process to permit judgement |
|  | **Allocation concealment (selection bias)** | Unclear risk | Insufficient information to permit judgement |
|  | **Blinding of participants and personnel (performance bias)** | High risk | No blinding or incomplete blinding, and the outcome is likely to be influenced by lack of blinding |
|  | **Blinding of outcome assessment (detection bias)** | Unclear risk | Insufficient information to permit judgement |
|  | **Incomplete outcome data (attrition bias)** | Low risk | Missing outcome data balanced in numbers across intervention groups, with similar reasons for missing data across group |
|  | **Selective reporting (reporting bias)** | Unclear risk | Insufficient information to permit judgement |
|  | **Other bias** | Unclear risk | Insufficient information to permit judgement |
| Jensterle 2017 | **Random sequence generation (selection bias)** | Unclear risk | Insufficient information about the sequence generation process to permit judgement |
|  | **Allocation concealment (selection bias)** | Unclear risk | Insufficient information to permit judgement |
|  | **Blinding of participants and personnel (performance bias)** | High risk | No blinding or incomplete blinding, and the outcome is likely to be influenced by lack of blinding |
|  | **Blinding of outcome assessment (detection bias)** | Unclear risk | Insufficient information to permit judgement |
|  | **Incomplete outcome data (attrition bias)** | Low risk | Missing outcome data balanced in numbers across intervention groups, with similar reasons for missing data across group |
|  | **Selective reporting (reporting bias)** | Unclear risk | Insufficient information to permit judgement |
|  | **Other bias** | Unclear risk | Insufficient information to permit judgement |
| Jensterle 2023 | **Random sequence generation (selection bias)** | Low risk | The investigators describe a random component in the sequence generation process  Quote: “As a method of randomization the RAND programme in Excel was used.” |
|  | **Allocation concealment (selection bias)** | Low risk | Participants and investigators enrolling participants could not foresee assignment |
|  | **Blinding of participants and personnel (performance bias)** | Low risk | Blinding of participants and key study personnel ensured, and unlikely that the blinding could have been broken |
|  | **Blinding of outcome assessment (detection bias)** | High risk | No blinding of outcome assessment, and the outcome measurement is likely to be influenced by lack of blinding |
|  | **Incomplete outcome data (attrition bias)** | Low risk | Missing outcome data balanced in numbers across intervention groups, with similar reasons for missing data across group |
|  | **Selective reporting (reporting bias)** | Unclear risk | Insufficient information to permit judgement |
|  | **Other bias** | Low risk | The study appears to be free of other sources of bias. |
| Johnson 2015 | **Random sequence generation (selection bias)** | Low risk | The investigators describe a random component in the sequence generation process  Quote: “Participants were randomized in blocks of six (three participants to each group) ” |
|  | **Allocation concealment (selection bias)** | High risk | Participants or investigators enrolling participants could possibly foresee assignments and thus introduce selection bias |
|  | **Blinding of participants and personnel (performance bias)** | Unclear risk | Insufficient information to permit judgement |
|  | **Blinding of outcome assessment (detection bias)** | High risk | No blinding of outcome assessment, and the outcome measurement is likely to be influenced by lack of blinding;  Quote: “Group allocation was revealed to participant and staff member (registered dietician or study nurse) after all baseline examinations and measures were completed. “ |
|  | **Incomplete outcome data (attrition bias)** | Low risk | Missing outcome data balanced in numbers across intervention groups, with similar reasons for missing data across group |
|  | **Selective reporting (reporting bias)** | Unclear risk | Insufficient information to permit judgement |
|  | **Other bias** | Unclear risk | Insufficient information to permit judgement |
| Kasim Karakas 2009 | **Random sequence generation (selection bias)** | Low risk | The investigators describe a random component in the sequence generation process  Quote: “randomized using block design provided by the Research Randomizer ” |
|  | **Allocation concealment (selection bias)** | High risk | Participants or investigators enrolling participants could possibly foresee assignments and thus introduce selection bias |
|  | **Blinding of participants and personnel (performance bias)** | Low risk | Blinding of participants and key study personnel ensured, and unlikely that the blinding could have been broken |
|  | **Blinding of outcome assessment (detection bias)** | High risk | No blinding of outcome assessment, and the outcome measurement is likely to be influenced by lack of blinding; |
|  | **Incomplete outcome data (attrition bias)** | Low risk | Missing outcome data balanced in numbers across intervention groups, with similar reasons for missing data across group |
|  | **Selective reporting (reporting bias)** | Unclear risk | Insufficient information to permit judgement |
|  | **Other bias** | Unclear risk | Insufficient information to permit judgement |
| Lee 2023 | **Random sequence generation (selection bias)** | Low risk | The investigators describe a random component in the sequence generation process  Quote: “Randomization was conducted by the research coordinator, who independently per-  Randomization was conducted by the research coordinator, who independently per-  formed simple randomization using an online software called ‘research randomizer’ before  formed simple randomization using an online software called ‘research randomizer’ be-  data collection.” |
|  | **Allocation concealment (selection bias)** | Low risk | Participants and investigators enrolling participants could not foresee assignment |
|  | **Blinding of participants and personnel (performance bias)** | High risk | No blinding or incomplete blinding, and the outcome is likely to be influenced by lack of blinding |
|  | **Blinding of outcome assessment (detection bias)** | Unclear risk | Insufficient information to permit judgement |
|  | **Incomplete outcome data (attrition bias)** | Low risk | Missing outcome data balanced in numbers across intervention groups, with similar reasons for missing data across group |
|  | **Selective reporting (reporting bias)** | Unclear risk | Insufficient information to permit judgement |
|  | **Other bias** | Unclear risk | Insufficient information to permit judgement |
| Lindholm 2008 | **Random sequence generation (selection bias)** | Unclear risk | Insufficient information about the sequence generation process to permit judgement |
|  | **Allocation concealment (selection bias)** | Unclear risk | Insufficient information to permit judgement |
|  | **Blinding of participants and personnel (performance bias)** | Low risk | Blinding of participants and key study personnel ensured, and unlikely that the blinding could have been broken  Quote: “During the study, the subjects and study personnel were not informed about which treatment the patient received.” |
|  | **Blinding of outcome assessment (detection bias)** | Low risk | Blinding of outcome assessment ensured, and unlikely that the blinding could have been broken.  Quote: “During the study, the subjects and study personnel were not informed about which treatment the patient received.” |
|  | **Incomplete outcome data (attrition bias)** | Low risk | Missing outcome data balanced in numbers across intervention groups, with similar reasons for missing data across group |
|  | **Selective reporting (reporting bias)** | Unclear risk | Insufficient information to permit judgement |
|  | **Other bias** | Low risk | The study appears to be free of other sources of bias. |
| Mehrabani 2012 | **Random sequence generation (selection bias)** | Unclear risk | Insufficient information about the sequence generation process to permit judgement |
|  | **Allocation concealment (selection bias)** | Unclear risk | Insufficient information to permit judgement |
|  | **Blinding of participants and personnel (performance bias)** | Low risk | Blinding of participants and key study personnel ensured, and unlikely that the blinding could have been broken  Quote: “The investigator was not blinded as to the kind of dietary intervention, but subjects were” |
|  | **Blinding of outcome assessment (detection bias)** | High risk | No blinding of outcome assessment, and the outcome measurement is likely to be influenced by lack of blinding;  Quote: “The investigator was not blinded as to the kind of dietary intervention, but subjects were” |
|  | **Incomplete outcome data (attrition bias)** | Low risk | Missing outcome data balanced in numbers across intervention groups, with similar reasons for missing data across group  Quote: “49 out of 60 subjects completed the study: 26 in CHCD and 23 in MHCD” Intention to treat analysis was not performed. |
|  | **Selective reporting (reporting bias)** | Unclear risk | Insufficient information to permit judgement |
|  | **Other bias** | Unclear risk | Insufficient information to permit judgement |
| Moeller 2019 | **Random sequence generation (selection bias)** | Low risk | The investigators describe a random component in the sequence generation process  Quote: “a computer-randomization was performed in blocks of two and four, blinded to authors” |
|  | **Allocation concealment (selection bias)** | High risk | Participants or investigators enrolling participants could possibly foresee assignments and thus introduce selection bias |
|  | **Blinding of participants and personnel (performance bias)** | Unclear risk | Insufficient information to permit judgement |
|  | **Blinding of outcome assessment (detection bias)** | Unclear risk | Insufficient information to permit judgement |
|  | **Incomplete outcome data (attrition bias)** | Low risk | Missing outcome data balanced in numbers across intervention groups, with similar reasons for missing data across group  The attrition rate was 5/19 in the MI group and 4/18 in the SC group. Intention-to-treat analysis was not performed. |
|  | **Selective reporting (reporting bias)** | Unclear risk | Insufficient information to permit judgement |
|  | **Other bias** | Unclear risk | Insufficient information to permit judgement |
| Moini 2015 | **Random sequence generation (selection bias)** | Low risk | The investigators describe a random component in the sequence generation process  Quote: “we used the randomization table method to divide the patients into two groups. A” |
|  | **Allocation concealment (selection bias)** | Low risk | Participants and investigators enrolling participants could not foresee assignment |
|  | **Blinding of participants and personnel (performance bias)** | Low risk | Blinding of participants and key study personnel ensured, and unlikely that the blinding could have been broken  Quote: “double-blind design” |
|  | **Blinding of outcome assessment (detection bias)** | Low risk | Blinding of outcome assessment ensured, and unlikely that the blinding could have been broken.  Quote: “A member of the study team who was blinded to both groups visited each participant monthly” |
|  | **Incomplete outcome data (attrition bias)** | Low risk | Missing outcome data balanced in numbers across intervention groups, with similar reasons for missing data across group  Quote: “In the intervention (n = 50) and control groups (n = 50), the same numbers of participants completed the study (n = 43 for both groups).” |
|  | **Selective reporting (reporting bias)** | Unclear risk | Insufficient information to permit judgement |
|  | **Other bias** | Unclear risk | Insufficient information to permit judgement |
| Moran 2003  Moran 2010 | **Random sequence generation (selection bias)** | Unclear risk | Insufficient information about the sequence generation process to permit judgement |
|  | **Allocation concealment (selection bias)** | Unclear risk | Insufficient information to permit judgement |
|  | **Blinding of participants and personnel (performance bias)** | High risk | No blinding or incomplete blinding, and the outcome is likely to be influenced by lack of blinding  Quote: “Subjects and investigators were not blinded as to the dietary intervention.” |
|  | **Blinding of outcome assessment (detection bias)** | High risk | No blinding of outcome assessment, and the outcome measurement is likely to be influenced by lack of blinding;  Quote: “Subjects and investigators were not blinded as to the dietary intervention.” |
|  | **Incomplete outcome data (attrition bias)** | Low risk | Missing outcome data balanced in numbers across intervention groups, with similar reasons for missing data across group  The attrition rate was 8/22 in the LP diet group and 9/23 in the HP diet group. Intention-to-treat analysis was not performed. |
|  | **Selective reporting (reporting bias)** | Low risk | All pre-specified outcomes are reported. |
|  | **Other bias** | Unclear risk | Insufficient information to permit judgement |
| Moran 2006 | **Random sequence generation (selection bias)** | Low risk | The investigators describe a random component in the sequence generation process  Quote: “randomized to the CC or FC protocol before study commencement by an independent observer using the computer program CLINSTAT” |
|  | **Allocation concealment (selection bias)** | Low risk | Participants and investigators enrolling participants could not foresee assignment |
|  | **Blinding of participants and personnel (performance bias)** | Unclear risk | Insufficient information to permit judgement |
|  | **Blinding of outcome assessment (detection bias)** | Unclear risk | Insufficient information to permit judgement |
|  | **Incomplete outcome data (attrition bias)** | Low risk | Missing outcome data balanced in numbers across intervention groups, with similar reasons for missing data across group  The attrition rate was 4/18 in the CC group and 7/16 in the FC group. Intention-to-treat analysis was performed. |
|  | **Selective reporting (reporting bias)** | Unclear risk | Insufficient information to permit judgement |
|  | **Other bias** | Unclear risk | Insufficient information to permit judgement |
| Munir 2018 | **Random sequence generation (selection bias)** | Low risk | The investigators describe a random component in the sequence generation process  Quote: “Randomization was done through separate randomization tables based on group using drug and other group only lifestyle changes.” |
|  | **Allocation concealment (selection bias)** | Low risk | Participants and investigators enrolling participants could not foresee assignment |
|  | **Blinding of participants and personnel (performance bias)** | High risk | No blinding or incomplete blinding, and the outcome is likely to be influenced by lack of blinding  Quote: “it could not be double blind” |
|  | **Blinding of outcome assessment (detection bias)** | Unclear risk | Insufficient information to permit judgement |
|  | **Incomplete outcome data (attrition bias)** | Unclear risk | Insufficient information to permit judgement |
|  | **Selective reporting (reporting bias)** | Unclear risk | Insufficient information to permit judgement |
|  | **Other bias** | Unclear risk | Insufficient information to permit judgement |
| Nybacka 2011  Nybacka 2013  Nybacka 2017 | **Random sequence generation (selection bias)** | Low risk | The investigators describe a random component in the sequence generation process  Quote: “The randomization was performed using the permuted-block randomization method, with 10 blocks and a block size of six.” |
|  | **Allocation concealment (selection bias)** | High risk | Participants or investigators enrolling participants could possibly foresee assignments and thus introduce selection bias |
|  | **Blinding of participants and personnel (performance bias)** | High risk | No blinding or incomplete blinding, and the outcome is likely to be influenced by lack of blinding |
|  | **Blinding of outcome assessment (detection bias)** | Unclear risk | Insufficient information to permit judgement |
|  | **Incomplete outcome data (attrition bias)** | Unclear risk | Insufficient information to permit judgement |
|  | **Selective reporting (reporting bias)** | Unclear risk | Insufficient information to permit judgement |
|  | **Other bias** | Unclear risk | Insufficient information to permit judgement |
| Oberg 2019 | **Random sequence generation (selection bias)** | Low risk | The investigators describe a random component in the sequence generation process  Quote: “The randomization was carried out with blocks of eight patients using the program SAS Systems 9.1 (SAS Institute Inc, Cary, NC, USA).” |
|  | **Allocation concealment (selection bias)** | High risk | Participants or investigators enrolling participants could possibly foresee assignments and thus introduce selection bias |
|  | **Blinding of participants and personnel (performance bias)** | High risk | No blinding or incomplete blinding, and the outcome is likely to be influenced by lack of blinding  Quote: “Due to the apparent difference in the interventions, neither patients nor care providers were blinded to the allocation.” |
|  | **Blinding of outcome assessment (detection bias)** | High risk | No blinding of outcome assessment, and the outcome measurement is likely to be influenced by lack of blinding  Quote: “Due to the apparent difference in the interventions, neither patients nor care providers were blinded to the allocation.” |
|  | **Incomplete outcome data (attrition bias)** | Low risk | Missing outcome data balanced in numbers across intervention groups, with similar reasons for missing data across group  The attrition rate was 21/68 on completion of the trial. Intention-to-treat analysis was performed. |
|  | **Selective reporting (reporting bias)** | Low risk | All pre-specified outcomes are reported. |
|  | **Other bias** | Unclear risk | Insufficient information to permit judgement |
| Panduveric 2023 | **Random sequence generation (selection bias)** | Unclear risk | Insufficient information about the sequence generation process to permit judgement |
|  | **Allocation concealment (selection bias)** | Unclear risk | Insufficient information to permit judgement |
|  | **Blinding of participants and personnel (performance bias)** | High risk | No blinding or incomplete blinding, and the outcome is likely to be influenced by lack of blinding |
|  | **Blinding of outcome assessment (detection bias)** | Unclear risk | Insufficient information to permit judgement |
|  | **Incomplete outcome data (attrition bias)** | Low risk | Missing outcome data balanced in numbers across intervention groups, with similar reasons for missing data across group |
|  | **Selective reporting (reporting bias)** | Low risk | All pre-specified outcomes are reported. |
|  | **Other bias** | Unclear risk | Insufficient information to permit judgement |
| Samarasinghe 2024 | **Random sequence generation (selection bias)** | Low risk | The investigators describe a random component in the sequence generation process  Quote: “randomly assigned using a computer- generated random sequence at a 1:1 ratio” |
|  | **Allocation concealment (selection bias)** | Low risk | Participants and investigators enrolling participants could not foresee assignment |
|  | **Blinding of participants and personnel (performance bias)** | High risk | No blinding or incomplete blinding, and the outcome is likely to be influenced by lack of blinding |
|  | **Blinding of outcome assessment (detection bias)** | High risk | No blinding of outcome assessment, and the outcome measurement is likely to be influenced by lack of blinding |
|  | **Incomplete outcome data (attrition bias)** | Unclear risk | Insufficient information to permit judgement |
|  | **Selective reporting (reporting bias)** | Low risk | All pre-specified outcomes are reported. |
|  | **Other bias** | Low risk | The study appears to be free of other sources of bias. |
| Stamets 2004 | **Random sequence generation (selection bias)** | Low risk | The investigators describe a random component in the sequence generation process  Quote: “A random number table was used to randomize participants to one of two energy-restricted diets” |
|  | **Allocation concealment (selection bias)** | Low risk | Participants and investigators enrolling participants could not foresee assignment |
|  | **Blinding of participants and personnel (performance bias)** | High risk | No blinding or incomplete blinding, and the outcome is likely to be influenced by lack of blinding |
|  | **Blinding of outcome assessment (detection bias)** | Unclear risk | Insufficient information to permit judgement |
|  | **Incomplete outcome data (attrition bias)** | Low risk | Missing outcome data balanced in numbers across intervention groups, with similar reasons for missing data across group  The attrition rate was 4/17 for the high protein diet and 5/18 for the high carbohydrate diet. Intention-to-treat analysis was not performed. |
|  | **Selective reporting (reporting bias)** | Unclear risk | Insufficient information to permit judgement |
|  | **Other bias** | Unclear risk | Insufficient information to permit judgement |
| Veena Kirthika 2019 | **Random sequence generation (selection bias)** | Low risk | The investigators describe a random component in the sequence generation process  Quote: “recruited and divided into two groups by simple random sampling (random number tables from standard statistics book) to participate in this pilot study.” |
|  | **Allocation concealment (selection bias)** | Low risk | Participants and investigators enrolling participants could not foresee assignment |
|  | **Blinding of participants and personnel (performance bias)** | High risk | No blinding or incomplete blinding, and the outcome is likely to be influenced by lack of blinding |
|  | **Blinding of outcome assessment (detection bias)** | Unclear risk | Insufficient information to permit judgement |
|  | **Incomplete outcome data (attrition bias)** | Unclear risk | Insufficient information to permit judgement |
|  | **Selective reporting (reporting bias)** | Unclear risk | Insufficient information to permit judgement |
|  | **Other bias** | Unclear risk | Insufficient information to permit judgement |
| Vigorito 2007 | **Random sequence generation (selection bias)** | Unclear risk | Insufficient information about the sequence generation process to permit judgement |
|  | **Allocation concealment (selection bias)** | Unclear risk | Insufficient information to permit judgement |
|  | **Blinding of participants and personnel (performance bias)** | High risk | No blinding or incomplete blinding, and the outcome is likely to be influenced by lack of blinding |
|  | **Blinding of outcome assessment (detection bias)** | Low risk | Blinding of outcome assessment ensured, and unlikely that the blinding could have been broken  Quote: “All clinical assessments were performed by the same physician who was blinded to the patient allocation into the study protocol.” |
|  | **Incomplete outcome data (attrition bias)** | Low risk | No missing outcome data  Quote: “All subjects completed the study protocol.” |
|  | **Selective reporting (reporting bias)** | Unclear risk | Insufficient information to permit judgement |
|  | **Other bias** | Unclear risk | Insufficient information to permit judgement |
| Vosnakis 2012 | **Random sequence generation (selection bias)** | Unclear risk | Insufficient information about the sequence generation process to permit judgement |
|  | **Allocation concealment (selection bias)** | Unclear risk | Insufficient information to permit judgement |
|  | **Blinding of participants and personnel (performance bias)** | High risk | No blinding or incomplete blinding, and the outcome is likely to be influenced by lack of blinding |
|  | **Blinding of outcome assessment (detection bias)** | Unclear risk | Insufficient information to permit judgement |
|  | **Incomplete outcome data (attrition bias)** | High risk | Reason for missing outcome data likely to be related to true outcome, with either imbalance in numbers or reasons for missing data across intervention groups  Quote: “The initial randomization was 2–1 but ended in a 3–1 ratio due to the withdrawal of 19 patients from the group that received only hypocaloric diet plus exercise. These patients were lost to follow up, while no drop outs were recorded in the group treated with sibutramine.” Attrition bias was not performed. |
|  | **Selective reporting (reporting bias)** | Unclear risk | Insufficient information to permit judgement |
|  | **Other bias** | Unclear risk | Insufficient information to permit judgement |
