# Supplementary material for: Pre‐conception weight loss interventions in women with polycystic ovary syndrome and the effect on perinatal outcomes: A quantitative synthesis of surrogate outcomes
Source: Diabetes Obes Metab. 2025 Oct 1;27(12):7158–79. doi: 10.1111/dom.70116 (PMC12587233; doi:10.1111/dom.70116)
Supplement: Supplementary file 5 — Data S5. Supporting Information. [file DOM-27-7158-s004.docx]

**Supplementary Material 5**

***Table 5. Certainty of evidence of primary outcomes***

| **Outcome** | **No. of participants (studies)** | **Relative effect (95% CI)** | **Absolute effect** | **Certainty of evidence (GRADE)** | **Reasons for downgrade or upgrade** |
| --- | --- | --- | --- | --- | --- |
| **Pregnancy rate** | 111 (1 RCT) | RR 1.39 (0.80 to 2.41) | 23.3–26.7% (intervention) vs 16.7% (control) | Low | Downgraded for imprecision (wide CI) and indirectness (not primary endpoint) |
| **Live birth rate** | 111 (1 RCT) | Not statistically different | Not reported numerically | Low | Downgraded for imprecision and outcome not being a primary endpoint |
