# Supplementary material for: Pre‐conception weight loss interventions in women with polycystic ovary syndrome and the effect on perinatal outcomes: A quantitative synthesis of surrogate outcomes
Source: Diabetes Obes Metab. 2025 Oct 1;27(12):7158–79. doi: 10.1111/dom.70116 (PMC12587233; doi:10.1111/dom.70116)
Supplement: Supplementary file 6 — Data S6. Supporting Information. [file DOM-27-7158-s001.docx]

**Supplementary Material 5**

***Table 5.*** *Reasons for exclusion of studies*

| Title | Reason for Exclusion |
| --- | --- |
| The effects of lifestyle guidance and mental health care on improving the carbohydrate metabolism and enhancing the pregnancy rate in obese pcos patients mental health care Chen H.; Wang Q. | Study design |
| Clinical, metabolic and endocrine parameters in response to metformin in obese women with polycystic ovary syndrome: a randomized, double-blind and placebo-controlled trial Chou KH; von Eye Corleta H; Capp E; Spritzer PM | Patient population (age 16-22) |
| Time Restricted Eating for the Treatment of Polycystic Ovary Syndrome: A Pilot Study Cienfuegos S.; Ezpeleta M.; Gonzalez F.; Lin S.; Varady K. | Abstract |
| Favorable changes in phenotype expression and androgens in women with pcos due to weight loss in a three-component lifestyle intervention program De Loos, AD; Timman, R; Jiskoot, G; Beerthuizen, A; Busschbach, J; Laven, J | Abstract |
| Pregnancy Rate and Outcomes Following a Randomized Controlled Three-component Lifestyle Intervention in Women with PCOS Dietz de Loos A.; Jiskoot G.; Louwers Y.; Beerthuizen A.; Busschbach J.; Laven J. | Abstract |
| Favorable changes in characteristics, phenotype and androgens as result of weight loss in a randomised controlled three-component lifestyle intervention in women with PCOS Dietz de Loos A; Timman R; Jiskoot G; Beerthuizen A; Van Busschbach J; Laven J | Protocol |
| Effects of three medical nutrition therapies for weight loss on metabolic parameters and androgen level in overweight/obese patients with polycystic ovary syndrome Dou, P; Zhang, TT; Xu, Y; Xue, Q; Shang, J; Yang, XL | Patient population (BMI) |
| Effects of raw red onion consumption on metabolic features in overweight or obese women with polycystic ovary syndrome: a randomized controlled clinical trial Ebrahimi-Mamaghani M; Saghafi-Asl M; Pirouzpanah S; Asghari-Jafarabadi M | intervention (Onion) |
| Effect of liraglutide on atrial natriuretic peptide, adrenomedullin, and copeptin in PCOS Frossing S; Nylander M; Kistorp C; Skouby SO; Faber J | Patient population (BMI > 25 and/or Insulin resistance) |
| A randomised trial comparing a low carbohydrate diet and nutrient-balanced low glycaemic index diet on body weight, hyperandrogenism and cardiovascular risk factors in women with polycystic ovary syndrome (PCOS) Ghosh D; Murphy C; Elsheikh M | Abstract |
| Effects of multidimensional life management on healthy behavior in polycystic ovary syndrome patients: A randomized controlled trial Guo, Y; Liu, Y; Yan, X; Ding, R; Wang, L | Patient population (BMI) |
| Reproductive hormonal changes following two types of hypocaloric diets in overweight and obese polycystic ovary syndrome women Hamayeli Mehrabani H; Tahbaz F; Salehpour S; Hedayati M; Amiri Z; Ghassemi A | Language (Not English) |
| The effectiveness of high intensity intermittent training on metabolic, reproductive and mental health in women with polycystic ovary syndrome: study protocol for the iHIT- randomised controlled trial. Hiam, Danielle; Patten, Rhiannon; Gibson-Helm, Melanie; Moreno-Asso, Alba; McIlvenna, Luke; Levinger, Itamar; Harrison, Cheryce; Moran, Lisa J; Joham, Anju; Parker, Alex; Shorakae, Soulmaz; Simar, David; Stepto, Nigel | Protocol |
| The effect of 6-month nutritional intervention on the anthropometric, biochemical, and reproductive profile of Lebanese women with Polycystic ovarian syndrome Hmedeh C; El Iskandarni S; Tawfik I | Abstract |
| The effect of 6 months weight -loss/maintenance on anthropometric, biochemical and psychological profile in Lebanese PCOS women: a prospective randomised control study Hmedeh C; Ghazeeri G; Tinworth L; Tewfik I | Study design |
| The effect of 6 months weight -loss/maintenance on anthropometric, biochemical and psychological profile in Lebanese PCOS women: a prospective randomised control study Hmedeh C; Ghazeeri G; Tinworth L; Tewfik I | Study design |
| A randomized, placebo-controlled trial of metformin and lifestyle management in overweight women with polycystic ovary syndrome: a pilot study Hoeger K; Guzick D; Kochman L; Craig K; Miller R | Abstract |
| Nutritional education (face-to-face and video instruction) for polycystic ovary syndrome results in greater reduction in bmi and hemoglobin a1c than caloric restriction, exercise and metformin Jackson B.; Kishan R.; Mullins C.; Mathew M.; Kim S.; Huang J.-C.; Phy J.L. | Abstract |
| Short-term effectiveness of low dose liraglutide in combination with metformin vs. high dose liraglutide alone in treatment of obesity Jensterle M; Kravos N; Janez A | Abstract |
| Once-weekly semaglutide delays a late phase gastric emptying of solid meal measured by repeated scintigraphic imaging in obese women with PCOS Jensterle M.; Ferjan S.; Lezaic L.; Socan A.; Zaletel K.; Janez A. | Abstract |
| Effects on body weight and waist/hip circumference during a 1-year three component lifestyle RCT in obese PCOS women Jiskoot L; Timman R; Beerthuizen A; Busschbach J; Laven J | Abstract |
| Effects on body weight and waist/hip circumference during a 1-year three-component lifestyle RCT in obese PCOS women Jiskoot L; Timman R; Beerthuizen A; Busschbach J; Laven J | Abstract |
| Effects on body weight of a 1-year three-component lifestyle RCT in obese PCOS women Jiskoot G; Benneheij S; Beerthuizen A; Timman R; Busschbach J; Laven J | Duplicate |
| The Female Health Dietary Intervention Study Treatment of PCOS (Polycystic Ovary Syndrome)in Morbidly Obese Women - A Randomized Controlled Prospective Dietary Intervention Study / 2008;(): | Protocol |
| Effects of low calorie diets based on crisp bread or liquid meal replacements on metabolic syndrome in morbidly obese women with polycystic ovarian syndrome (PCOS). A randomised controlled trial Johnson LKJ; Tanbo TT; Holven KBH; Hjelmesæth JH | Abstract |
| The effects of DASH diet on lipid profiles and biomarkers of oxidative stress in overweight and obese women with polycystic ovary syndrome: a randomised clinical trial Karamali M; Samimi M; Bahmani F; Foroozanfard F; Esmaillzadeh A | Abstract |
| A Randomized Controlled Trial of a Lifestyle Intervention with Longitudinal Follow up on Ovarian Dysmorphology, Hyperandrogenism, and Menstrual Irregularity in Women with Polycystic Ovary Syndrome Kazemi, M; Pierson, RA; McBreairty, LE; Chilibeck, PD; Zello, GA; Chizen, DR | Patient population (BMI) |
| Polycystic ovarian disease: Impact of metformin therapy Kore SJ; Lakhotia S; Parikh M; Kulkarni V; Ambiye VR | Not available |
| Isocaloric diet is as effective as the hypocaloric diet in ameliorating symptoms in PCOS patients Kulshreshtha B.; Sharma N.; Pant S.; Sharma L.; Pahuja B.; Singh P. | Patient population (BMI) |
| Isocaloric diet is as effective as the hypocaloric diet in ameliorating symptoms in PCOS patients Kulshreshtha, B; Sharma, N; Pant, S; Sharma, L; Pahuja, B; Singh, P | Patient population (BMI) |
| Effect of sibutramine on weight reduction in women with polycystic ovary syndrome: a randomized, double-blind, placebo-controlled trial Lindholm A; Bixo M; Bjorn I; Wolner-Hanssen P; Eliasson M; Larsson A; Johnson O; Poromaa IS | Abstract |
| Efficacy of exenatide on weight loss, metabolic parameters and pregnancy in overweight/obese polycystic ovary syndrome (in press) Liu, X; Zhang, Y; Zheng, Sy; Lin, R; Xie, Yj; Chen, H; Zheng, Yx; Liu, E; Chen, L; Yan, Jh; et al. | Patient population (BMI) |
| Short-term monotherapy with exenatide is superior to metformin in improving pregnancy, fat distribution, and metabolic parameters in overweight/obese PCOS women Liu X; Zhang Y; Zheng SY; Lin R; Xie YJ; Chen H; Zheng YX; Liu E; Chen L; Yan JH; Mai TT; Gong Y | Patient population (BMI) |
| Efficacy of exenatide on weight loss, metabolic parameters and pregnancy in overweight/obese polycystic ovary syndrome Liu X; Zhang Y; Zheng SY; Lin R; Xie YJ; Chen H; Zheng YX; Liu E; Chen L; Yan JH; Xu W; Mai TT; Gong Y | Patient population (BMI) |
| Short-term combined treatment with exenatide and metformin for overweight/obese women with polycystic ovary syndrome Ma RL; Deng Y; Wang YF; Zhu SY; Ding XS; Sun AJ | Intervention (COCP) |
| The effect of cognitive behavioral therapy on depression and anxiety of women with polycystic ovary syndrome: a randomized controlled trial. Majidzadeh, Sheida; Mirghafourvand, Mojgan; Farvareshi, Mahmoud; Yavarikia, Parisa | Patient population (BMI) |
| Effects of aerobic exercise on plasma lipoproteins in overweight and obese women with polycystic ovary syndrome Mardanian F.; Taghian F.; Abazar E. | Abstract |
| Mediterranean Diet Combined With a Low-Carbohydrate Dietary Pattern in the Treatment of Overweight Polycystic Ovary Syndrome Patients. Mei, Shanshan; Ding, Jie; Wang, Kaili; Ni, Zhexin; Yu, Jin | Patient population (BMI) |
| Comparison of the effects of a diet and physical activity trial in obese women with polycystic ovary syndrome Mirfeizi M. | Abstract |
| Effects of eight-week high-intensity interval training on some metabolic, hormonal and cardiovascular indices in women with PCOS: a randomized controlled trail Mohammadi S.; Monazzami A.; alavimilani S. | Patient population (BMI) |
| Plasma Anti-Mullerian Hormone (AMH) levels correlate and predict the response to lifestyle intervention in polycystic ovarian syndrome Nyback A; Fabri F; Hellstrom PM; Hirschberg AL; Stahle A | Abstract |
| Metabolic and cardiopulmonary effects of detraining after a structured exercise training programme in young PCOS women Orio F; Giallauria F; Palomba S; Manguso F; Orio M; Tafuri D; Lombardi G; Carmina E; Colao A; Vigorito C | Study design |
| Effects of an "Internet +" Multidisciplinary Management Model Oriented by Nurse Specialists on Obesity Combined with Polycystic Ovary Syndrome Ou Y.; Wu L.; Liu L.; Ye X.; Chen Y.; Yuan H. | Patient population (BMI) |
| High-intensity training elicits greater improvements in cardio-metabolic and reproductive outcomes than moderate-intensity training in women with polycystic ovary syndrome: a randomized clinical trial Patten RK; McIlvenna LC; Levinger I; Garnham AP; Shorakae S; Parker AG; McAinch AJ; Rodgers RJ; Hiam D; Moreno-Asso A; Stepto NK | Patient population (Physical inactivity) |
| Acarbose in obese patients with polycystic ovarian syndrome: a double-blind, randomized, placebo-controlled study Penna, IA; Canella, PR; Reis, RM; Silva de Sá, MF; Ferriani, RA | Patient population (Insulin resistance) |
| Cardiovascular autonomic modulation differences between moderate-intensity continuous and high-intensity interval aerobic training in women with PCOS: A randomized trial. Philbois, Stella V; Ribeiro, Victor B; Tank, Jens; Dos Reis, Rosana Maria; Gerlach, Darius A; Souza, Hugo C D | Patient population (BMI) |
| The role of weight loss and metformin in the improvement of menstrual function in overweight and obese women with polycystic ovary sindrome Popova P; Ryasantseva E; Zazerskaya I; Ivanova L; Grineva E | Abstract |
| Effects of orlistat on serum androgen levels among iranian obese women with polycystic ovarian syndrome Salehpour, S; Hosseini, S; Nazari, L; Saharkhiz, N; Zademodarres, S | Study design |
| Evaluation of changes in levels of hyperandrogenism, hirsutism and menstrual regulation after a period of aquatic high intensity interval training in women with polycystic ovary syndrome Samadi Z; Bambaeichi E; Valiani M; Shahshahan Z | Patient population (Physical inactivity and insulin resistance) |
| Usage of mobile health interventions among overweight/obese PCOS patients undergoing assisted reproductive technology treatment during the COVID-19 pandemic. Sang, Meiying; Wu, Qiong; Tao, Yuanyuan; Huang, Feifei; Lu, Lianlian; Zhou, Wenting; Li, Aixiang; Bai, Shun | Patient population (BMI) |
| Effects of high-intensity interval training in combination with detraining on mental health in women with polycystic ovary syndrome: A randomized controlled trial Santos I.K.; Pichini G.S.; Daniel d. Ferreira C.; Dantas P.B.; Browne R.A.V.; de Queiros V.; Soares G.M.; Goncalves A.K.; Cabral B.G.; Maranhao T.M.O.; Dantas P.M.S. | Patient population (BMI) |
| Efficacy, Feasibility and Acceptability of a Mediterranean Diet Intervention on Hormonal, Metabolic and Anthropometric Measures in Overweight and Obese Women with Polycystic Ovary Syndrome: study Protocol Scannell N; Moran L; Mantzioris E; Cowan S; Villani A | Protocol |
| Short-term combined treatment with liraglutide and metformin leads to significant weight loss in obese women with polycystic ovary syndrome and previous poor response to metformin Sever M.J.; Kocjan T.; Pfeifer M.; Janez A. | Patient population (Previous poor response to metformin) |
| Influence of lifestyle guidance and mental care on internal secretion, carbohydrate metabolism and pregnancy in obese patients with polycystic ovary syndrome Song J.; Wang H. | Study design |
| Effects of training intensity on polycystic ovary syndrome markers: a randomized-controlled trial Sonntag, B | Patient population (Physical inactivity) |
| The effect of diet and exercise in women with polycystic ovary syndrome Sweatt K; Ovalle F; Azziz R; Gower B | Abstract |
| The effect of diet and exercise on vascular function in overweight and obese women with polycystic ovary syndrome Thomson RL; Brinkworth GD; Noakes M; Clifton PM; Norman RJ; Buckley JD | Abstract |
| The effect of diet and exercise on markers of endothelial function in overweight and obese women with polycystic ovary syndrome Thomson RL; Brinkworth GD; Noakes M; Clifton PM; Norman RJ; Buckley JD | Patient population (Sedentary) |
| The effect of a hypocaloric diet with and without exercise training on body composition, cardiometabolic risk profile, and reproductive function in overweight and obese women with polycystic ovary syndrome Thomson RL; Buckley JD; Noakes M; Clifton PM; Norman RJ; Brinkworth GD | Patient population (Sedentary) |
| Effect of Wolly Workout in PCOS and PCOD Patients as Part of Management Tripathi P.P. | Patient population (BMI) |
| Short-term effect of beinaglutide combined with metformin versus metformin alone on weight loss and metabolic profiles in obese patients with polycystic ovary syndrome: a pilot randomized trial. Wen, Qing; Fang, Song; Liang, Yanjing; Tian, Yuting; Chen, Yiding; Yuan, Jun; Chen, Qiu | Patient population (BMI) |
| Effect of metformin versus metformin plus liraglutide on gonadal and metabolic profiles in overweight patients with polycystic ovary syndrome. Xing, Chuan; Zhao, Han; Zhang, Jiaqi; He, Bing | Patient population (BMI) |
| Effects of Metformin on Endocrine and Metabolic Parameters in Patients with Polycystic Ovary Syndrome Zahra M; Shah M; Ali A; Rahim R | Patient population (BMI) |
| Effect of using 5A's model for lifestyle counseling on psychological symptoms in women with polycystic ovary syndrome: a randomized field trial. ZareMobini, Fatemeh; Farajzadegan, Ziba; Kazemi, Ashraf; Salehi, Mehrdad | Patient population (BMI) |
| Effect of Dulaglutide plus Calorie-Restricted Diet vs. Calorie-Restricted Diet on Visceral Fat and Metabolic Profiles in Women with Polycystic Ovary Syndrome-A Randomized Controlled Trial Zhang Y.; Shao X.; Cai M.; Dilimulati D.; Qu S.; Zhang M. | Abstract |
| Effects of liraglutide on leptin promoter methylation in ovarian granulosa cells of patients with polycystic ovary syndrome and obesity Zhao H.; Guo Y. | Patient population (BMI) |
| Effects of orlistat and metformin on metabolism and gonadal function in overweight or obese patients with polycystic ovary syndrome Zhao YX; Wang LJ; Gong FY; Pan H; Miao H; Duan L; Yang HB; Zhu HJ | Language (Not English) |
| Efficacy of internet-based intensive weight management or exenatide treatment on weight loss and metabolism in overweight/obese pcos Zheng S.; Zhang Y.; Li R.; Xue J.; Shen H. | Abstract |
