# Supplementary material for: Pre‐conception weight loss interventions in women with polycystic ovary syndrome and the effect on perinatal outcomes: A quantitative synthesis of surrogate outcomes
Source: Diabetes Obes Metab. 2025 Oct 1;27(12):7158–79. doi: 10.1111/dom.70116 (PMC12587233; doi:10.1111/dom.70116)
Supplement: Supplementary file 7 — Data S7. Supporting Information [file DOM-27-7158-s002.docx]

|  | Asemi 2014 (25), Asemi 2015 (26) | Atiomo 2009 (30) | Azadi-Yazdi 2017 (27) | Bruno 2007 (59) | Cincione 2023 (44) | De Loos 2021 (18), De Loos 2022 (17), De Loos 2023 (16), Jiskoot 2020 (19) | Deshmukh (45) | Elkind-Hirsch 2022 (34) | Esfahanian 2013 (31) | Florakis 2008 (57) | Foroozanfard 2017 (28) | Gan 2023 (60) | Ghandi 2011 (37) | Harborne 2005 (36) | Hoeger 2004 (32) | Jensterle 2015 (38) | Jensterle 2016 (61) | Jensterle 2017 (62) | Jensterle 2023 (63) | Johnson 2015 (46) | Kasim Karakas 2009 (47) | Lee 2023 (20) | Lindholm 2008 (33) | Mehrabani 2012 (48) | Moeller 2019 (21) | Moini 2015 (58) | Moran 2010 (49), Moran 2003 (24) | Moran 2006 (29) | Munir 2018 (35) | Nybacka 2011 (50), Nybacka 2013 (51), Nybacka 2017 (52) | Oberg 2019 (22) | Pandurevic 2023 (53) | Samarasinghe 2024 (39) | Stamets 2004 (54) | Veena Kirthika 2019 (56) | Vigorito 2007 (23) | Vosnakis 2012 (55) |
| --- | --- | --- | --- | --- | --- | --- | --- | --- | --- | --- | --- | --- | --- | --- | --- | --- | --- | --- | --- | --- | --- | --- | --- | --- | --- | --- | --- | --- | --- | --- | --- | --- | --- | --- | --- | --- | --- |
| Brief Name | Y | Y | Y | Y | Y | Y | Y | Y | Y | Y | Y | Y | Y | Y | Y | Y | Y | Y | Y | Y | Y | Y | Y | Y | Y | Y | Y | Y | Y | Y | Y | Y | Y | Y | Y | Y | Y |
| Why | Y | Y | Y | Y | Y | Y | Y | Y | Y | Y | Y | Y | Y | Y | Y | Y | Y | Y | Y | Y | Y | Y | Y | Y | Y | Y | Y | Y | Y | Y | Y | Y | Y | Y | Y | Y | Y |
| What Materials | Y | Y | Y | Y | Y | Y | Y | Y | Y | Y | Y | Y | Y | Y | Y | Y | Y | Y | Y | Y | Y | Y | Y | Y | Y | Y | Y | Y | Y | Y | Y | Y | Y | Y | Y | Y | Y |
| What Procedures | Y | Y | Y | Y | Y | Y | Y | Y | Y | Y | Y | Y | Y | Y | Y | Y | Y | Y | Y | Y | Y | Y | Y | Y | Y | Y | Y | Y | Y | Y | Y | Y | Y | Y | Y | Y | Y |
| Who Provided | Y | Y | Y | ? | Y | Y | Y | Y | Y | Y | Y | Y | Y | Y | Y | Y | Y | Y | Y | Y | Y | Y | Y | Y | Y | Y | Y | Y | ? | Y | Y | Y | Y | Y | Y | Y | Y |
| How | Y | Y | Y | Y | Y | Y | Y | Y | Y | Y | Y | Y | Y | Y | Y | Y | Y | Y | Y | Y | Y | Y | Y | Y | Y | Y | Y | Y | Y | Y | Y | Y | Y | Y | Y | Y | Y |
| Where | Y | Y | Y | Y | Y | Y | Y | Y | Y | Y | Y | Y | Y | Y | Y | Y | Y | Y | Y | Y | Y | Y | Y | Y | Y | Y | Y | Y | Y | Y | Y | Y | Y | Y | Y | Y | Y |
| When and How Much | Y | Y | Y | Y | Y | Y | Y | Y | Y | Y | Y | Y | Y | Y | Y | Y | Y | Y | Y | Y | Y | Y | Y | Y | Y | Y | Y | Y | Y | Y | Y | Y | Y | Y | Y | Y | Y |
| Tailoring | N/A | N/A | N/A | N/A | N/A | N/A | N/A | N/A | N/A | N/A | N/A | N/A | N/A | N/A | N/A | N/A | N/A | N/A | N/A | N/A | N/A | N/A | N/A | N/A | N/A | N/A | N/A | N/A | N/A | N/A | N/A | N/A | N/A | N/A | N/A | N/A | N/A |
| Modifications | N/A | N/A | N/A | N/A | N/A | N/A | N/A | N/A | N/A | N/A | N/A | N/A | N/A | N/A | N/A | N/A | N/A | N/A | N/A | N/A | N/A | N/A | N/A | N/A | N/A | N/A | N/A | N/A | N/A | N/A | N/A | N/A | N/A | N/A | N/A | N/A | N/A |
| How Well Planned | Y | Y | Y | ? | Y | ? | Y | Y | Y | Y | Y | ? | ? | ? | Y | ? | ? | ? | ? | Y | Y | ? | Y | Y | ? | Y | Y | Y | ? | ? | Y | ? | ? | Y | ? | ? | ? |
| How Well Actual | Y | Y | Y | N/A | Y | N/A | Y | Y | Y | Y | Y | N/A | N/A | N/A | Y | N/A | N/A | N/A | N/A | Y | Y | N/A | Y | Y | N/A | Y | Y | Y | N/A | N/A | Y | N/A | N/A | Y | N/A | N/A | N/A |

**Supplementary Material 6**

***Table 6.*** *TIDieR Checklist of Interventions*
